# Revisiting the anticancer properties of phosphane(9-ribosylpurine-6-thiolato)gold(I) complexes and their 9H-purine precursors

**DOI:** 10.1007/s00775-022-01968-x

**Published:** 2022-10-16

**Authors:** Luisa Kober, Sebastian W. Schleser, Sofia I. Bär, Rainer Schobert

**Affiliations:** Organic Chemistry Laboratory, University of Bayreuth, Universitaetsstrasse 30, 95440 Bayreuth, Germany

**Keywords:** Anticancer compounds, Gold(I) complexes, Thioredoxin reductase (TrxR) inhibitors, Triorganophosphanes, Nucleosides, Auranofin

## Abstract

New mono- and di-nuclear thio-purine and thio-purine nucleoside gold(I) complexes were synthesized, characterized, and evaluated in vitro for biological activities in comparison to related known purine complexes. By combining known anti-tumoral thio-purines with R_3_PAu moieties as present in auranofin, complexes with enhanced effects and selectivities were obtained, which not only act as cytostatics, but also disrupt tumor-specific processes. Their *IC*_*50*_ values in cytotoxicity test with tumor cell lines ranged from three-digit nanomolar to single-digit micromolar, revealing a tentative structure–activity relationship (SAR). Both the residues R^2^ of the phosphane ligand and R^1^ at C2 of the pyrimidine ring had a significant impact on the cytotoxicity. In most cases, the introduction of a ribo-furanosyl group at N9 of the purine led to a distinctly more cytotoxic complex. Most complexes were more active against multi-drug-resistant tumor cells or such lacking functional p53 when compared to the respective untreated wild type cell lines. Some nucleoside complexes displayed an interesting dose-dependent dual mode of action regarding cell cycle arrest and DNA repair mechanism. Some phosphane(purine-6-thiolato)gold (I) complexes had a stronger inhibitory effect on the thioredoxin reductase (TrxR) and on the reactive oxygen species (ROS) generation in cancer cells than is typical of other gold complexes. They also led to DNA fragmentation and showed anti-angiogenic effects. Their stability under test conditions was demonstrated by ^77^Se NMR monitoring of an exemplary selenopurine complex.

**Graphical abstract:**

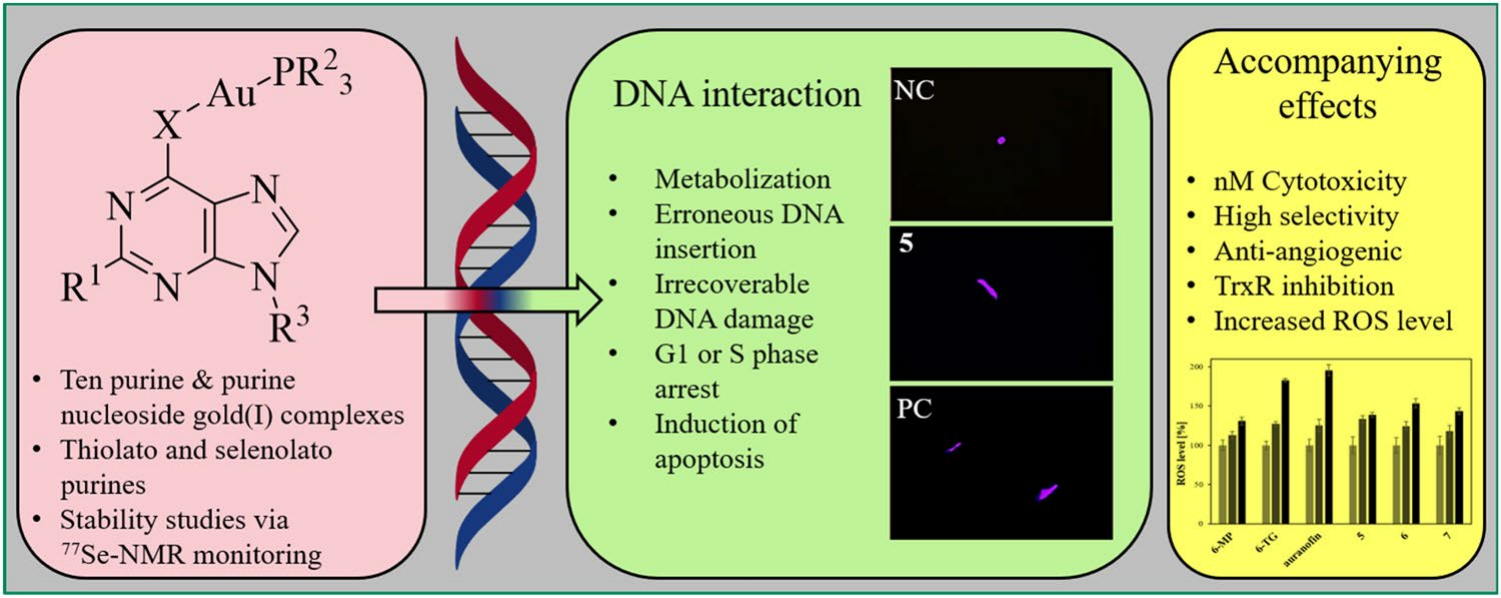

**Supplementary Information:**

The online version contains supplementary material available at 10.1007/s00775-022-01968-x.

## Introduction

Cisplatin has been and still is a mainstay in the chemotherapy of certain cancer entities, despite of its poor cancer selectivity, fast emergence of resistance, and frequent unwanted side effects. Later generation platinum drugs, such as oxaliplatin and carboplatin, while eliciting tumor resistance to a lesser degree than cisplatin, are still associated with severe side effects. For this reason, and in anticipation of a similar, platinum-like efficiency, the search for further metallo-drugs was extended to coordination and organometallic complexes of noble and other late transition metals. Although no such drug has been approved by the FDA for cancer treatment to date [1], auranofin is a successful example of a highly efficacious, multi-modal gold drug with few side effects. Initially designed and approved against rheumatoid arthritis [2], it has shown promising effects against a variety of cancer entities [3–5], in addition to antibacterial and antiviral properties [6]. Extensive SAR studies of structurally related compounds were carried out as early as 1986, in which the phosphane motif was found to be particularly relevant [7]. Recent studies supported these findings, in which the PEt_3_Au^+^ cation with various halides or thiols also exhibited sub-micro-molecular cytotoxicities against various human cancer cell lines [8, 9]. Since then, many gold(I) and gold(III) complexes with anti-tumor properties have been evaluated [10]. The choice of ligands and their lipophilicity, size and intrinsic toxicity turned out essential for their bioactivity and bio-distribution as too lipophilic phosphane gold(I) compounds e.g., [Au(dppe)_2_]^+^ tend to display significant hepatotoxicity [11]. One way to compensate for the lipophilicity of phosphanes is to use hydrophilic thiols as second ligands. Needless to say, that these complexes must be stable enough to reach their target, but not too unreactive to interfere with it [9]. The thio analogs of adenine and guanine proved to be particularly potent in this regard. 6-Mercaptopurine (6-MP) and 6-thioguanine (6-TG) are already well studied, clinically established antimetabolites with known modes of action [12]. By coordinating these thiopurines to various triorganylphosphane gold(I) fragments, Tiekink et al. could improve their beneficial effects. In fact, their complexes were cytotoxic even against human cancer cell lines not responsive to the underlying free purines [13–15]. Mechanistically, to exert their effect, they are first converted into their nucleotide analogs in vivo [16]. It is therefore unsurprising that their corresponding nucleosides, such as 6-methylmercaptopurine riboside (6-MMR), are also potent anticancer agents [13–15, 17].

Herein, we further investigate the thio-purine complexes of Tiekink et al. for their cytotoxicity and mechanism of action and we also take a look at the more effective complexes of the thio nucleosides 6-thioguanosine (6-TGS) and 6-thioinosin (6-TNS).

## Materials and methods

### Chemical synthesis

#### General

Reactions: all reactions with moisture-sensitive reagents were carried out under argon atmosphere in water-free solvents.

Solvents: unless stated otherwise, the solvents were purified and dried using standard methods.

Purchasable reagents: starting compounds were purchased from Sigma-Aldrich (St. Louis, United States), TCI (Tokyo, Japan), Merck (Darmstadt, Germany), abcr (Karlsruhe, Germany), Acros Organics (Fair Lawn, United States), VWR (Radnor, United States) and used without further purification. Nuclear Magnetic Resonance (NMR**)** spectra were measured using a Bruker (Bellerica, United States) DRX spectrometer at 500 MHz at ambient temperature. Chemical shifts are given in parts per million (*δ*). For ^1^H-NMR spectra the resonance signal of the residual proton of CDCl_3_ (*δ* = 7.26 ppm) or DMSO-d_6_ (*δ* = 2.50 ppm) was used as internal standard. For ^13^C-NMR spectra, the resonance signal of the carbon atom of CDCl_3_ (*δ* = 77.1 ppm) or DMSO-d_6_ (*δ* = 39.5 ppm) was used. ^1^H-NMR spectra were measured at 500 MHz, ^13^C-NMR spectra at 125 MHz, ^31^P-NMR spectra at 202 MHz and ^77^Se-NMR spectra at 95 MHz. For signal multiplicities, the following abbreviations were used: s = singlet, d = doublet, t = triplet, m = multiplet, dd = doublet of doublets, dt = doublet of triplets, dq = doublet of quartets. Coupling constants are given in Hz. Melting points were taken with an Electrothermal 9100 apparatus and are uncorrected. Mass spectra were recorded with a ThermoFisher Scientific (Waltham, United States) UPLC/Orbitrap MS system (HRMS-ESI). High-Performance Liquid Chromatography (HPLC): Analytical HPLC measurements were carried out on a Shimadzu Nexera XR with auto-sampler SIL-20A and a diode array detector SPD-M20A using the column Eurosphere II 100-3 C18 (150 × 4 mm) from Knauer GmbH (Berlin, Germany). Solvents used for the HPLC system featured the purity level ‘HPLC grade’. UV/Vis spectra were obtained using a Varioskan LUX Plate Reader—Multimode Microplate Reader from Thermo Fisher (Waltham, United States).

### General protocol for the synthesis of complexes 1–10

The respective thiol (1.00 eq.) and R_3_PAuCl (1.00 eq.) were suspended in EtOH (75 mL/mmol) and 200 µm KOH_(aq.)_ (1.00 eq.) was added dropwise. The suspension slowly started to dissolve and was left stirring for 24 h at rt. The solvent was evaporated and the residue was suspended in boiling acetone. After filtration, the solvent was evaporated to leave the complex as a colorless powder.

#### (1,7-Dihydro-6H-purine-6-thiolato) (triphenylphosphane) gold(I) (1)

84.0 mg (138 µmol, 68%) from 6-MP (34.4 mg, 202 µmol, 1.00 eq.), Ph_3_PAuCl (100 mg, 202 µmol, 1.00 eq.) and 200 µm KOH_(aq.)_ (1.01 mL, 202 µmol, 1.00 eq.) in EtOH (15 mL). m.p. 260 °C (decomp.); ^1^H NMR (500 MHz, DMSO-d_6_) *δ*_H_ = 13.3 (s, 1H, NH), 8.45 (s, 1H, H^ar^), 8.26 (s, 1H, H^ar^), 7.76–7.53 (m, 15H, H^ar^); ^13^C NMR (126 MHz, DMSO-d_6_) *δ*_C_ = 167.7 (s), 149.3 (s), 147.7 (s), 140.2 (s), 132.0 (d, *J*_*CP*_ = 14.1 Hz), 130.0 (d, *J*_*CP*_ = 2.7 Hz), 127.8 (s), 127.7 (d, *J*_*CP*_ = 11.4 Hz), 127.4 (s); ^31^P NMR (202 MHz, DMSO-d_6_) *δ*_P_ = 36.38; HRMS (ESI): m/z calculated for C_23_H_18_AuN_4_PS + H^+^ [M + H^+^]: 611.07336. Found: 611.07230.

#### (2-Amino-1,7-dihydro-6H-purine-6-thiolato) (triphenylphosphane) gold(I) (2)

68.0 mg (109 µmol, 54%) from 6-TG (33.8 mg, 202 µmol, 1.00 eq.), Ph_3_PAuCl (100 mg, 202 µmol, 1.00 eq.) and 200 µm KOH_(aq.)_ (1.01 mL, 202 µmol, 1.00 eq.) in EtOH (15 mL). m.p. 260 °C (decomp.); ^1^H NMR (500 MHz, DMSO-d_6_) *δ*_H_ = 12.4 (s, 1H, NH), 7.77 (s, 1H, H^ar^), 7.71–7.58 (m, 15H, H^ar^), 6.04 (s, NH_2_); ^13^C NMR (126 MHz, DMSO-d_6_) *δ*_C_ = 170.6 (s), 160.4 (s), 151.7 (s), 138.1 (s), 134.4 (d, *J*_*CP*_ = 14.1 Hz), 132.3 (d, *J*_*CP*_ = 2.7 Hz), 130.4 (s), 130.0 (d, *J*_*CP*_ = 11.4 Hz), 126.3 (s); ^31^P NMR (202 MHz, DMSO-d_6_) *δ*_P_ = 36.8; HRMS (ESI): m/z calculated for C_23_H_19_AuN_5_PS + H^+^ [M + H^+^]: 626.08425. Found: 626.08195.

#### (1,7-Dihydro-6H-purine-6-thiolato) (triethylphosphane) gold(I) (3)

40.0 mg (85.8 µmol, 30%) from 6-MP (48.6 mg, 285 µmol, 1.00 eq.), Et_3_PAuCl (100 mg, 285 µmol, 1.00 eq.) and 200 µm KOH_(aq.)_ (1.43 mL, 285 µmol, 1.00 eq.) in EtOH (15 mL). m.p. 240 °C (decomp.); ^1^H NMR (500 MHz, DMSO-d_6_) *δ*_H_ = 13.2 (s, 1H, NH), 8.38 (s, 1H, H^ar^), 8.26 (s, H^ar^), 1.91 (dt, *J* = 15.3 Hz, 7.3 Hz, 6H, CH_2_), 1.23–1.01 (m, 9H, CH_3_); ^13^C NMR (126 MHz, DMSO-d_6_) *δ*_C_ = 171.5 (s), 151.5 (s), 149.5 (s), 141.7 (s), 133.1 (s), 17.7 (d, *J*_CP_ = 34.1 Hz), 9.34 (s); ^31^P NMR (202 MHz, DMSO-d_6_) *δ*_P_ = 36.7; HRMS (ESI): m/z calculated for C_11_H_18_AuN_4_PS + H^+^ [M + H^+^]: 467.07336. Found: 467.07161.

#### (2-Amino-1,7-dihydro-6H-purine-6-thiolato) (triethylphosphane) gold(I) (4)

47.0 mg (97.7 µmol, 34%) from 6-TG (47.7 mg, 285 µmol, 1.00 eq.), Et_3_PAuCl (100 mg, 285 µmol, 1.00 eq.) and 200 µm KOH_(aq.)_ (1.43 mL, 285 µmol, 1.00 eq.) in EtOH (15 mL). m.p. 250 °C (decomp.); ^1^H NMR (500 MHz, DMSO-d_6_) *δ*_H_ = 12.3 (s, 1H, NH), 7.74 (s, 1H, H^ar^), 5.91 (s, NH_2_), 2.17–1.67 (m, 6H, CH_2_), 1.48–0.88 (m, 9H, CH_3_); ^13^C NMR (126 MHz, DMSO-d_6_) *δ*_C_ = 171.4 (s), 160.1 (s), 151.6 (s), 137.6 (s), 126.6 (s), 17.9 (d, *J*_CP_ = 33.6 Hz), 9.26 (s); ^31^P NMR (202 MHz, DMSO-d_6_) *δ*_P_ = 36.6; HRMS (ESI): m/z calculated for C_11_H_19_AuN_5_PS + H^+^ [M + H^+^]: 482.08425. Found: 482.08282.

#### (1,7-Dihydro-6H-purine-9-β-D-ribofuranosyl-6-thiolato) (triphenylphosphane) gold(I) (5)

53.0 mg (71.4 µmol, 50%) from 6-TNS (40.2 mg, 141 µmol, 1.00 eq.), Ph_3_PAuCl (70.0 mg, 141 µmol, 1.00 eq.) and 200 µm KOH_(aq.)_ (708 µL, 141 µmol, 1.00 eq.) in EtOH (15 mL). The crude product was purified by column chromatography (silica gel 60, CH_2_Cl_2_/MeOH 95:5). *R*_f_ = 0.63 (CH_2_Cl_2_/MeOH 9:1); m.p. 130 °C (decomp.); ^1^H NMR (500 MHz, DMSO-d_6_) *δ*_H_ = 8.56 (s, 1H, H^ar^), 8.49 (s, 1H, H^ar^), 7.76–7.58 (m, 15H, H^ar^), 5.97 (d, *J* = 5.5 Hz, 1H), 5.51 (d, *J* = 5.5 Hz, 1H), 5.28–5.17 (m, 2H), 4.59 (d, *J* = 5.5 Hz, 1H), 4.18 (q, *J* = 4.5 Hz, 1H), 3.97 (q, *J* = 3.7 Hz, 1H), 3.71 (dt, *J* = 12.1, 4.3 Hz, 1H), 3.58 (dd, *J* = 12.1, 6.4 Hz, 1H); ^13^C NMR (126 MHz, DMSO-d_6_) *δ*_C_ = 171.7 (s), 148.5 (s), 134.4 (d, *J*_*CP*_ = 14.1 Hz), 133.7 (s), 132.5 (d, *J*_CP_ = 2.8 Hz), 130.0 (d, *J*_*CP*_ = 11.4 Hz), 129.7 (s), 88.4 (s), 86.1 (s), 74.2 (s), 71.4 (s), 61.7 (s); ^31^P NMR (202 MHz, DMSO-d_6_) *δ*_P_ = 37.0; HRMS (ESI): m/z calculated for C_28_H_26_AuN_4_O_4_PS + H^+^ [M + H^+^]: 743.11561. Found: 743.11528.

#### (2-Amino-1,7-dihydro-6H-purine-9-β-D-ribofuranosyl-6-thiolato) (triphenylphosphane) gold(I) (6)

56.0 mg (73.9 µmol, 46%) from 6-TGS (48.4 mg, 161 µmol, 1.00 eq.), Ph_3_PAuCl (80.0 mg, 162 µmol, 1.00 eq.) and 200 µm KOH_(aq.)_ (809 µL, 162 µmol, 1.00 eq.) in EtOH (15 mL). The crude product was purified by column chromatography (silica gel 60, CH_2_Cl_2_/MeOH 95:5). *R*_f_ = 0.43 (CH_2_Cl_2_/MeOH 95:5); m.p. 230 °C (decomp.); ^1^H NMR (500 MHz, DMSO-d_6_) δ_H_ = 8.09 (s, 1H, H^ar^), 7.82–7.45 (m, 15H, H^ar^), 6.21 (d, *J* = 5.5 Hz, 2H, NH_2_), 5.80 (d, *J* = 5.6 Hz, 1H), 5.42 (d, *J* = 5.9 Hz, 1H), 5.22 (t, *J* = 5.6 Hz, 1H), 5.13 (d, *J* = 4.8 Hz, 1H) 4.48 (q, *J* = 5.6 Hz, 1H), 4.14 (q, *J* = 4.5 Hz, 1H), 3.91 (q, *J* = 3.6 Hz, 1H), 3.67 (dt, *J* = 12.0, 4.7 Hz, 1H), 3.56 (dd, *J* = 12.0, 6.2 Hz, 1H); ^13^C NMR (126 MHz, DMSO-d_6_) *δ*_C_ = 171.6 (s), 160.1 (s), 150.9 (s), 134.4 (d, *J*_*CP*_ = 13.7 Hz), 132.4 (s), 130.0 (d, *J*_*CP*_ = 11.4 Hz), 126.6 (s), 87.4 (s), 85.7 (s), 74.0 (s), 70.8 (s), 61.7 (s); ^31^P NMR (202 MHz, DMSO-d_6_) *δ*_P_ = 37.1; HRMS (ESI): m/z calculated for C_28_H_27_AuN_5_O_4_PS + H^+^ [M + H^+^]: 758.12651. Found: 758.12542.

#### (1,7-Dihydro-6H-purine-9-β-D-ribofuranosyl-6-thiolato) (triethylphosphane) gold(I) (7)

108 mg (176 µmol, 88%) from 6-TNS (60.0 mg, 200 µmol, 1.00 eq.), Et_3_PAuCl (70.0 mg, 200 µmol, 1.00 eq.) and 200 µm KOH_(aq.)_ (1.00 mL, 200 µmol, 1.00 eq.) in EtOH (10 mL). m.p. 200 °C (decomp.); ^1^H NMR (500 MHz, DMSO-d_6_) *δ*_H_ = 8.55 (s, 1H, H^ar^), 8.40 (s, 1H, H^ar^), 5.93 (d, *J* = 5.7 Hz, 1H), 5.51 (s, 1H), 5.22 (d, *J* = 5.9 Hz, 1H), 5.22 (s, 2H), 4.58 (t, *J* = 5.4 Hz, 1H), 4.16 (dd, *J* = 4.9 Hz, 3.6 Hz, 1H), 3.95 (q, *J* = 3.8 Hz, 1H), 3.68 (dd, *J* = 12.0 Hz, 3.9 Hz, 1H), 3.58–3.49 (m, 1H), 1.96 (dq, *J* = 10.4, 7.6 Hz, 9H), 1.20 (dt, *J* = 18.6, 7.6 Hz, 6H). ^13^C NMR (126 MHz, DMSO-d_6_) *δ*_C_ = 151.1 (s), 148.0 (s), 141.7 (s), 133.4 (s), 87.8 (s), 85.6 (s), 73.5 (s), 70.3 (s), 17.3 (d, *J*_*CP*_ = 33.6 Hz), 8.86 (s). ^31^P NMR (202 MHz, DMSO-d_6_) *δ*_P_ = 37.1; HRMS (ESI): m/z calculated for C_16_H_27_AuN_4_O_4_PS + H^+^ [M + H^+^]: 599.11561. Found: 599.11330.

#### (2-Amino-1,7-dihydro-6H-purine-9-β-D-ribofuranosyl-6-thiolato) (triethylphosphane) gold(I) (8)

90.0 mg (150 µmol, 75%) from 6-TGS (56.7 mg, 200 µmol, 1.00 eq.), Et_3_PAuCl (70.0 mg, 200 µmol, 1.00 eq.) and 200 µm KOH_(aq.)_ (1.00 mL, 200 µmol, 10.0 eq.) in EtOH (10 mL). m.p. 170 °C (decomp.); ^1^H NMR (500 MHz, DMSO-d_6_) *δ*_H_ = 8.07 (s, 1H, H^ar^), 6.08 (s, 2H, NH_2_), 5.76 (d, *J* = 6.0 Hz, 1H), 5.20 (s, 3H), 5.22 (s, 2H), 4.48 (t, *J* = 5.5 Hz, 1H), 4.11 (dd, *J* = 5.0 Hz, 3.4 Hz, 1H), 3.65 (dd, *J* = 12.0 Hz, 3.9 Hz, 1H), 3.53 (dd, *J* = 12.0 Hz, 3.9 Hz, 1H), 1.94 (dq, *J* = 10.2, 7.6 Hz, 6H), 1.19 (dt, *J* = 18.5, 7.6 Hz, 9H); ^13^C NMR (126 MHz, DMSO-d_6_) *δ*_C_ = 171.9 (s), 159.4 (s), 150.4 (s), 137.5 (s), 126.6 (s), 86.7 (s), 85.2 (s), 73.3 (s), 70.4 (s), 61.4 (s), 17.3 (d, *J*_*CP*_ = 33.4 Hz), 8.82 (s); ^31^P NMR (202 MHz, DMSO-d_6_) *δ*_P_ = 36.8; HRMS (ESI): m/z calculated for C_16_H_27_AuN_5_O_4_PS + H^+^ [M + H^+^]: 614.12651. Found: 614.12425.

#### (1,7-Dihydro-6H-purine-2,6-bisthiolato) (triethylphosphane) gold(I) (9)

66.0 mg (81.3 µmol, 60%) from 2,6-dimercaptopurine (25.0 mg, 136 µmol, 1.00 eq.), Et_3_PAuCl (95.1 mg, 271 µmol, 2.00 eq.) and 200 µM KOH_(aq.)_ (1.36 mL, 271 µmol, 1.00 eq.) in EtOH (7 mL). m.p. 200 °C (decomp.); ^1^H NMR (500 MHz, DMSO-d_6_) *δ*_H_ = 12.46 (s, 1H, NH), 7.92 (s, 1H, H^ar^), 1.94 (dq, *J* = 15.2, 7.7 Hz, 12H), 1.18 (dt, *J* = 18.6, 7.7 Hz, 18H); ^13^C NMR (126 MHz, DMSO-d_6_) *δ*_C_ = 171.7 (s), 169.9 (s), 150.4 (s), 139.3 (s), 129.9 (s), 17.8 (d, *J*_*CP*_ = 33.4 Hz), 9.44 (d, *J*_*CP*_ = 37.7 Hz); ^31^P NMR (202 MHz, DMSO-d_6_): *δ*_P_ = 38.2, 36.6; HRMS (ESI): m/z calculated for C_17_H_32_Au_2_N_4_P_2_S_2_ + H^+^ [M + H^+^]: 813.09259. Found: 813.09361.

#### 6-Selenopurine

6-Chloropurine (250 mg, 1.62 mmol, 1.00 eq.) was dissolved in EtOH (5 mL), treated with selenourea (203 mg, 1.65 mmol, 1.02 eq.) and stirred at 108 °C for 1 h. The suspension was filtered and washed with H_2_O. The filter cake was dissolved in 2% NaHCO_3_(aq.) (25 mL) and the resulting mixture was stirred at 60 °C for 1.5 h. After cooling, the suspension was filtered and the filtrate was treated with acetic acid. Filtration, washing with water and drying in vacuo afforded 6-Selenopurine as orange solid (120 mg, 603 µmol, 37%). ^1^H NMR (500 MHz, DMSO-d_6_) *δ*_H_ = 14.26 (s, 1H, SeH), 13.68 (s, 1H, NH), 8.56 (s, 1H, H^ar^), 8.26 (s, 1H, H^ar^); ^13^C NMR (126 MHz, DMSO-d_6_) *δ*_C_ = 166.1 (s), 151.2 (s), 147.3 (s), 145.6 (s), 131.4 (s); ^77^Se NMR (95 MHz, DMSO-d_6_) *δ*_Se_ = 370.8. The NMR spectra match previously reported data [18].

#### (1,7-Dihydro-6H-purine-6-selenolato) (triethylphosphane) gold(I) (10)

72.0 mg (142 µmol, 94%) from 6-selenopurine (30.0 mg, 151 µmol, 1.00 eq.), Et_3_PAuCl (52.8 mg, 151 µmol, 1.00 eq.) and 200 µm KOH_(aq.)_ (753 µl, 285 µmol, 1.00 eq.) in EtOH (10 mL). m.p.: 140 °C (decomp.); ^1^H NMR (500 MHz, DMSO-d_6_) δ_H_ = 13.1 (s, 1H, NH), 8.39 (s, 1H, H^ar^), 8.33 (s, H^ar^), 1.94 (dq, *J* = 10.2 Hz, 7.6 Hz, 6H, CH_2_), 1.19 (dt, *J* = 18.6 Hz, 7.6 Hz, 9H, CH_3_); ^13^C NMR (126 MHz, DMSO-d_6_) *δ*_C_ = 163.4 (s), 152.1 (s), 151.1 (s), 145.6 (s), 135.5 (s), 17.9 (s), 17.7 (s), 9.28 (s); ^77^Se NMR (95 MHz, DMSO-d_6_) *δ*_Se_ = 166.6; ^31^P NMR (202 MHz, DMSO-d_6_) *δ*_P_ = 38.3; HRMS (ESI): m/z calculated for C_11_H_18_AuN_4_PSe + H^+^ [M + H^+^]: 515.01781. Found: 515.01613.

## Biological evaluation

### Stock solutions

The test compounds were dissolved in DMSO to a concentration of 10 mM and stored at − 23 °C. Prior to biological experiments, they were diluted to the desired concentration with sterile Millipore water.

### Cell culture conditions

518A2 human melanoma cells (Department of Radiotherapy & Radiobiology, University Hospital Vienna, Austria), HCT116^wt^ (DSMZ ACC-581) and its HCT116^p53−/−^ knockout mutant colon carcinoma cells, U87 glioblastoma cells (ATCC HTB-14), EA.hy926 somatic cell hybrid cells (ATCC CRL-2922), HeLa cervix carcinoma cells (DSMZ ACC-57), MCF-7 breast cancer cells (DSMZ ACC-115), HT-29 cisplatin resistant colon cancer cells (DSMZ ACC-299) and non-malignant human dermal fibroblasts HDFa (ATCC PCS-201-012) were cultured in Dulbecco’s modified Eagle medium (PAN biotech), supplemented with 10% (v/v) fetal bovine serum (Sigma-Aldrich) and 1% (v/v) ZellShield (Minerva Biolabs) at 37 °C under 95% humidity and 5% CO_2_. Unless noted otherwise, all bioassay steps including cells were conducted under these standard cell culture conditions. To maintain stable multidrug resistance in Kb-V1 cells, they were routinely treated with 340 nM vinblastine.

### Cytotoxicity and cellular uptake

#### 3-(4,5-dimethylthiazol-2-yl)-2,5-diphenyltetrazolium bromide (MTT) cell viability assay

All complexes stable in solution were investigated for their anti-proliferative effect on nine human cancer cell lines, using the MTT-based cell viability assay [19]. Cells were seeded in 96-well microtiter plates (Sarstedt) with a cell density of 0.05 × 10^6^ cells per ml and 100 µL per well and incubated for 24 h. A dilution series of test compounds was added to the wells, ranging in twelve steps from 100 µM to 5 nM. As a control, equal amounts of DMSO were added. Treated cells were further incubated for 72 h. Then, 12.5 µL per well of MTT solution (0.05% in phosphate-buffered saline (PBS)) was added, followed by another 2 h of incubation. The plates were centrifuged, the medium was discarded and 25 µL per well of SDS solution in DMSO (10% SDS, 0.6% AcOH in DMSO) was added to dissolve the formazan. The plates were incubated for a further hour. Absorbances at 570 nm and 630 nm were measured using a plate reader (Tecan). Background absorbance (630 nm) was subtracted from the formazan signal (570 nm). The resulting absorbance was directly proportional to the amount of viable cells. The control was normalized to 100% viable cells and viability of cells treated with test compounds was calculated accordingly. IC_50_ values were calculated based on a sigmoidal fit model using GraphPad Prism. Means and SD were calculated from at least four independent experiments.

### Cellular uptake measurement via ICP-MS

Cells were seeded at a density of 2 × 10^6^ cells per dish in cell culture dishes (Sarstedt) and grown overnight. The cells were then treated with the test compounds at a final concentration of 5 µM for 5 h. The cell monolayer was washed twice with PBS, the cells were harvested by trypsination, counted and pelleted (4 °C, 150 × g, 5 min). Cell pellets were solubilized with aqua regia (reflux, 20 min), and the gold content was determined by ICP-MS. Means and SD were calculated from at least two independent experiments.

### Lactate dehydrogenase (LDH) cytotoxicity assay

518A2 melanoma cells were seeded in 96-well flat bottom microtiter plates (Sarstedt) with 100 µL per well and a cell density of 0.05 × 10^6^ cells per mL. Wells containing medium alone were additionally set for background measurement. Cells were allowed to grow overnight followed by substance treatment with 11.1 µL of tenfold concentrated test compound dilutions and further incubated for 24 h. As a positive control, 10 µL per well of lysis solution (9% Triton-X 100 in Millipore H_2_O) was added and incubated for 45 min to maintain maximum LDH release, the same amount was added to maximum release background correction wells containing only medium. After centrifugation (4 °C, 150×*g*, 5 min), 50 µL of the supernatant of each well was transferred on a fresh microtiter plate followed by addition of 50 µL LDH assay buffer (223 mg of 2-*p*-iodophenyl-3-*p*-nitrophenyl-5-phenyltetrazolium chloride, 57 mg of *N*-methyl-phenazonium methyl sulfate, 575 mg of *N*-adenine dinucleotide, 3.2 g of lactic acid in 480 ml 200 mM Tris–Cl, pH 8.0) per well. The plate was incubated in the dark for 10–30 min at room temperature. Then 50 µL stop solution (1 M acetic acid) was added per well and the absorbance was measured at 490 nm. The mean value of background wells with medium alone was subtracted from the negative control and test wells. Additionally, the mean value of volume correction wells was subtracted from maximum LDH release wells. The percentage of LDH release was calculated, with the maximum LDH release set as 100% and the negative control as 0% release. Means and SD were calculated from at least three independent experiments [20].

### Apoptotic events

#### Caspase 3/7 activation

The assay was conducted, following manufacturer’s instructions using the Cell Meter Caspase 3/7 activity apoptosis assay kit (AAT Bioquest) [21]. Briefly, 518A2 melanoma cells were seeded in 96-well flat black microtiter plates with a density of 0.22 × 10^6^ cells per mL and 90 µL per well. After 24 h of incubation, 10 µL per well of tenfold diluted test compounds was added, and the cells were incubated for a further 6 h. Subsequently, 100 µL per well of Caspase 3/7 substrate working solution was added and incubated for 1 h in the dark at room temperature. The fluorescence was measured using a micro-plate reader (Tecan) at ex/em = 490/525 nm.

### ROS assay

To determine cellular ROS levels, 2,2′-bis(4-nitrophenyl)-5,5′-diphenyl-3,3′-(3,3′-dimethoxy-4,4′-diphenylene) di-tetrazolium chloride (NBT) was used. The assay is based on the reduction of a yellow NBT tetrazolium salt to a blue formazan dye. 518A2 melanoma cells were seeded in 96-well flat bottom microtiter plates (Sarstedt) with 100 µL per well and a cell density of 0.1 × 10^6^ cells per mL. The cells were allowed to grow overnight, followed by substance treatment with 11.1 µL of tenfold concentrated test compound dilutions and further incubated for 24 h. Then plates were centrifuged, the medium was discarded and 50 µL per well of NBT solution (0.1% in PBS) was added. The plates were further incubated for 4 h, centrifuged again and the medium was discarded. After that, 50 µL of 2M KOH solution and 65 µL DMSO had been added per well, incubation was continued for 30 min. The absorbances at 630 nm und 405 nm were measured, using a plate reader (Tecan). The background absorbance (405 nm) was subtracted from the formazan signal (630 nm). The resulting absorbance is directly proportional to the reactive oxygen species level. The control value was normalized to 100% ROS level, and accordingly, the viability of cells treated with test compounds was calculated. Means and SD were calculated from at least four independent experiments [22].

### Inhibition of TrxR activity

For the measurement of TrxR activity, the TrxR Colorimetric Assay Kit (Cayman Chemicals) [23] was used according to manufacturer’s instructions. 1 × 10^8^ 518A2 melanoma cells were harvested using a cell scraper, homogenized in 5 mL of cold lysis buffer (50 mM K_3_PO_4_, 1 mM EDTA, pH 7.4) on ice and centrifuged for 15 min (4 °C, 10,000×*g*). The protein concentration of the supernatant was determined via Nanodrop. Then, 10 µL Protease Inhibitor Cocktail Plus (Carl Roth) was added to 1 mL of the cell lysate, which was either used for the assay right away or stored at − 80 °C. Prior to use, the assay buffer was warmed to room temperature and the cell lysate, NADPH (Nicotinamide adenine dinucleotide phosphate), ATM (aurothiomalate) and rat liver TrxR enzyme were thawed and kept on ice After determination of the amount of cell lysate for optimum TrxR activity, alle components were pipetted into the wells of a clear 96-well plate and the enzymatic reactions were initiated by addition of NADPH and 5,5′-dithiobis-(2-nitrobenzoic acid (DTNB). Then, the absorbance at 405 nm was measured once every minute using a plate reader (Tecan) at least ten time points. The TrxR activity was measured in the presence and absence of ATM. The difference between the two results renders the DTNB reduction due to TrxR activity. By plotting the average absorbance values as a function of time, the slope of the linear portion of the curve was obtained, and the change of absorbance (ΔA405) per minute could be determined. The values were corrected for unspecific DTNB reduction and the TrxR activity was calculated using the following formula: TrxR activity [µmol/min/mL] = [corrected ΔA/min (sample)/7.92 mM^−1^] × [0.2 mL/0.02 mL] × sample dilution. The assay was conducted at 22 °C. All experiments were performed in triplicate and the solvent-treated negative controls were set to 100%.

### Influence on the cell cycle

The complexes were investigated for their effect on the cell cycle [24]. Cells were seeded in 6-well plates (Sarstedt) with 3 mL per well and a cell density of 0.05 × 10^6^ cells per mL and incubated for 24 h. Cells were treated with concentrations of compounds, corresponding their IC_50_ and further incubated for 24 h. The supernatant of each sample was transferred into a separate tube on ice, the cell monolayer was rinsed once with PBS and the cells were harvested using trypsin, and also transferred into the tube. The cells were pelleted (4 °C, 150 × g, 5 min), re-suspended in 1 mL ice cold EtOH (70%) and stored for at least 1 h at 4 °C. Prior to propidium iodide (PI) staining, the cells were centrifuged (150×*g*, 5 min), the supernatant was discarded and the cells were layered with 1 mL PBS for 5 min. The cells were centrifuged again and the pellet was re-suspended in 200 µL of PI staining solution (50 µg/mL PI, 1% sodium citrate in PBS) containing 1 µL RNase (10 mg/mL stock solution) and incubated in dark for 30 min at 37 °C. The cell cycle distribution was assessed by flow cytometry (Beckmann Coulter). Means and SD were calculated from at least three independent experiments.

### Tubulin polymerisation assay

First, 2 × polymerization buffer was prepared, which consisted of 385 µL Brinkley renaturing buffer 80 (BRB 80), 100 µL glycerol, and 15 µL GTP (100 mM). 50 µL of freshly prepared 2 × polymerization buffer was added to µClear black well plates. Subsequently, 11.1 µL each of a tenfold substance pre-dilution in BRB80 was added. In addition, a combretastatin A-4 positive control was prepared analogously. After rapid addition of 50 µL of tubulin per well, the measurement was started on the plate reader at 340 nm. The plate reader measured at 20 s intervals for at least 120 min until the measurement plateau was reached. For evaluation, OD 340 nm was plotted versus time [25].

### Inhibition of wound healing (scratch migration assay)

Cells of melanoma cell line 518A2 were seeded in a volume of 500 µL and at a concentration of 0.1 × 10^6^ cells/mL in 24-well plates in triplicate. The plate was incubated (37 °C, 95% humidity, 5% CO_2_) for two days until a confluent monolayer was formed.  A 10 µL pipette tip was used to create a wound in the cell lawn, the wound was rinsed with PBS and the medium was changed to a FBS-reduced one. Pictures were taken at first time point (0 h) right before the addition of substance at 100-fold pre-dilution. The plate was then incubated again in the incubator and photos were taken at several more time points (6, 12, and 24 h) [26,26,26].

**Fig. 1:**
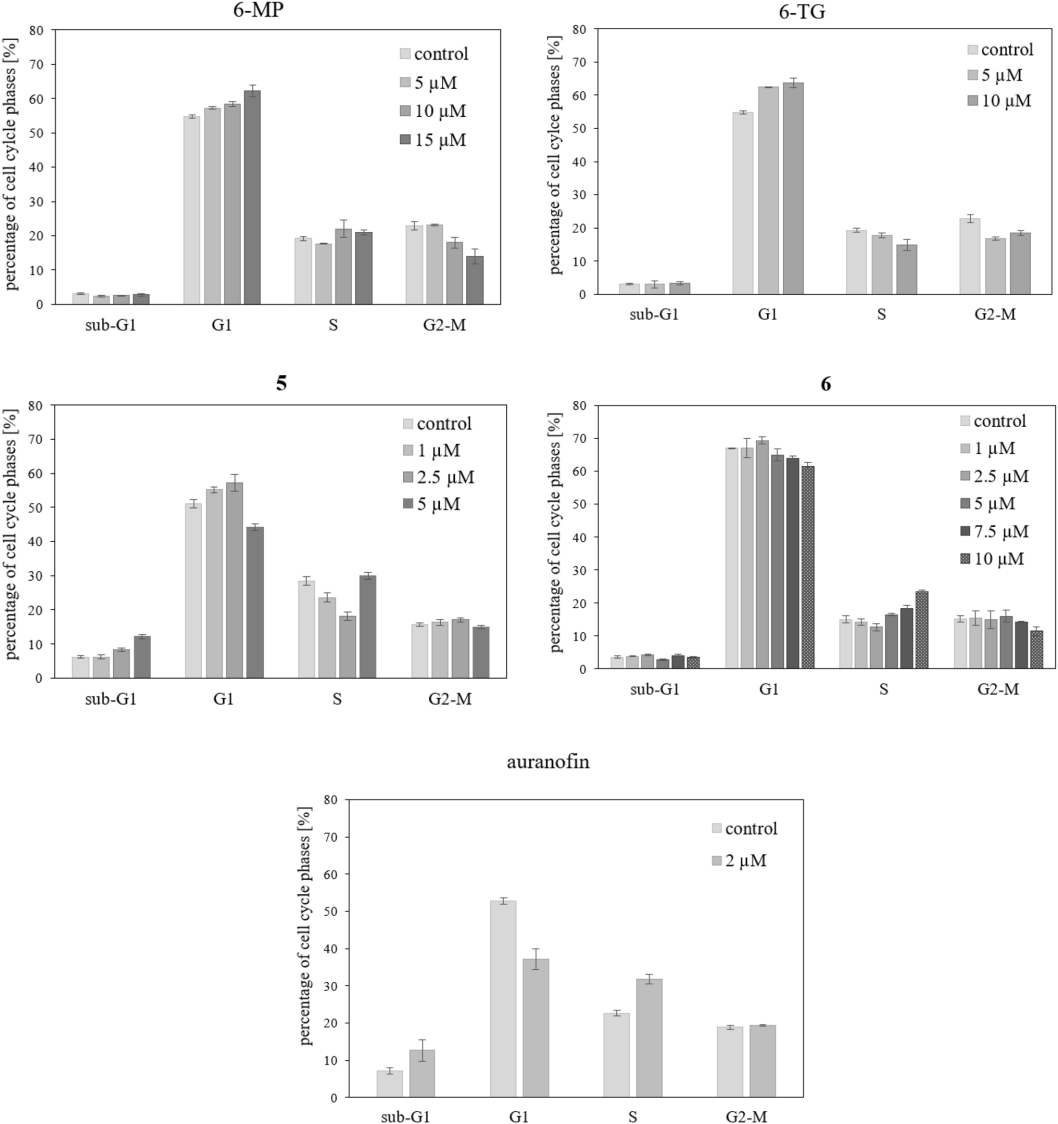
Effect on the cell cycle of 518A2 melanoma cells after 24 h incubation with different concentrations of 6-MP, 6-TG, and auranofin, as well as complexes **5** and **6**. Percentage of 518A2 melanoma cells in G_1_, S and G_2_/M cell cycle phases and the proportion of potentially apoptotic cells (sub-G1) as determined by flow cytometry. Values are means ± SD of at least three independent experiments

### DNA interaction

#### Ethidium bromide saturation assay (EtdBr assay)

The interaction of the complexes with linear DNA was assessed using the EtdBr assay. Ethidium bromide intercalates into DNA enhancing its fluorescence, while intercalation is hindered by alterations of the DNA structure e.g., by small molecules interfering with DNA [17]. In wells of a 96-well black flat bottom microtiter plates a solution of 1 µg linear salmon sperm DNA (ThermoFisher) in TE buffer (10 mM Tris–Cl, 1 mM EDTA, pH 8.0) was treated with concentration series (final concentrations; 25 µM, 50 µM, 75 µM and 100 µM) of the compounds, a corresponding amount of DMSO was used as a control (0 µM). After an incubation period of 2 h at 37 °C, 100 µL per well of ethidium bromide solution (10 µg/mL in TE buffer) was added and the plate was incubated for 5 min in the dark. Background samples were prepared analogously but without DNA addition. EtdBr–DNA adduct fluorescence was monitored at an excitation wavelength of 535 nm and an emission wavelength of 595 nm. After background subtraction, changes in fluorescence intensity were calculated in relation to control (set to 100%). Means and SD were calculated from at least three independent experiments.

### Electrophoretic mobility shift assay (EMSA)

To distinguish whether the DNA interaction is of an electrostatic or covalent nature, a second DNA interaction assay was conducted. 1.5 µg of circular plasmid pBR322 DNA were incubated in TE buffer (10 mM Tris–Cl, 1 mM EDTA, pH 8.0) with different concentrations of test compounds (final concentrations; 5 µM, 10 µM, 25 µM and 50 µM), whereas a corresponding volume of DMSO was added to the control (0 µM) to a final volume of 20 µL of each sample. Samples ware incubated for 16 h at 37 °C and subjected to 1% agarose gel electrophoresis in 0.5 × TBE buffer (45 mM Tris–Cl, 45 mM boric acid, 1.25 mM EDTA, pH 8.0) for 4 h at 66 V. Afterward, the agarose gel was stained for 20 min with EtdBr (10 µg/mL in 0.5 TBE buffer) solution and the result documented using UV excitation.

### Comet assay

518A2 melanoma cells were seeded at a concentration of 0.03 × 10^6^ cells/mL and a volume of 2 mL seeded in 6-well plates and incubated overnight (37 °C, 95% humidity, 5% CO_2_). In addition, slides were coated with 1.5% agarose and allowed to harden before being stored in a moist place until the next day. On the second day, a 100-fold substance pre-dilution was added to the cells followed by a further incubation of 3–5 h. The selected concentration range was based on Mendonça et al. [27]. Subsequently, the cells were harvested with trypsin and the cell number was adjusted to 0.02 × 10^6^ cells/mL. 0.5% agarose solution was prepared and tempered to a maximum of 50 °C. After mixing 0.4 mL of cell suspension with 1.2 mL of tempered agarose, 200 µL was pipetted onto each of the previously coated slides. After these had hardened, a one-hour incubation followed at 4 °C in lysis buffer. The samples were then electrophoresed at 11 V for 20 min and then incubated for 15 min in neutralization buffer. Finally, the DNA was stained with 30 µL each of 1% ethidium bromide for 30 min, before the slides were washed with PBS for subsequent documentation under a fluorescent microscope with UV excitation (Zeiss Axiovert 135, Ex/Em 300 nm/605 nm).

### Tube formation assay

EA.hy926 cells were cultured overnight in endothelial medium (37 °C, 95% humidity, 5% CO_2_) instead of DMEM. In addition, Matrigel was thawed on ice in the refrigerator. Pipette tips and 96-well plates were stored at − 20 °C overnight. The next day, 30 µL of Matrigel was added to each well bubble-free and the well plate was incubated for approximately 45 min (37 °C, 95% humidity, 5% CO_2_). Subsequently, 100 µL of cell suspension was added to each well at a concentration of 0.4–0.5 × 10^6^ cells/mL onto the Matrigel-treated wells and the substances were added in tenfold pre-dilution. This was followed by incubation for approximately 6 h, until tubes formed in the negative control with DMSO. At this point, documentation under the microscope took place [28].

## Results

### Synthesis and characterization

**Scheme 1:**
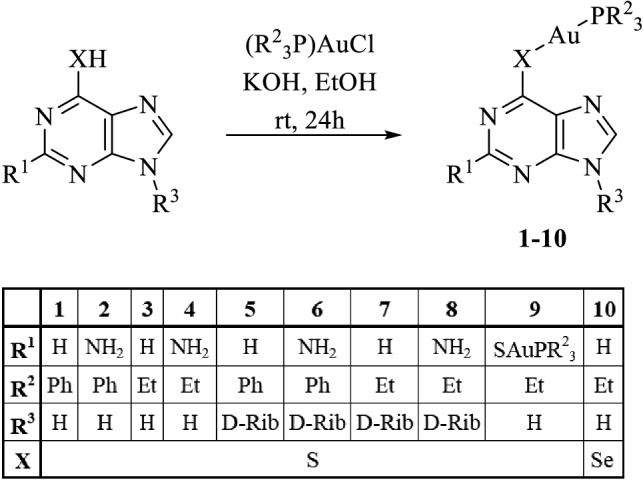
Synthesis of complexes **1**–**10**

Chlorotriorganophosphane gold(I) precursors were synthesized according to well established literature protocols [29]. The purine-6-thiolato gold(I) complexes **1**–**9** were synthesized analogously to a procedure by Tiekink et al. (Scheme 1) [30]. No protection of the hydroxy groups of the riboside was necessary due to the higher pKa values of the thiols and their higher aurophilicity, compared to alcohols. The 9*H*-purine-6-thiolato gold(I) complexes **1**–**4** and **9** were obtained with an average yield of 60%, their nucleoside analogs **5**–**8** in yields around 45%. Complexation of 2,6-dimercaptopurine with two equivalents of (PEt_3_)AuCl and base afforded the dinuclear complex **9** displaying two peaks in the ^31^P NMR spectrum. Purine-6-selenolato complex **10** was prepared by reacting 6-chloropurine with selenourea to afford 6-selenopurine which in turn was complexed exactly like the mercaptopurines and nucleosides. All complexes, designated for in vitro bio tests, were characterized by ^1^H, ^13^C, ^31^P and, if applicable, ^77^Se NMR spectroscopy, as well as ESI mass spectrometry.

None of the complexes showed a change of their signals in ^1^H NMR spectra (*cf.* Supporting Information) over the period of at least three days when dissolved in DMSO-d_6_ + 5% D_2_O, i.e., under testing conditions and can thus be considered stable. The disappearance of the amine and hydroxy protons immediately after the addition of water can be explained by the isotope exchange with the D_2_O present in large excess. ^31^P NMR spectra of 9*H*-purine complex **4** and nucleoside complex **7**, measured with additional D_2_O, also did not change noticeably in the course of three days, confirming the stability of the coordination of phosphorus to the gold atom. In a further corroboration of the complex stability, the similarity of the chalcogens sulfur and selenium was exploited. The selenolato complex **10** was synthesized and examined for its stability under identical conditions. Since ^77^Se is a 1/2 spin nucleus with roughly 8% natural abundance, its compounds can be investigated directly. Depending on whether a break in the Se–Au or the Au–PR_3_ bond would occur, the shift would change by several hundred ppm and serve as unequivocal evidence. Here, too, no change in the shift of the ^77^Se NMR signal was observed. In addition, we investigated the stability of complexes **1** and **5** as representatives of the series of purines and purine nucleosides, respectively, in cell culture medium by UV/Vis spectroscopy. Their signals did not change at all in the short period of 0–30 min and also in the longer period of 3 days only slightly by a decrease in their signals intensity. The complexes can thus be considered stable under the test conditions (*cf.* Supporting Information).

## Biological evaluation

### Cytotoxicity against cancer cells

All complexes **1–9** were evaluated in MTT-assays for their cytotoxicity against cancer cell lines of different tumor entities, as well as a non-malignant fibroblast control cell line (Table 1). In contrast to studies by Tiekink et al., no general improvement in the cytotoxicities of the thio-purines was observed upon complexation with a tri-alkyl-phosphane gold(I) cation. For most cell lines, the IC_50_ values of complexes **1-3** and the corresponding thiopurines were approximately the same. The complexes were sometimes more and sometimes less cytotoxic than the free bases within the range of the standard deviation. Complex **4**, however, was distinctly less active compared to its analogs. The new purine nucleoside complexes, however, showed higher cytotoxicities overall. Complex **5** performed best with IC_50_ values ranging between three-digit nano-molar and single-digit micromolar. Healthy human dermal fibroblasts (HDFa) were far less affected. Complex **5** surpassed the cytotoxic effects of the positive control 6-MP on all listed cancer cell lines. It was also as active on average as the positive controls 6-TG and auranofin. Only 6-MMR displayed a stronger activity than complex **5**, yet was also more cytotoxic against HDFa. Considering all complexes **1–8**, some SAR became apparent. Derivatization of the purine with d-ribose at N9 resulted in stronger cytotoxic effects, at least for the couples **1**/**5**, **3**/**7** and **4**/**8**. The introduction of a R^1^ = NH_2_ group at C2 also had an effect on the cytotoxicity. While complex **1** was less cytotoxic against all tested cell lines than its 2-amino derivative **2**, the 2-H complexes were more active against the tumor cell line panel than their 2-amino congeners in the couples **3**/**4**, **5**/**6**, and **7**/**8**. As to the influence of the phosphane residues R^2^, the tri-phenyl-phosphane(9*H*-purine-6-thiolato) complexes **1** and **2** were distinctly more cytotoxic against the tumor cells when compared with their triethylphosphane(9*H*-purine-6-thiolato) analogs **3** and **4**, respectively. No such difference in activities was observed for the corresponding nucleoside complex couples **5**/**7** and **6**/**8**.

**Table 1:** Inhibitory concentrations IC_50_ [µM] of 6-MP, 6-TG, 6-MMR, auranofin and complexes **1**–**9** when applied to EA.hy926 endothelial hybrid cells and cells of human HCT-116, HCT-116^p53−/−^ (p53 knockout mutant) and HT-29 colon carcinomas, HeLa and mdr KB-V1^Vbl^ cervix carcinoma (treated with and without 1 µM verapamil), MCF-7 and mdr MCF-7^Topo^ mamma carcinoma, U-87 glioblastoma, 518A2 melanoma, and human adult dermal fibroblasts HDFa

	EA.hy926	HCT-116^wt^	HCT-116^p53−/−^	HeLa	HT-29	KB-V1^Vbl^
6-MP	0.92 ± 0.04	5.3 ± 0.28	0.58 ± 0.05	1.4 ± 0.10	1.4 ± 0.06	1.6 ± 0.10
6-TG	0.25 ± 0.02	0.97 ± 0.10	1.0 ± 0.02	0.19 ± 0.01	1.2 ± 0.08	0.50 ± 0.05
Auranofin	0.82 ± 0.04	11.9 ± 0.4*	5.0 ± 0.2*	2.6 ± 0.4*	2.0 ± 0.18	1.9 ± 0.07
6-MMR	0.11 ± 0.001	0.10 ± 0.008	0.050 ± 0.001	0.0056 ± 0.0003	–	–
1	2.9 ± 0.70	4.9 ± 1.0	1.7 ± 0.30	2.6 ± 0.04	8.0 ± 0.73	1.9 ± 0.1
2	0.50 ± 0.10	0.80 ± 0.01	0.58 ± 0.01	0.60 ± 0.05	4.4 ± 0.32	0.30 ± 0.07
3	1.5 ± 0.11	6.3 ± 0.30	3.6 ± 0.80	6.8 ± 0.62	6.4 ± 1.4	2.5 ± 0.32
4	1.1 ± 0.08	25 ± 3.1	11 ± 0.50	15 ± 4.1	5.8 ± 1.7	12 ± 1.0
5	0.34 ± 0.03	1.7 ± 0.20	0.30 ± 0.07	0.19 ± 0.01	1.2 ± 0.09	0.50 ± 0.05
6	3.8 ± 0.13	5.5 ± 0.20	1.4 ± 0.30	2.4 ± 0.14	3.1 ± 0.19	0.91 ± 0.09
7	0.43 ± 0.02	1.6 ± 0.20	0.50 ± 0.03	0.51 ± 0.05	2.2 ± 0.17	0.40 ± 0.04
8	1.7 ± 0.14	5.5 ± 0.30	1.8 ± 0.06	2.4 ± 0.21	4.5 ± 0.34	0.70 ± 0.07
9	–	11.2 ± 0.78	3.7 ± 0.35	17.6 ± 0.80	11.1 ± 0.32	–

KB-V1^Vp^	MCF7	MCF7^Topo^	U-87	518A2	HDFa
1.4 ± 0.14	3.7 ± 0.26	6.4 ± 0.46	1.4 ± 0.13	11 ± 1.1	> 50
0.42 ± 0.04	0.98 ± 0.08	0.90 ± 0.09	1.7 ± 0.08	5.2 ± 0.45	> 50
2.1 ± 0.19	2.2 ± 0.22	4.7 ± 0.43	2.4 ± 0.24	1.8 ± 0.03*	14 ± 1.0*
–	0.090 ± 0.003	–	–	1.4 ± 0.12	5.8 ± 0.46
1.7 ± 0.77	13.0 ± 1.12	6.6 ± 0.65	0.91 ± 0.09	8.0 ± 0.71	–
0.54 ± 0.02	2.2 ± 0.13	3.7 ± 0.37	1.0 ± 0.1	4.4 ± 0.33	–
1.1 ± 0.11	9.4 ± 0.53	10 ± 0.26	6.9 ± 0.47	8.4 ± 0.62	–
10.4 ± 0.95	21 ± 3.40	4.9 ± 0.43	7.0 ± 0.66	16 ± 0.91	–
0.50 ± 0.03	2.4 ± 0.22	1.7 ± 0.14	1.7 ± 0.15	2.6 ± 0.25	36 ± 1.70
1.5 ± 0.11	5.8 ± 0.57	3.2 ± 0.29	1.2 ± 0.12	3.5 ± 0.26	36 ± 3.00
0.62 ± 0.04	2.1 ± 0.20	1.2 ± 0.11	1.1 ± 0.11	3.0 ± 0.21	7.0 ± 0.62
1.8 ± 0.17	10 ± 0.83	3.6 ± 0.34	1.0 ± 0.03	3.4 ± 0.33	7.0 ± 0.70
–	–	–	–	13.4 ± 1.20	–

Values are derived from dose–response curves obtained by measuring the percentage of vital cells of treated wells relative to untreated controls after 72 h of incubation using MTT-assays. Values are means ± SD of at least four independent experiments. Values marked with * were taken from Bär et al. [33]

Tests on tumor cell line mutants in comparison with their wild type also led to telling results. HCT-116 colon carcinoma cells lacking functional p53 protein, which is also frequently mutated in tumors, were more sensitive to all tested complexes than HCT116^wt^ cells. Moreover, complexes **1** and **4–8** showed an increased cytotoxicity against MCF7 breast cancer cells, previously rendered multi-drug resistant by repeated treatment with topotecan, when compared to untreated, sensitive MCF7 cells. In addition, most tested complexes were of similar cytotoxicity against KB-V1 cervix carcinoma cells in the absence and presence of the Pgp-substrate verapamil, clinically used to re-sensitize resistant tumors [31]. This suggests that these complexes are not substrates of ABC-transporter-type efflux pumps [32, 33].

### Cellular uptake

For an assessment of the uptake of the test compounds **1–8** into 518A2 melanoma cells, the gold content of lysed treated cells was measured by ICP-MS (*cf.* Supporting Information). Complex **6**, the only one that features a 2-NH_2_ group, a 9-d-ribosyl residue and a tri-phenyl-phosphane ligand, was taken up conspicuously well to a concentration of 164 µg/10^6^ cells. Given its moderate IC_50_ value of ca 3.5 µM for this cell line in the MTT-assays, when compared to the other test complexes and auranofin the intrinsic cytotoxicity of complex **6** is rather low. Complex **5** and auranofin were taken up to roughly the same extent which nicely matches their similar cytotoxicities against 518A2 melanoma cells.

### Influence on the cell cycle

**Fig. 2:**
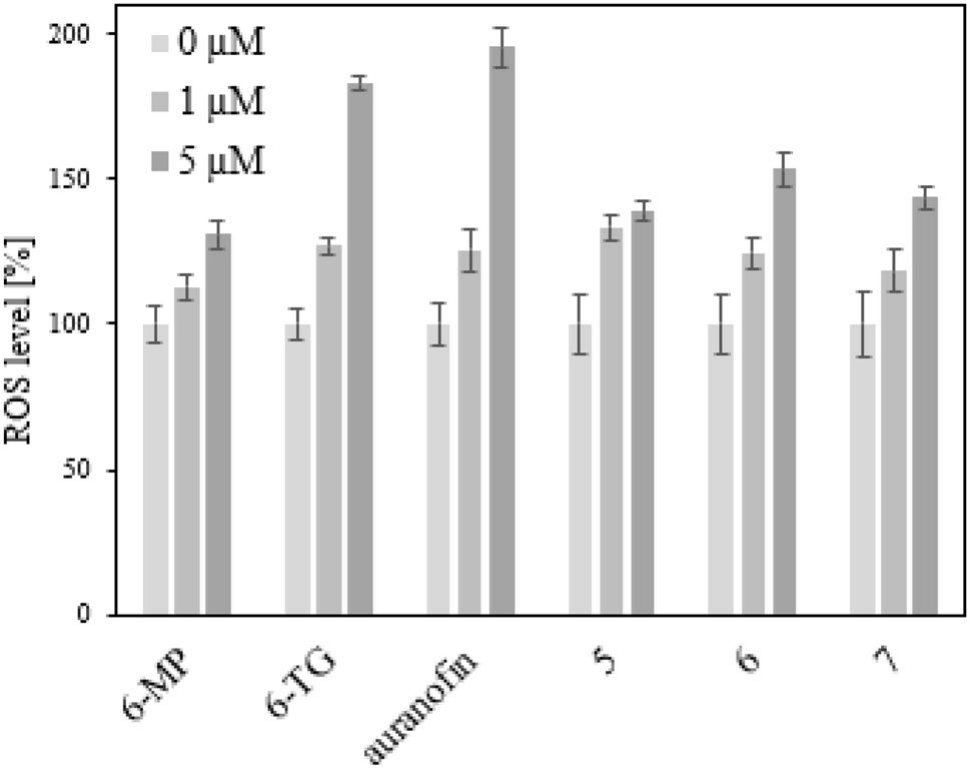
Relative increase of the intracellular concentration of reactive oxygen species in 518A2 melanoma cells after treatment with solvent (0 µM) or 6-MP, 6-TG, auranofin or complexes **5**–**7** (1 µM and 5 µM) for 24 h. All experiments were performed in sextuplicate. ROS levels were calculated as mean ± SD with respect to untreated control set to 100%

While 518A2 melanoma cells treated with the solvent DMSO showed the normal distribution over all cell cycle phases, cells treated with the test complexes, 6-MP, or 6-TG accumulated in G1-phase. In contrast, cells treated with IC_50_ concentrations of auranofin were arrested mainly in S-phase (Fig. 1, 2). The incorporation of thiopurines into DNA leads to DNA damage, which the cell tries to repair in G1-phase [34]. Auranofin inhibits TrxR which is important for some cellular processes during S-phase of the cell cycle [35]. Complex **5** displayed both a G_1_-phase arrest and an S-phase arrest when applied at approximately twice its IC_50_. Complex **6** exhibited a similar concentration-dependent bimodal effect. A possible explanation of this effect could be that at lower concentrations the entire compound can be accommodated in the DNA, eliciting a thiopurine-like G_1_-phase arrest. With concentration increasing, the cytotoxic effect typical of auranofin comes to the fore, ultimately leading to an S-phase arrest.

### Influence on the intracellular ROS concentration

The influence of the complexes **5**–**7**, 6-MP, 6-TG and auranofin on the concentration of ROS in 518A2 melanoma cells was assessed by NBT-assay. All compounds led to a similar increase of ROS levels. TrxR inhibitor auranofin, when applied at a concentration of 5 µM gave rise to the highest ROS levels, presumably due to an accumulation of oxidized thioredoxin [4].(Fig. 2).

### Influence on TrxR

**Fig. 3:**
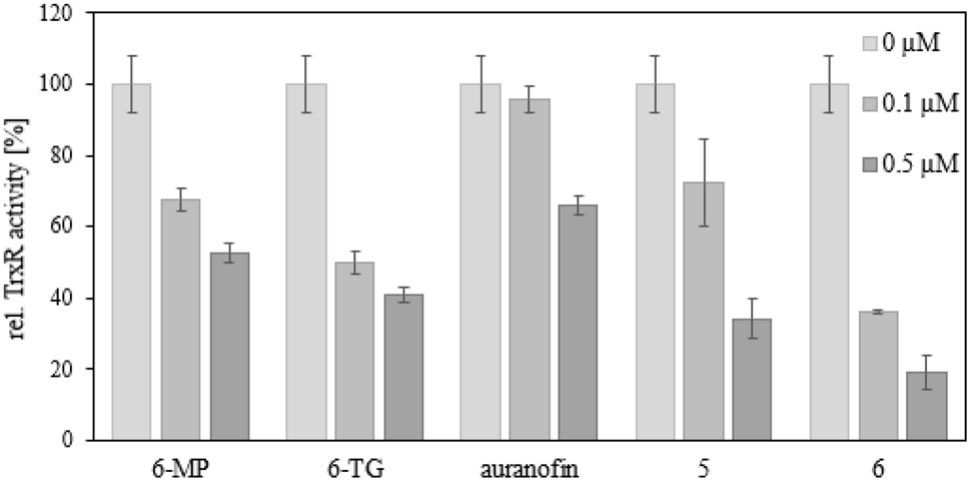
Concentration-dependent inhibition of TrxR activity in lysates of 518A2 melanoma cells by 6-MP, 6-TG, auranofin and complexes **5** and **6**. TrxR-independent substrate reduction was accounted for by experiments in the presence and absence of the specific TrxR inhibitor aurothiomalate. All values are means ± SD of at least four independent experiments with negative controls (0 µM) set to 100%

Like auranofin, the thiopurines 6-MP and 6-TG also inhibited TrxR. The enzyme is indirectly involved in DNA synthesis, by reducing thioredoxin, which gets oxidized by ribonucleotide reductase [36]. Combining the structural motifs of auranofin and thiopurines led to compounds exhibiting a much stronger inhibition of TrxR. There was no correlation between TrxR activity and ROS levels, which indicates that auranofin is involved in other redox pathways such as the glutathione system [37]. The complexes **5** and **6** inhibited TrxR even more strongly than 6-MP, 6-TG and auranofin (Fig. 3).

### Induction of apoptosis and necrosis

Auranofin inhibits the ubiquitin proteasome system, associated with induction of apoptosis [35, 38]. Both thiopurines 6-MP and 6-TG induce programmed cell death via mismatch repair mechanisms [39]. To detect any potential induction of apoptosis by the test complexes, a caspase-based fluorescence assay was used [35, 38]. Both thiopurines 6-MP and 6-TG induce programmed cell death via mismatch repair mechanisms [39]. To detect any potential induction of apoptosis by the test complexes, a caspase-based fluorescence assay was used. Executioner caspases **3** and **7** are activated in the course of the caspase cascade. Their activation can thus be taken as a measure of apoptosis induction. Both thiopurines and auranofin, but also complexes **5–7** led to increased caspase activity levels, most prominently so the complexes **6** and **7** which even exceeded the effects of the three controls.

To prove that apoptotic rather than necrotic cell death was induced, a lactate dehydrogenase (LDH)-based assay was employed. Potential drugs are supposed not to cause necrosis which leads to inflammation of healthy neighboring tissue. The release of the intracellular enzyme LDH upon drug treatment allows to gage the risk of it inducing necrosis. In comparison to a positive control, which shows the maximum LDH release, no enzyme could be detected upon treatment of 518A2 melanoma cells with 6-MP, 6-TG, auranofin or the complexes **5** and **6**. This confirms that the latter induce exclusively apoptosis and no necrosis (*cf.* Supporting Information).

### Influence on tubulin polymerization

Since polymerization of tubulin plays an essential role in mitosis, its inhibition is an ideal strategy for cancer therapy. Among the inhibitors of tubulin polymerization there are already many pyrimidine-derived agents [40]. We found that 6-MP and even more so 6-TG inhibited tubulin polymerization in vitro by turbidity measurements at 340 nm using a plate reader. Auranofin also turned out to be a microtubule-destabilizing agent, probably acting by binding to the sulfhydryl groups of tubulin, which were reported to serve as targets of other polymerization inhibitors [41, 42]. Complexes **5** and **6** led to a merely slight inhibition of tubulin polymerization in vitro (*cf.* Supporting Information).

### Inhibition of cancer cell migration

**Fig. 4:**
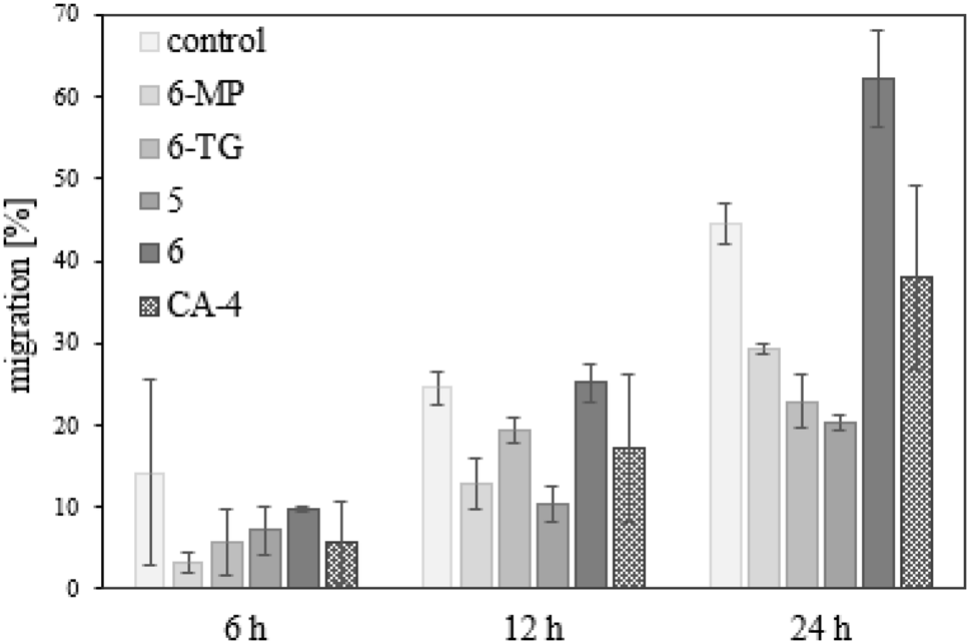
Effects on cell migration of DMSO (control), 6-MP, 6-TG, complexes **5**–**6** and CA-4 as positive control. 518A2 melanoma cells were treated with the respective IC_50_ concentrations and their measured after 0, 6, 12 and 24 h. All experiments were performed as triplicates. Values are displayed as means ± SD. Migration progress was set to 0% at 0 h

The ability of cancer cells to migrate has been shown to be a prerequisite for the tissue invasion and metastasis. A variety of cell adhesion molecules contribute to tumor suppression and thus reduce cell migration [43]. The scratch migration assay is a simple method for identifying potential inhibitors of cell motility. “Wounds” were created in the lawn of 518A2 melanoma cells and treated with the test compounds. The closure of the scratches was followed photographically and subsequently measured at three different points [44–47]. While complex **5** retarded the closure of the scratch "wound" relative to an untreated control, complex **6** even accelerated it. The only difference structure-wise between these two is the 2-NH_2_ group of **6** (Fig. 4).

### Influence on DNA

**Fig. 5:**
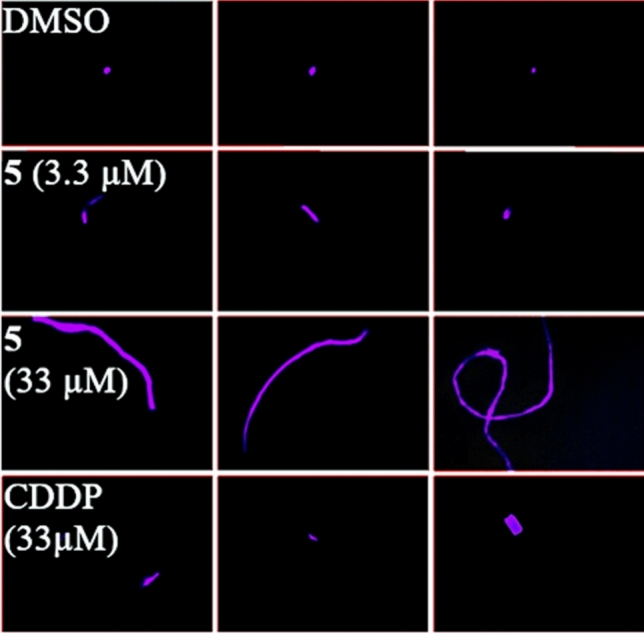
DNA damage upon treatment with complex **5** (3.3 µM and 33 µM) and CDDP (33 µM) as positive control, documented with comet assays. Treatment with DMSO as a negative control. Photos are representative of the respective compound and concentration. The images were taken at 100-fold magnification and were processed for better recognizability in terms of brightness and contrast. DNA damage is visualized by migration of DNA fragments out of the nucleus, which is reminiscent of a comet tail

As the mechanism of action of thiopurines involves interaction with and potential damage of DNA, we carried out electrophoretic mobility shift assays (EMSA) with circular pBR322 plasmid DNA, and ethidium bromide saturation assays with linear salmon sperm DNA, both of which failed to reveal any positive effects for the so far best performing complex **5**. Presumably, it does not interact with DNA directly, but is metabolized in cells similarly to 6-MP and 6-TG, which are erroneously incorporated into DNA eventually leading to apoptosis induction [48]. This assumption was confirmed by comet assays, where treatment of single 518A2 melanoma cells with complex **5** led to DNA damage, which is reflected in the migration of stained DNA fragments during gel electrophoresis (Fig. 5).

### Anti-angiogenic effects

**Fig. 6:**
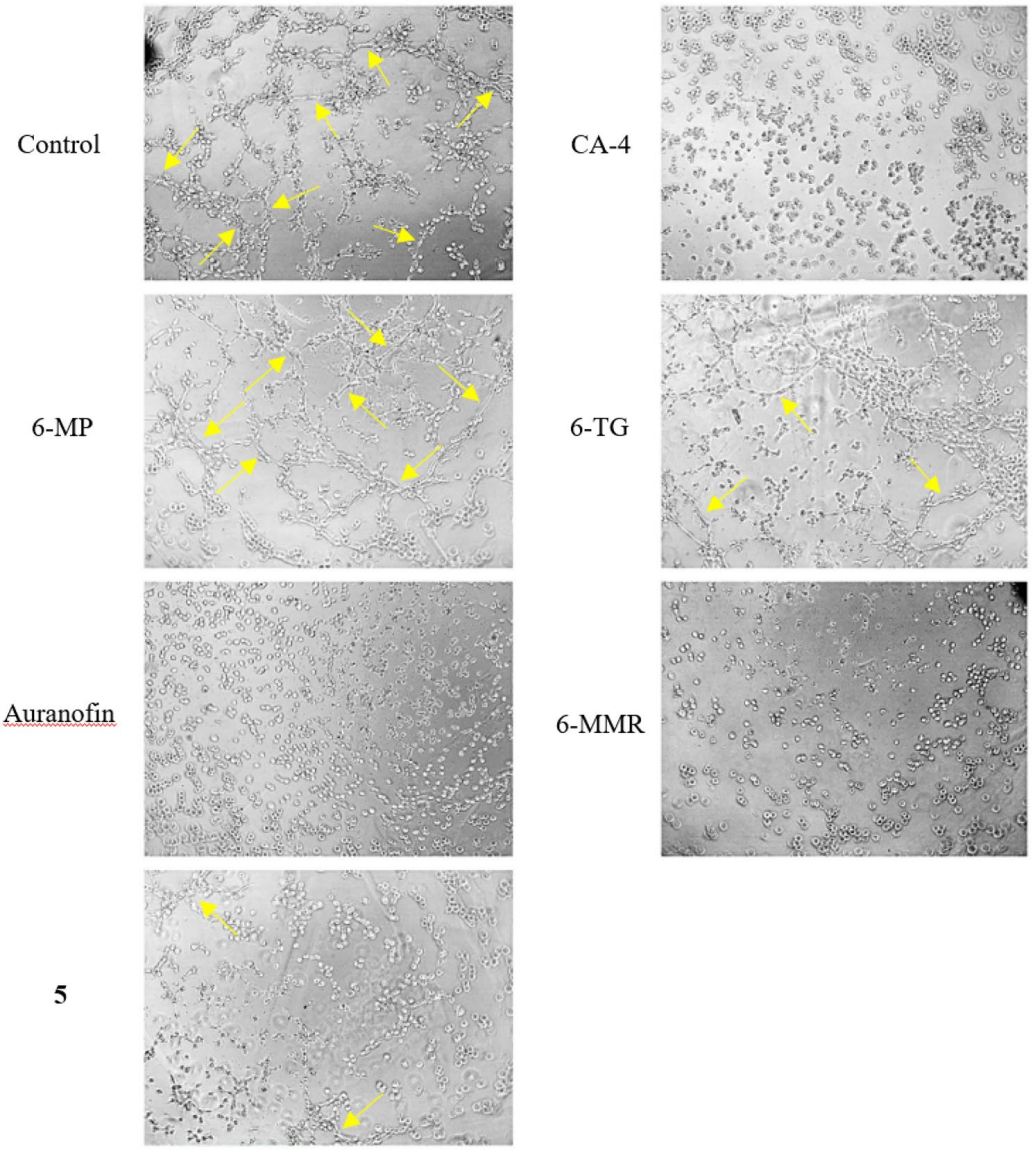
Tube formation assays: Inhibition of tube formation of Ea.hy926 cells through 6-MP, 6-TG, auranofin, 6-MMR and complex **5**, as well as DMSO (neg. control) and CA-4 (pos. control). The formation of tubes, some of which are indicated by yellow arrows, mimics vessel formation during angiogenesis. The lack of it is an indication for anti-angiogenic effects of test compounds. Final concentrations were 5 µM, except for CA-4 (0.01 µM). Photos are representative of the respective compound. The images were taken at tenfold magnification and were processed for better recognizability in terms of brightness and contrast

Like auranofin, 6-MMR also has an anti-angiogenic effect [17, 35]. Inhibition of angiogenesis is considered a promising strategy in cancer therapy since the growth of tumors requires the development of a vascular network [49]. Auranofin inhibits angiogenesis, at least in zebrafish models, via interference with the VEGF signaling pathway [50]. 6-MMR inhibits the early and late phases of the angiogenesis process. The early phase of angiogenesis is associated with cellular capabilities, such as cell proliferation, cell mobility and sprouting. The late phase comprises the formation of capillary structures, which can be ascertained by the tube formation assay [17]. We could confirm the known inhibitory effects of auranofin and 6-MMR on the formation of blood vessel-like tubes by Ea.hy926 cells. Complete inhibition of tube formation, such as by the positive control CA-4, is evident by the presence exclusively of individual cells, whereas the negative control DMSO allowed the formation of the tube-like structures, mimicking the angiogenesis process. This simple test indicates anti-angiogenic properties of the compounds. 6-TG had a stronger such effect when compared to 6-MP. Interestingly, complex **5** also had such an inhibitory effect, comparable in strength to that of 6-TG (Fig. 6).

## Conclusion

Four tri-organyl-phosphane(9*H*-purine-6-thiolato) gold(I) complexes with or without 2-NH_2_ residues, as well as four new analogous complexes with 6-TGS and 6-TNS ligands, and a dinuclear 2,6-dimercaptopurine gold(I) complex were synthesized and characterized. The complexation worked for purines as well as for nucleosides without any change of the reaction conditions. All complexes were stable under test conditions (5% D_2_O in DMSO-d_6_) over a period of at least three days. This assumption was confirmed by monitoring the stability under these conditions of an analogous purine-6-selenato gold(I) complex via ^77^Se-NMR spectroscopy.

The gold complexes **1**–**8** showed cytotoxic effects with IC_50_ values in the low micromolar to three-digit nano-molar range against a panel of seven cancer cell lines, with complex **5** performing best on average (0.19–2.5 µM). A few tentative SAR emerged from the MTT assays. In most cases, attachment of a 9-d-ribosyl residue led to an increase in cytotoxicity, whereas NH_2_ groups at C2 of the purine had the opposite effect. Complexes with a triphenylphosphane ligand were on average more active than those with triethylphosphane ligands. The cellular uptake of the complexes did not vary much with the residues R^1-3^ with complex **6** sticking out for its conspicuously great uptake. The most cytotoxic complex **5** was investigated in more detail.

Complex **5** exhibited a bipolar effect on the cancer cell cycle, leading to G1-phase arrest, like other known thiopurines, at low concentrations and to S-phase arrest, like auranofin, at higher concentrations. The latter effect is very likely due to its pronounced inhibition of TrxR. Complex **5** also initiated severe DNA lesions and fragmentation, ultimately leading to cancer cell apoptosis.

## Supplementary Information

Below is the link to the electronic supplementary material.

Supplementary file1 1H, 13C, 31P and if applicable 77Se-NMR, as well as HPL-chromatograms of compounds 1–10; stability tests via 1H-NMR for compounds 1–10; stability tests via 31P-NMR for compounds 4 and 7; stability tests via 77Se-NMR for compound 10; stability tests of 1 and 5 via UV/Vis spectroscopy; influence on tubulin polymerization of 6-MP, 6-TG, Auranofin, CA-4 in comparison with complexes 5 and 6. This material is available free of charge via the Internet at https://link.springer.com (PDF 4830 KB)
