# Supplementary material for: Revisiting the anticancer properties of phosphane(9-ribosylpurine-6-thiolato)gold(I) complexes and their 9H-purine precursors
Source: J Biol Inorg Chem. 2022 Oct 16;27(8):731–45. doi: 10.1007/s00775-022-01968-x (PMC9653339; doi:10.1007/s00775-022-01968-x)
Supplement: Supplementary file 1 — Supplementary file1 1H, 13C, 31P and if applicable 77Se-NMR, as well as HPL-chromatograms of compounds 1–10; stability tests via 1H-NMR for compounds 1–10; stability tests via 31P-NMR for compounds 4 and 7; stability tests via 77Se-NMR for compound 10; stability tests of 1 and 5 via UV/Vis spectroscopy; influence on tubulin polymerization of 6-MP, 6-TG, Auranofin, CA-4 in comparison with complexes 5 and 6. This material is available free of charge via the Internet at https://link.springer.com (PDF 4830 KB) [file 775_2022_1968_MOESM1_ESM.pdf]

-Supplementary data-

Revisiting the anticancer properties of phosphane(9-ribosylpurine-6-thiolato)gold(I) complexes and their 9H-purine precursors

Luisa Kober[+] • Sebastian W. Schleser[+] • Sofia I. Bär • Rainer Schobert[*]

Organic Chemistry Laboratory, University of Bayreuth, Universitaetsstrasse 30, 95440 Bayreuth, Germany.

[+]These authors contributed equally to this work

Corresponding Author: Rainer Schobert, Tel.: +49 (0)921-552679,
E-mail: Rainer.Schobert@uni-bayreuth.de, ORCID 0000-0002-8413-4342.

Conflicts of interest: There are no conflicts of interest.

Source of funding: Deutsche Forschungsgemeinschaft grant Scho 402/12-2;

Table of Content

Induction of apoptosis and necrosis ....- 3 -

Influence on tubulin polymerization ....- 4 -

Cellular uptake ....- 5 -

Dose-Response Curves of **5** ....- 5 -

NMR spectra of complexes **1-10** ....- 6 -

Stability testing via ^{1}H, ^{31}P and ^{77}Se-NMR spectroscopy ....- 22 -

Stability testing via UV/Vis spectroscopy ....- 28 -

HPLC Chromatograms ....- 29 -

Induction of apoptosis and necrosis

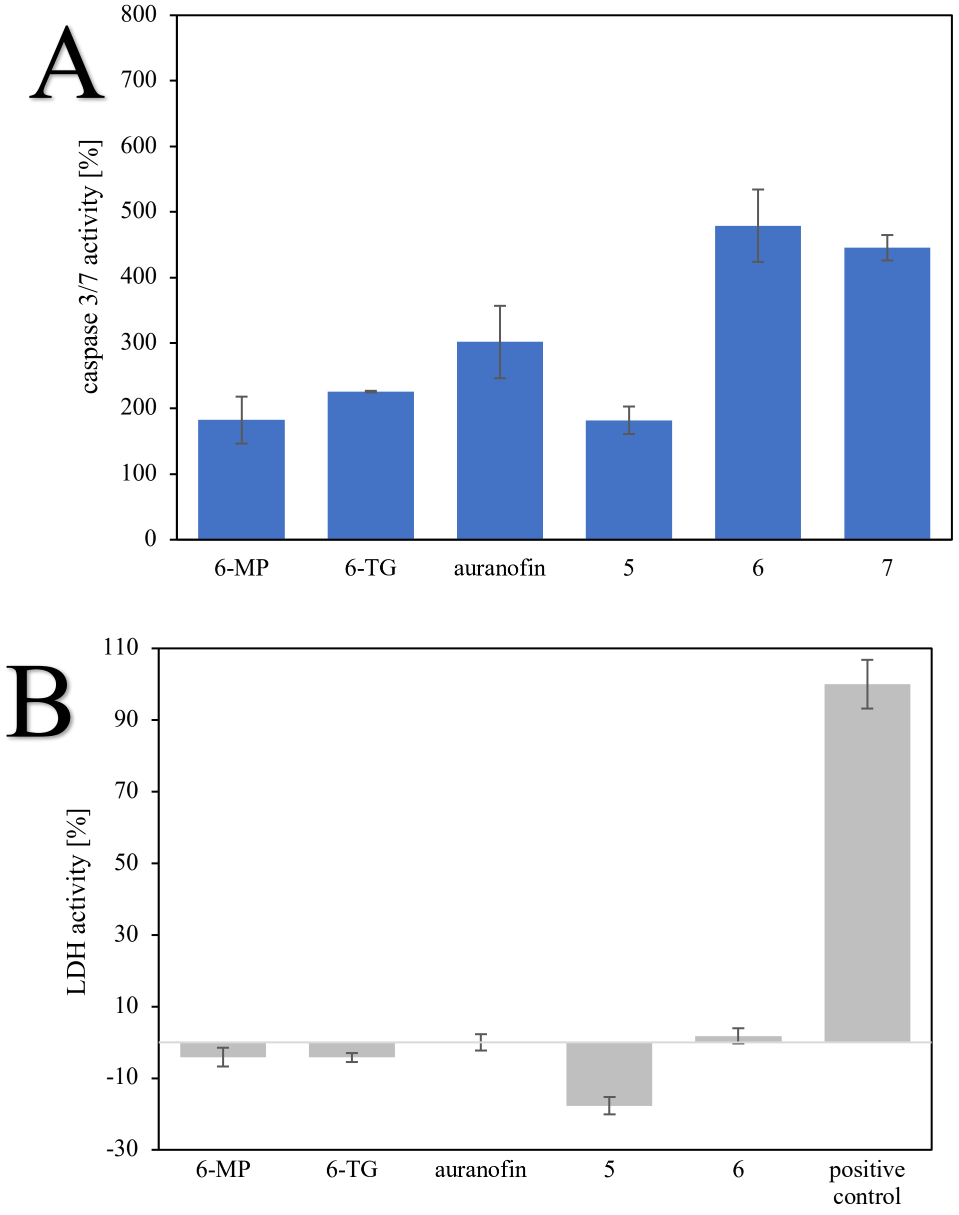

Figure S1. **A** Induction of effector capase-3/-7 activity in 518A2 melanoma cells after treatment with 10 µM mercaptopurine, thioguanine, auranofin and complexes **5**–**7** for 6 h. Vitality of cells was simultaneously tested by MTT assays and was found to be > 85 % for all experiments which were performed in triplicate. Results are quoted as means ± SD. The solvent-treated negative control was set to 100%. **B** Release of intracellular LDH in 518A2 melanoma cells after treatment with 10 µM mercaptopurine, thioguanine, auranofin and complexes **5** and **6** for 24 h. All experiments were performed in at least four independent experiments. Values are means ± SD with positive control, which displayed maximum LDH release, was set to 100%.

Influence on tubulin polymerization

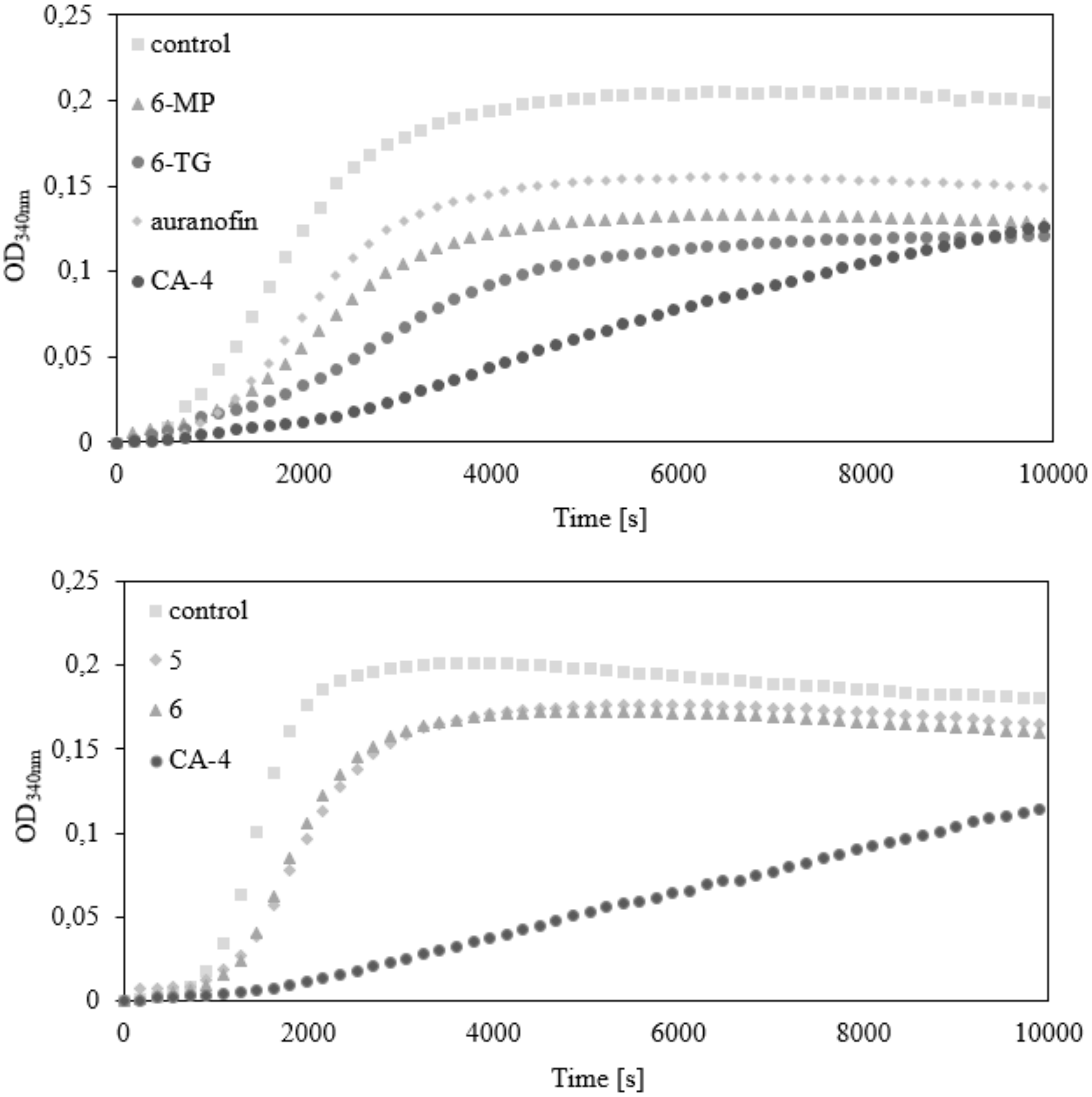

Figure S2. Inhibition of tubulin polymerization by mercaptopurine, thioguanine, auranofin, complexes **5**–**6** and CA-4 as a positive control (10 µM). All experiments were performed as duplicates.

Cellular uptake

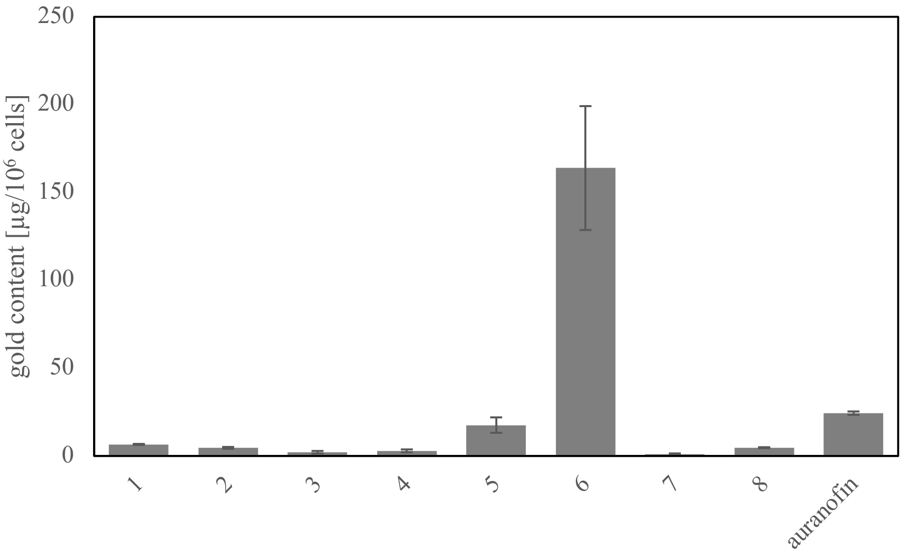

Figure S3. Cellular uptake [µg/10^6 cells] of complexes **1**–**8** and auranofin in 518A2 melanoma cells. Values were derived from substance treated lysed cells after 5 h of incubation measured via ICP-MS. Values are means ± SD from two independent experiments.

Dose-Response Curves of 5

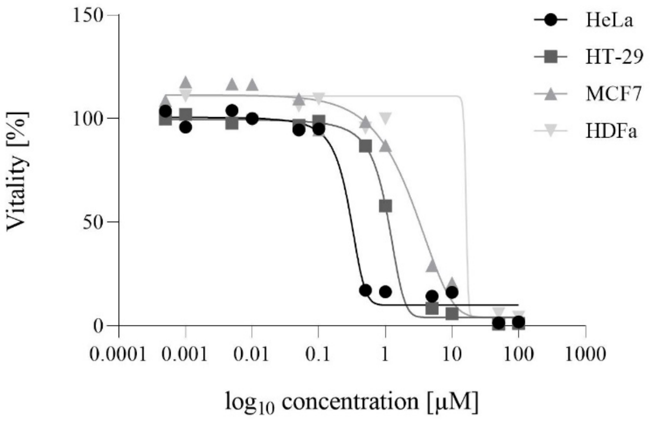

Figure S4. Dose-response curves of complex **5** with various human cancer cell lines as well as HDFa cells.

NMR spectra of complexes **1-10**

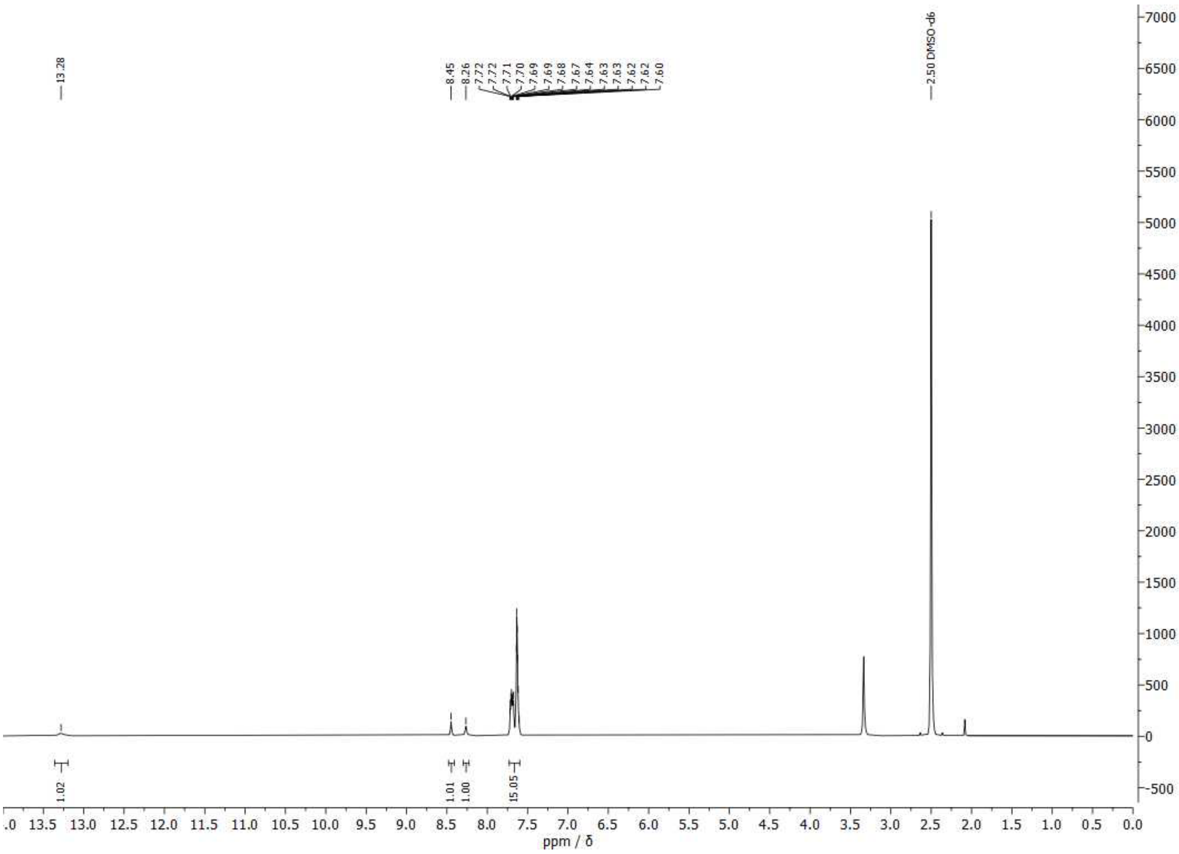

Figure S5. ^{1}H NMR spectrum of **1** in DMSO-d_6

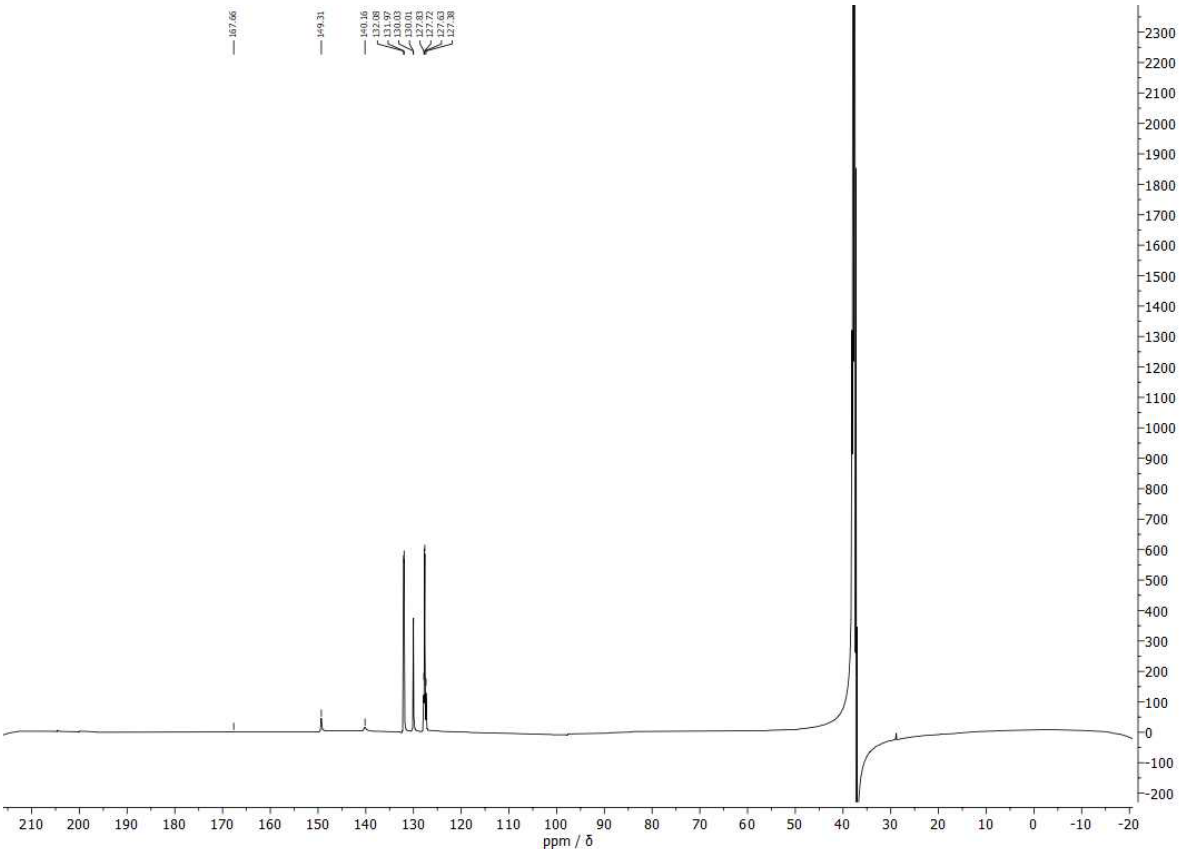

Figure S6. ^{13}C NMR spectrum of **1** in DMSO-d_6.

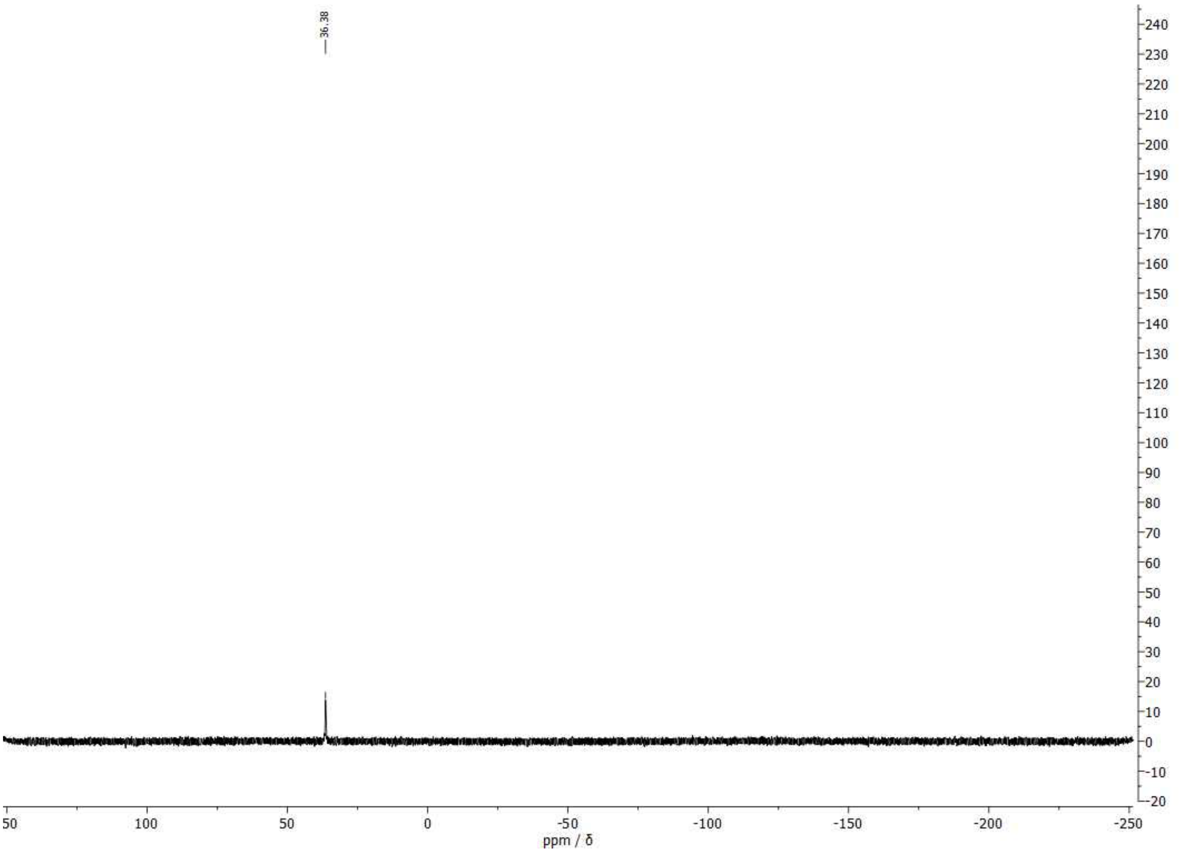

Figure S7. ^{31}P NMR spectrum of **1** in DMSO-d_6.

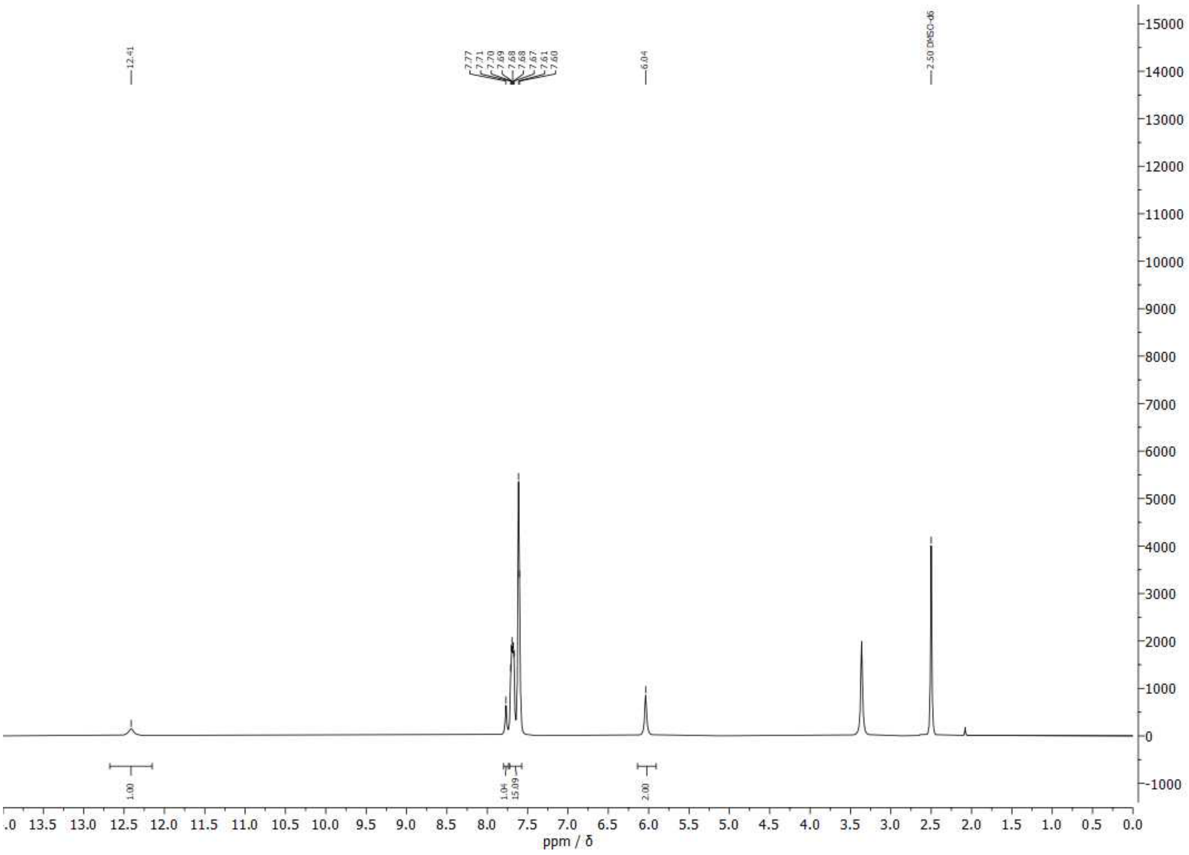

Figure S8. ^{1}H NMR spectrum of **2** in DMSO-d_6.

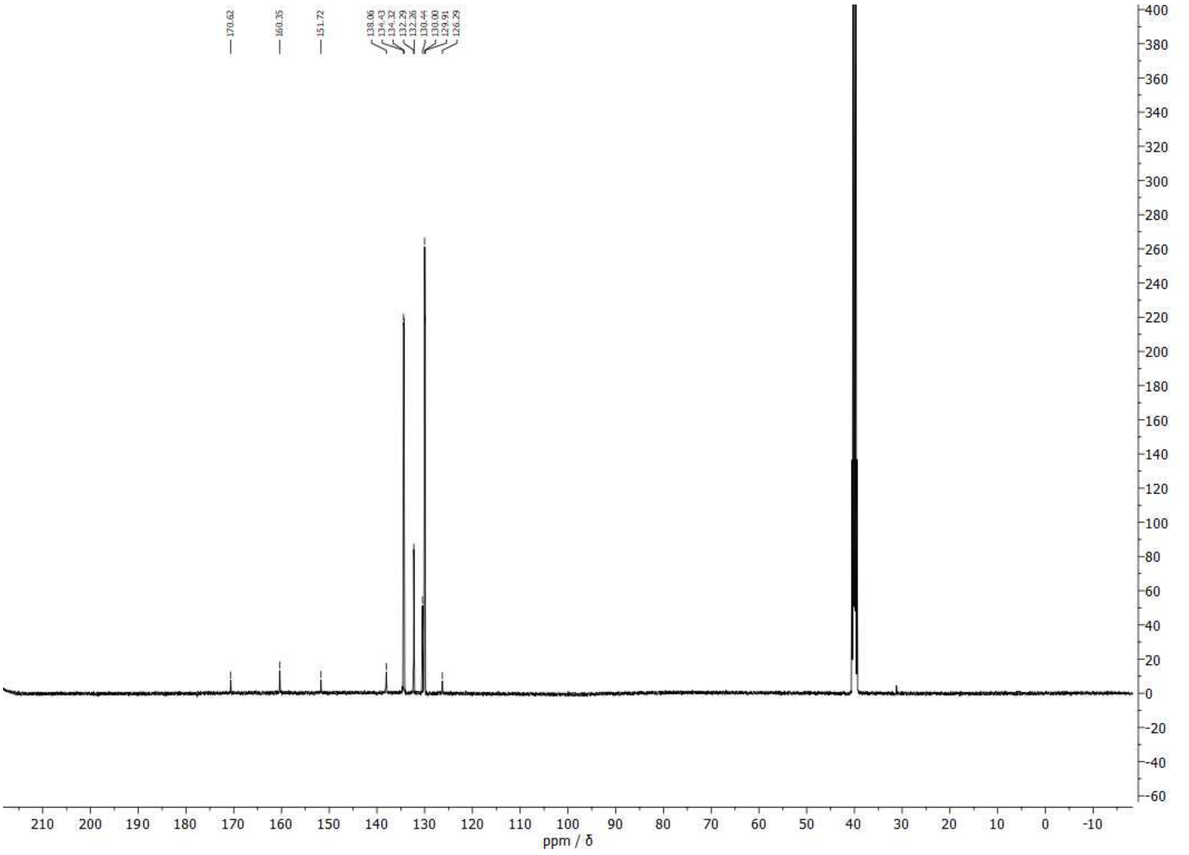

Figure S9. ^{13}C NMR spectrum of **2** in DMSO-d_6.

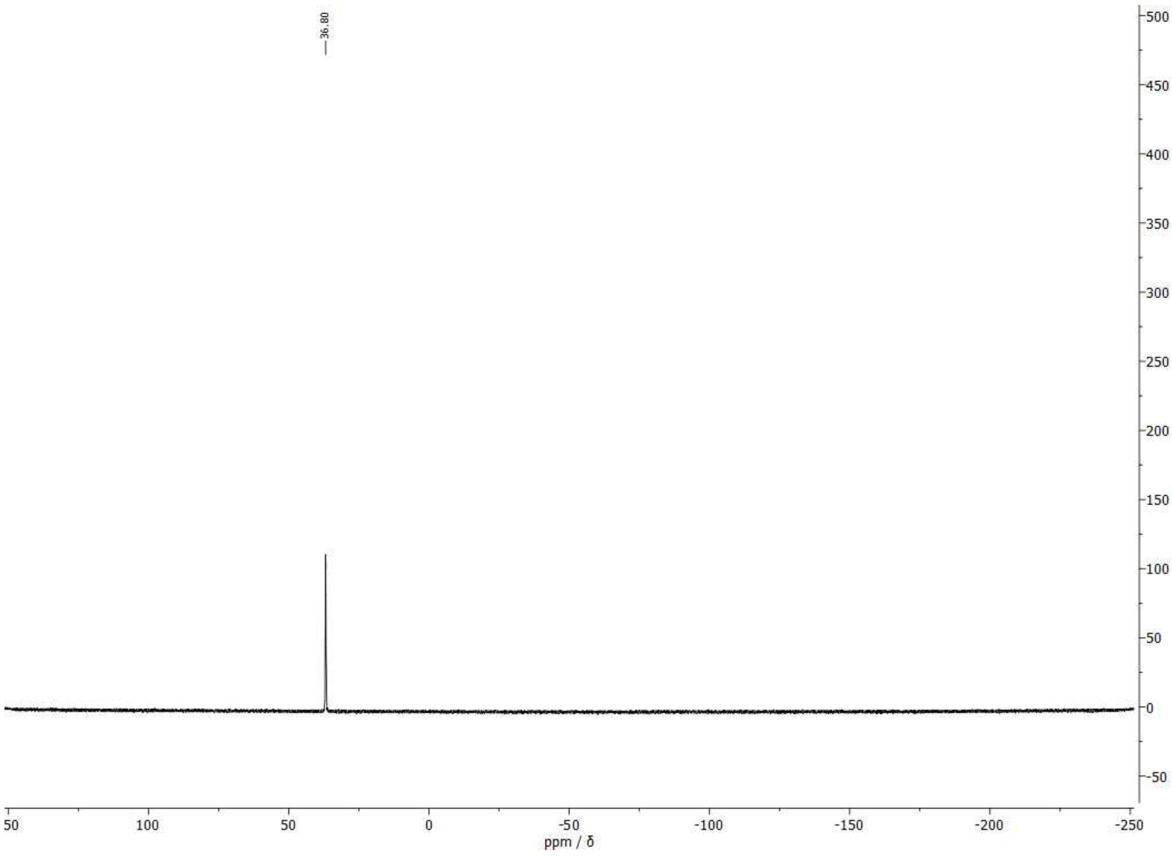

Figure S10. ^{31}P NMR spectrum of **2** in DMSO-d_6.

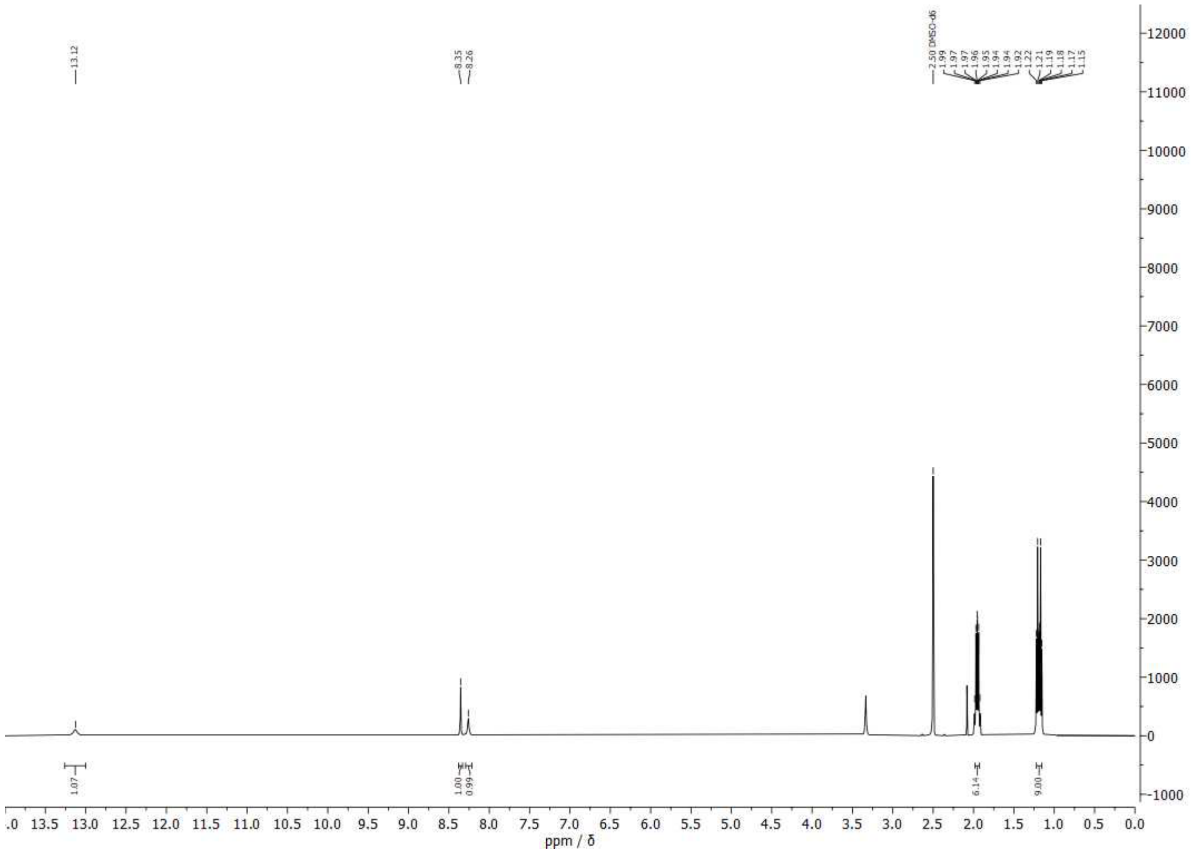

Figure S11. ^{1}H NMR spectrum of **3** in DMSO-d_6.

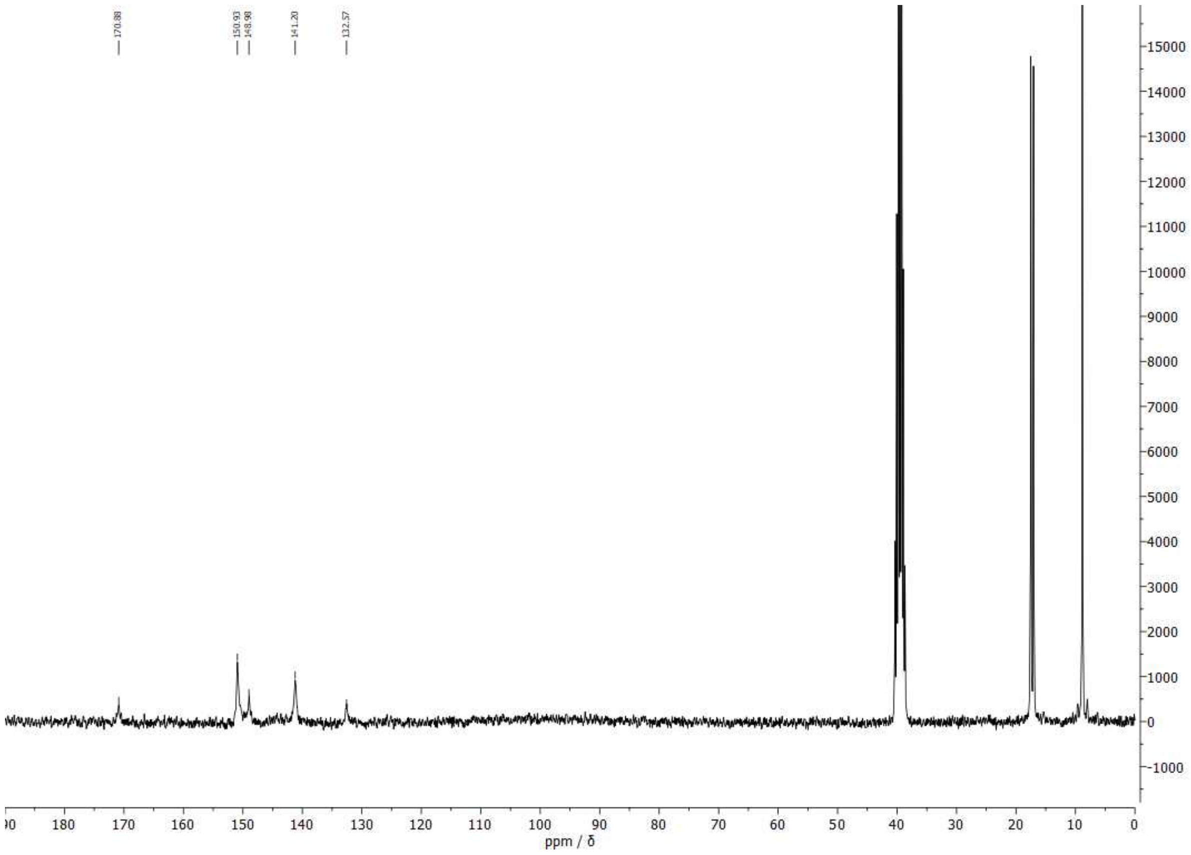

Figure S12. ^{13}C NMR spectrum of **3** in DMSO-d_6.

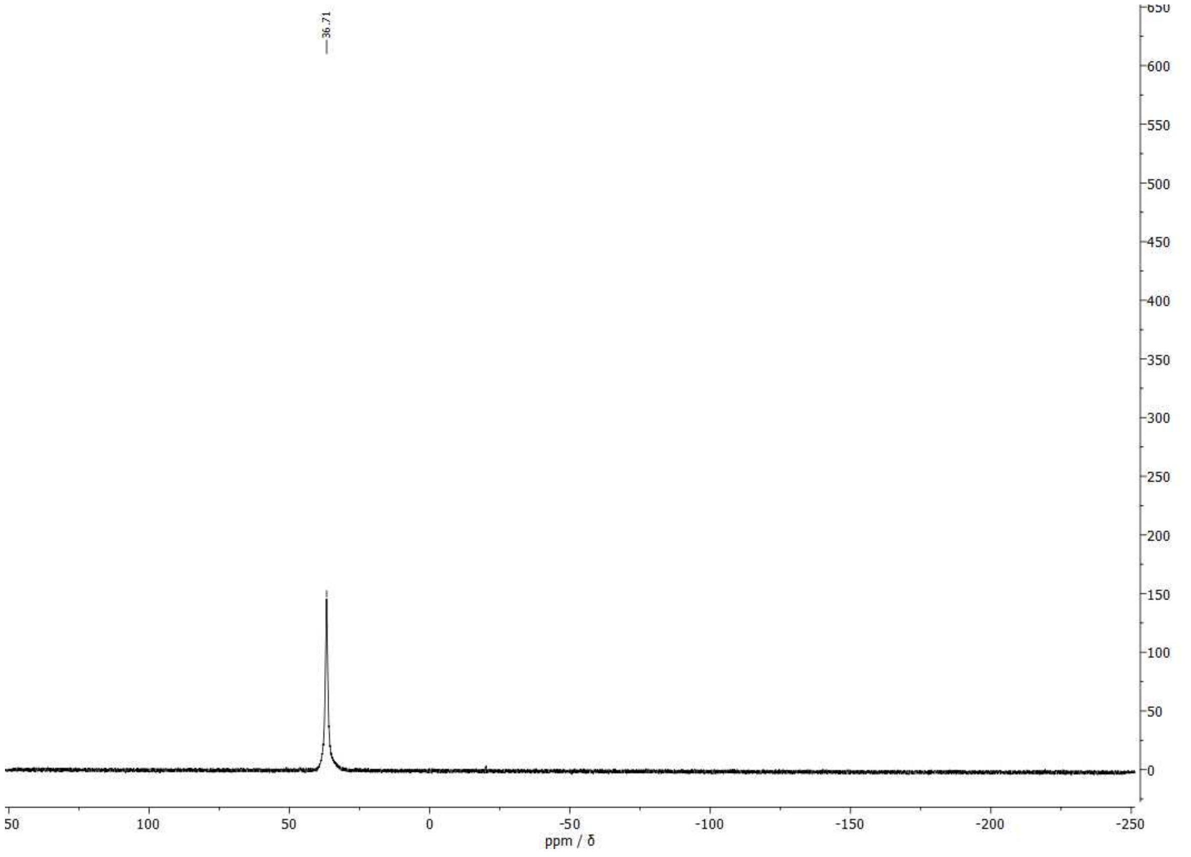

Figure S13. ^{31}P NMR spectrum of **3** in DMSO-d_6.

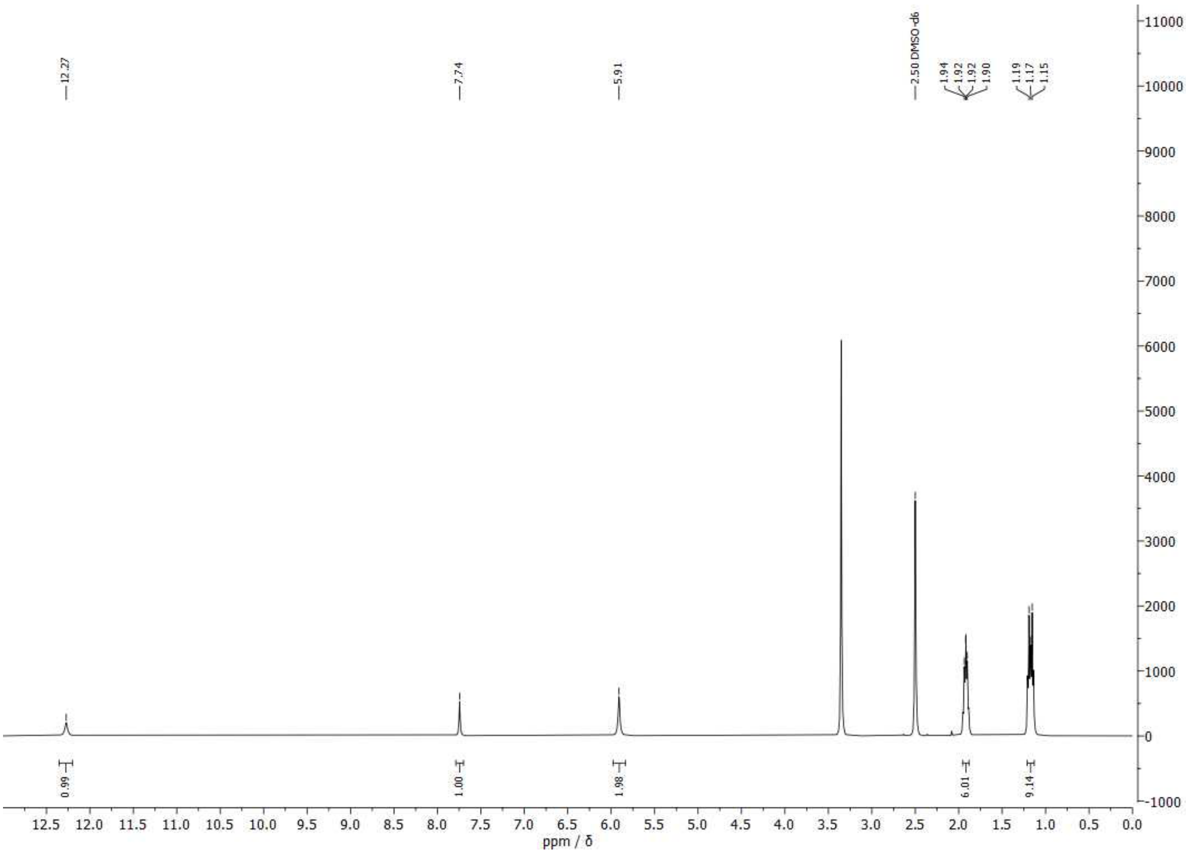

Figure S14. ^{1}H NMR spectrum of **4** in DMSO-d_6.

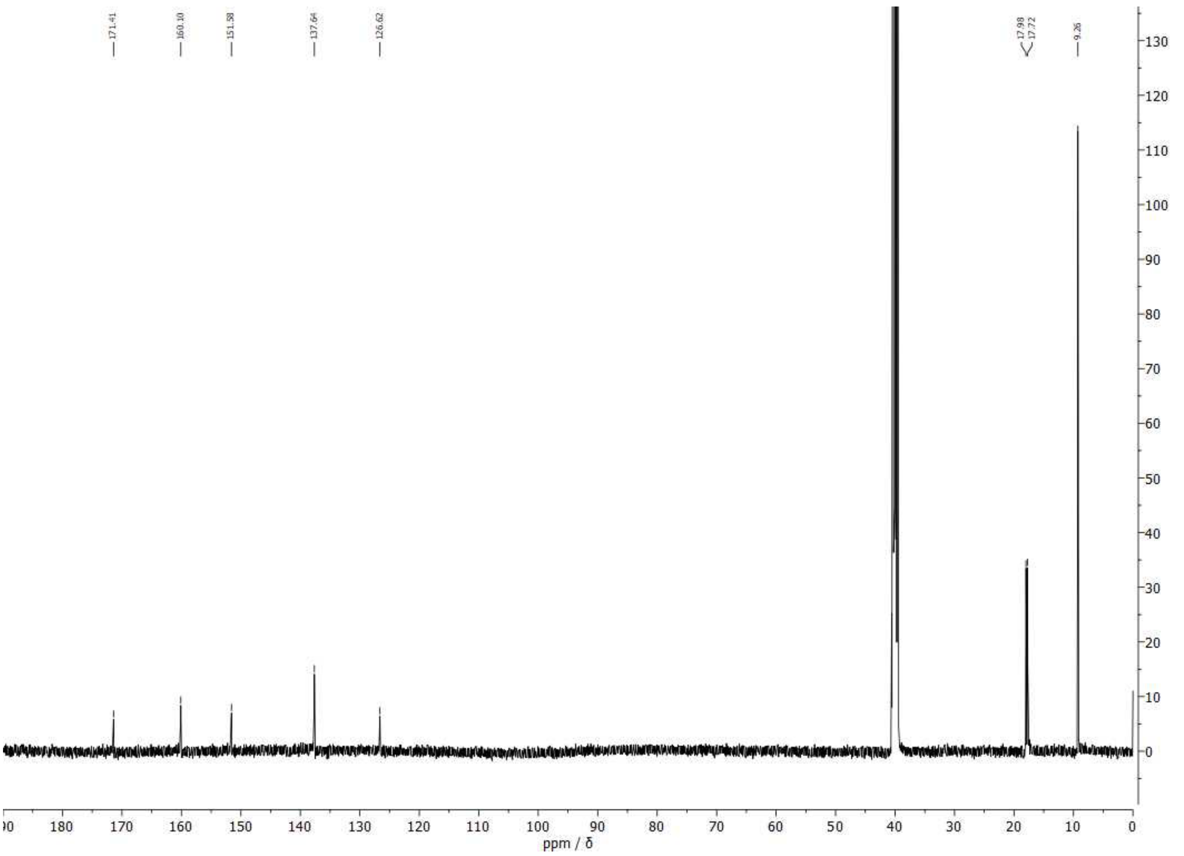

Figure S15. ^{13}C NMR spectrum of **4** in DMSO-d_6.

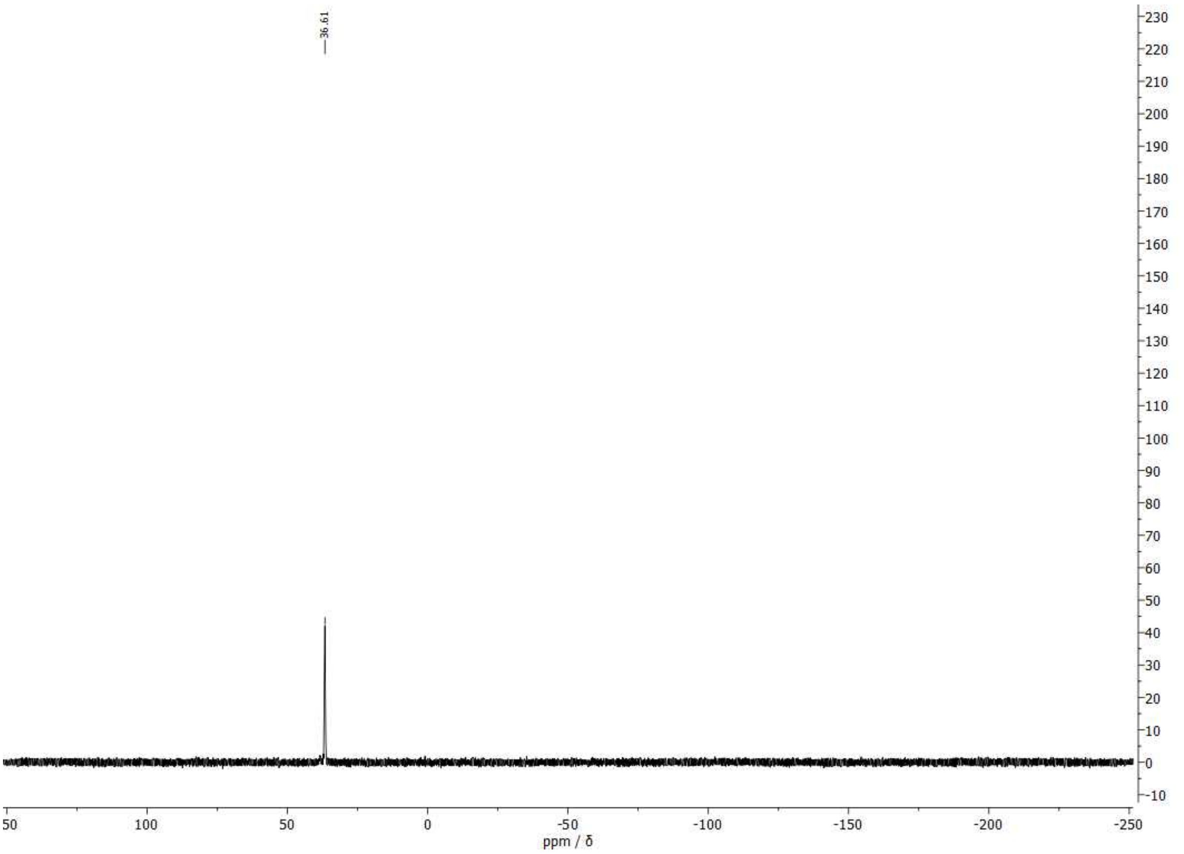

Figure S16. ^{31}P NMR spectrum of **4** in DMSO-d_6.

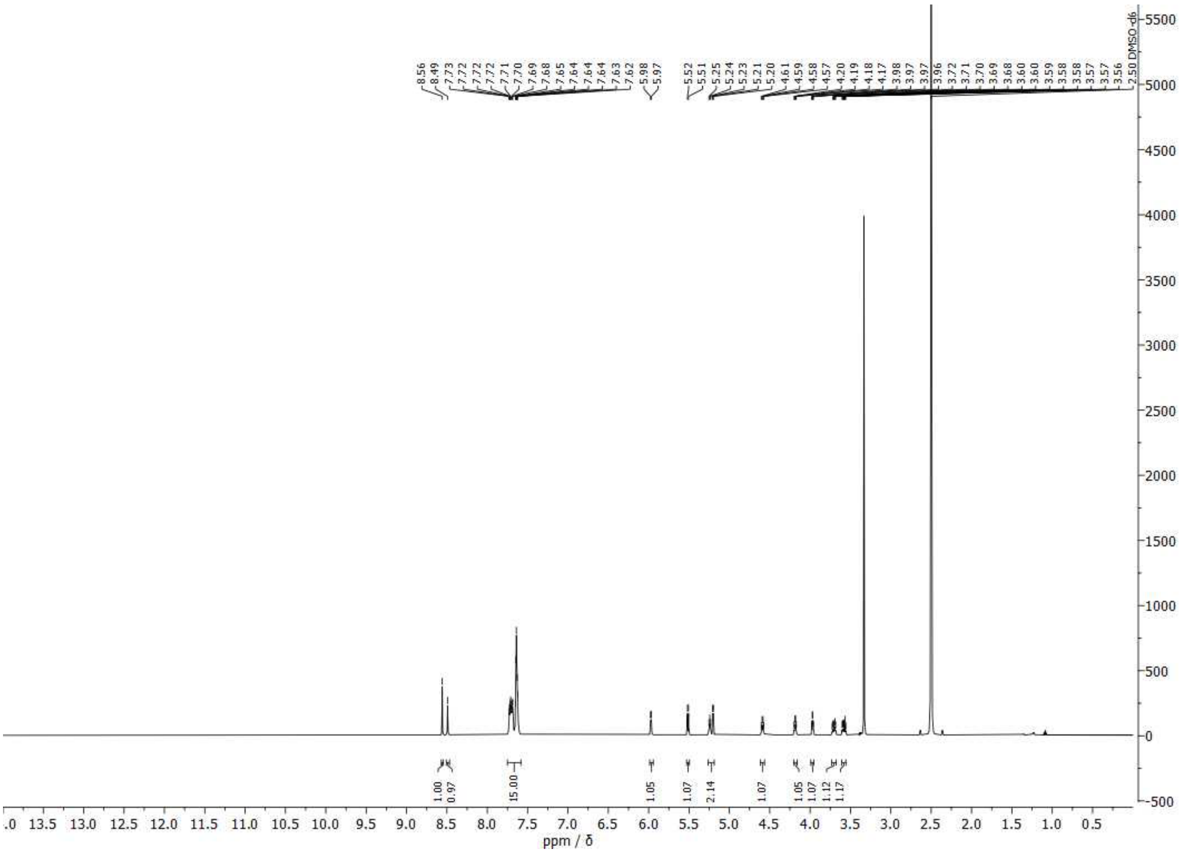

Figure S17. ^{1}H NMR spectrum of **5** in DMSO-d_6.

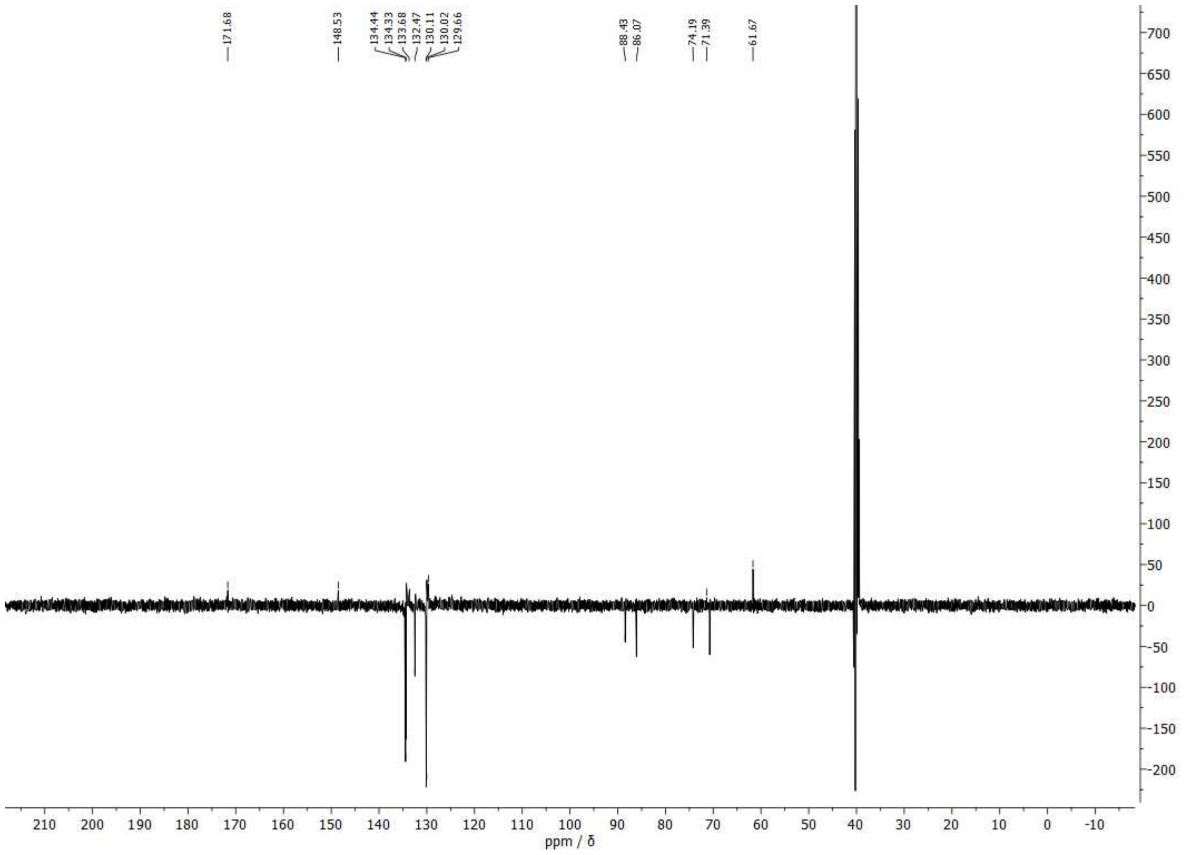

Figure S18. ^{1}H NMR spectrum of **5** in DMSO-d$_6$.

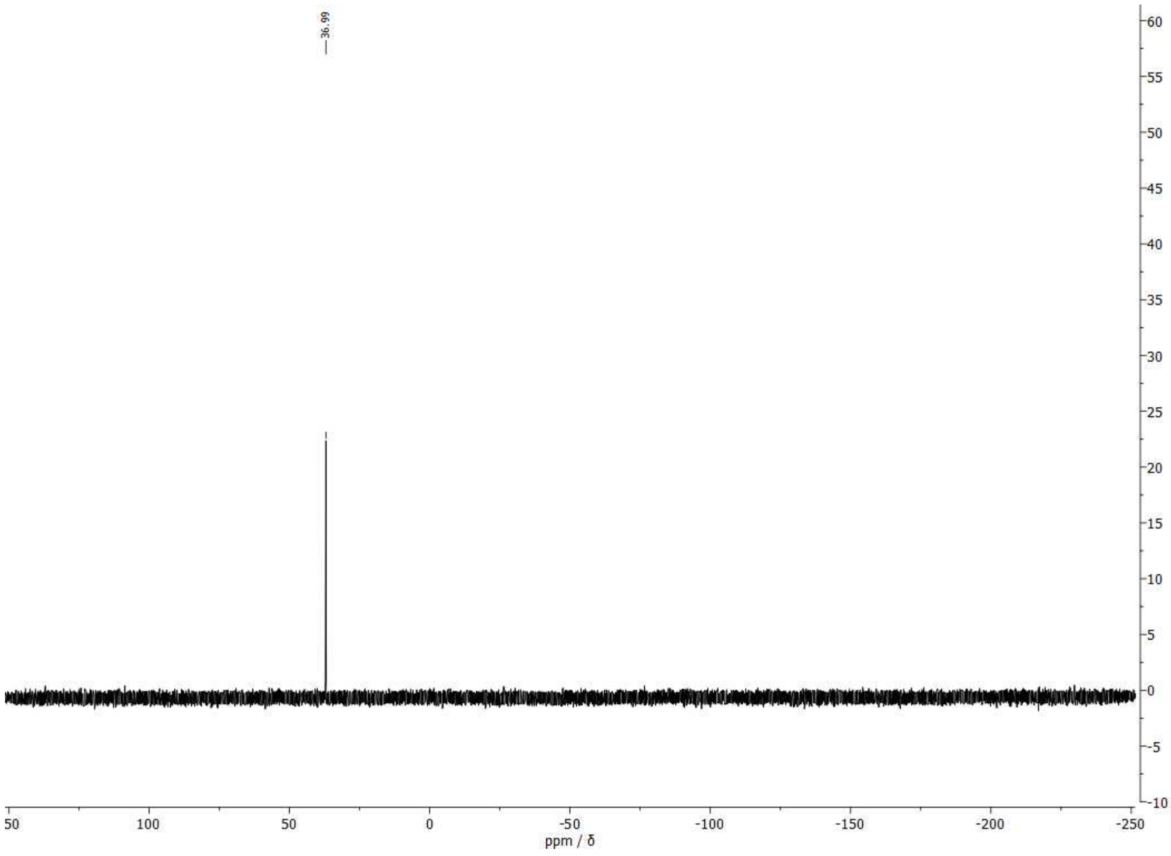

Figure S19. ^{31}P NMR spectrum of **5** in DMSO-d$_6$.

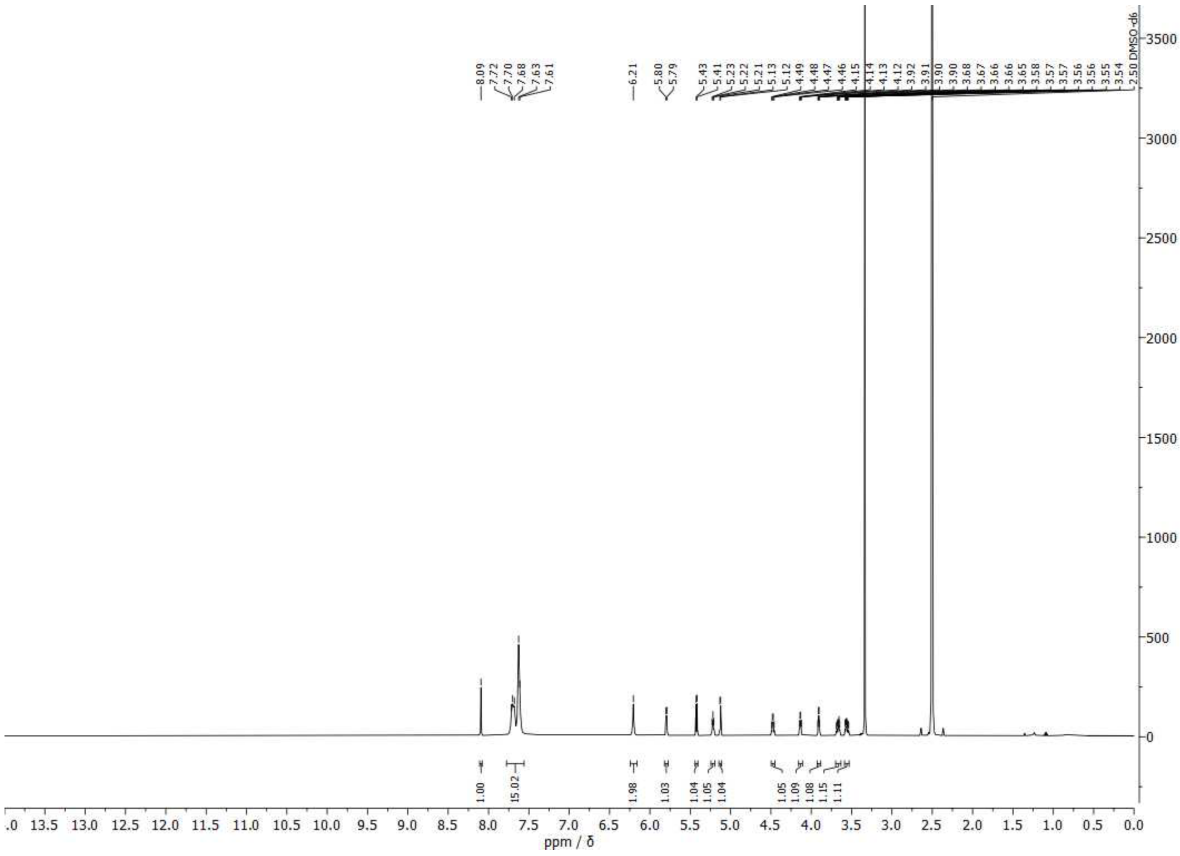

Figure S20. ^{1}H NMR spectrum of **6** in DMSO-d_6.

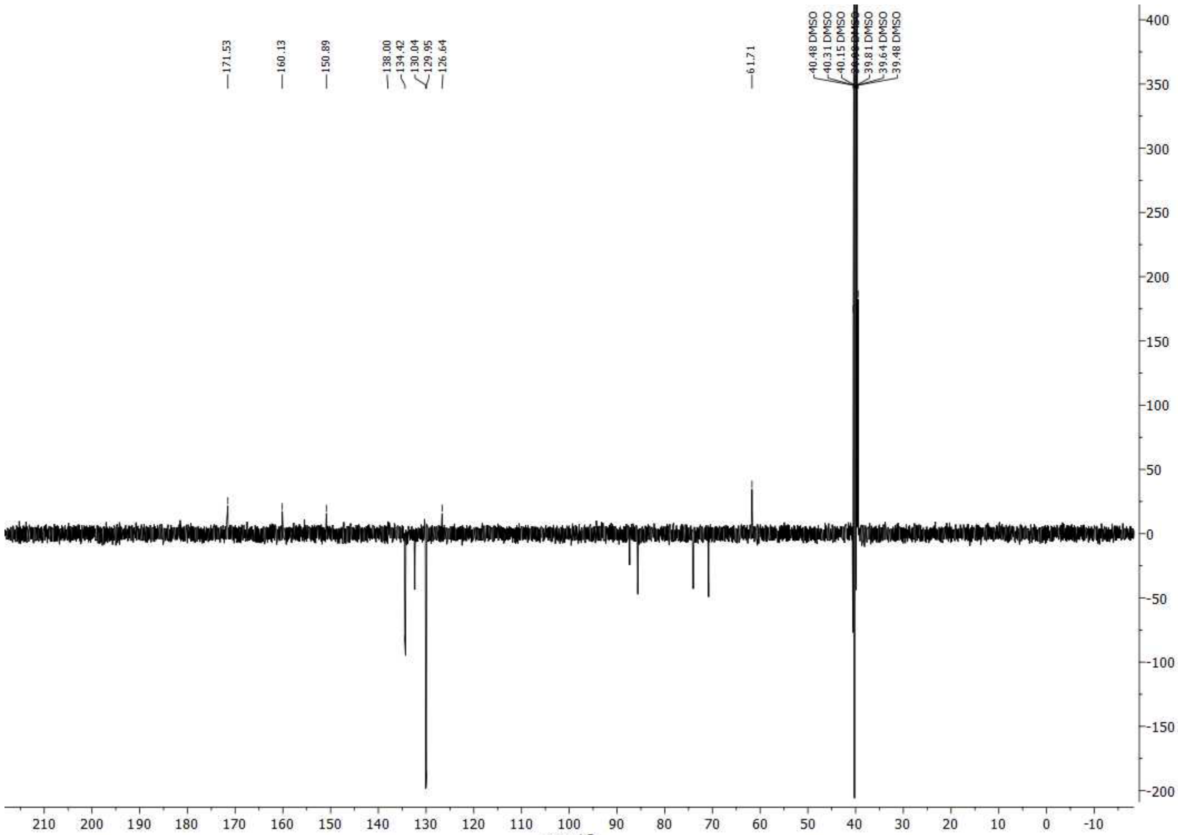

Figure S21. ^{1}H NMR spectrum of **6** in DMSO-d_6.

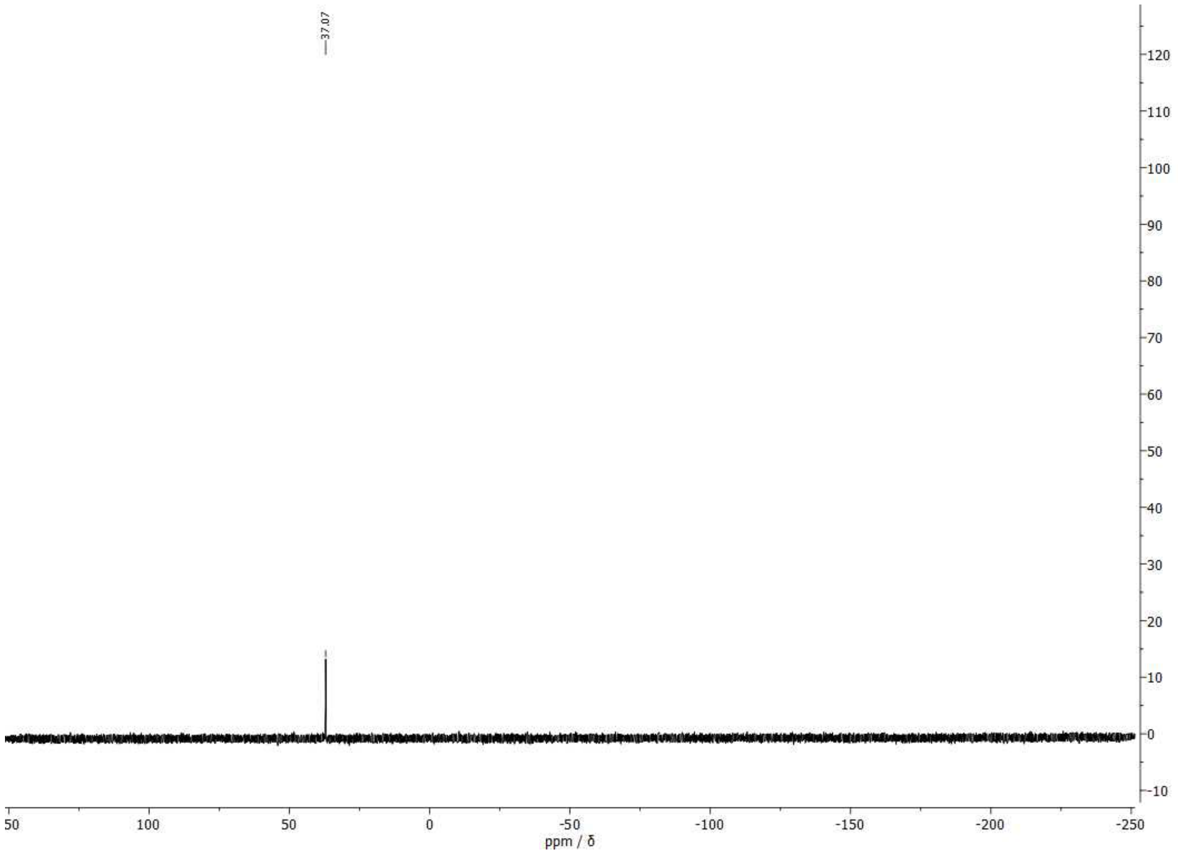

Figure S22. ^{31}P NMR spectrum of **6** in DMSO-d_6.

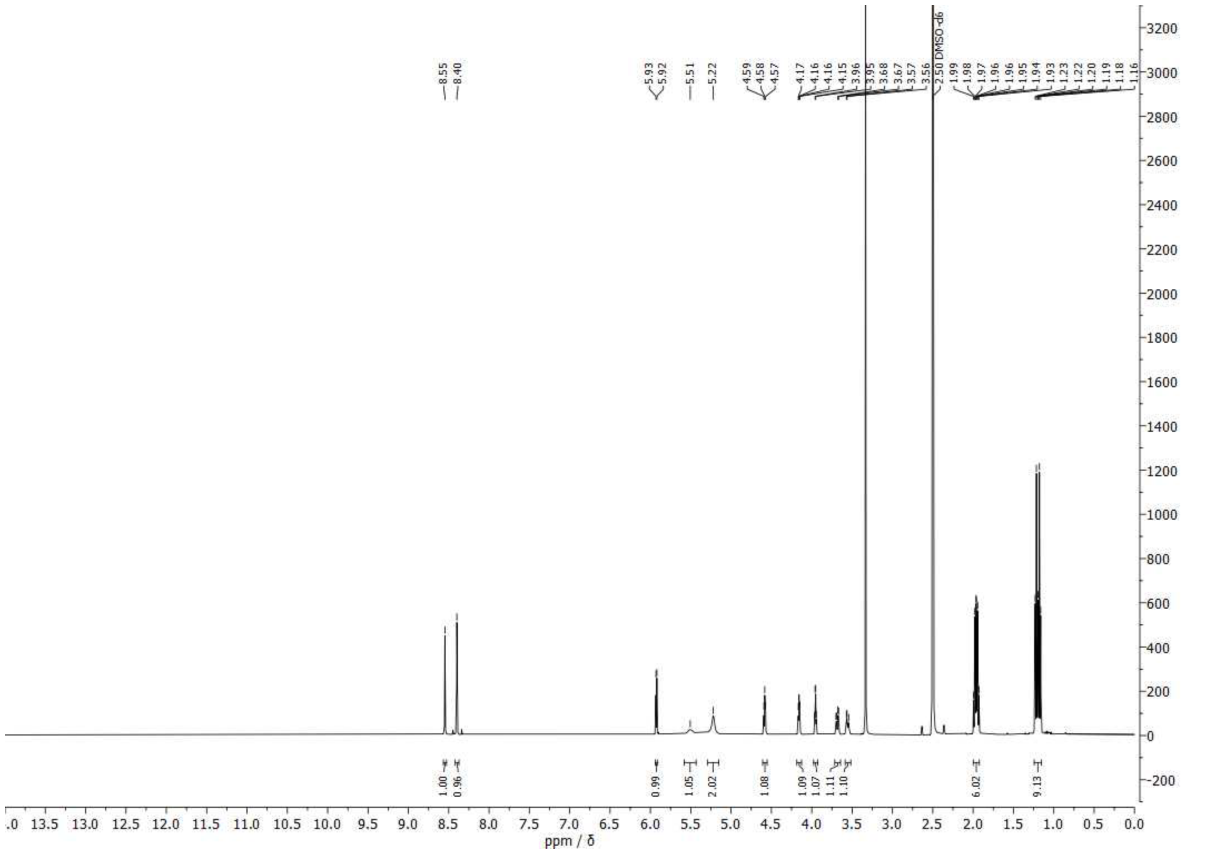

Figure S23. ^{1}H NMR spectrum of **7** in DMSO-d_6.

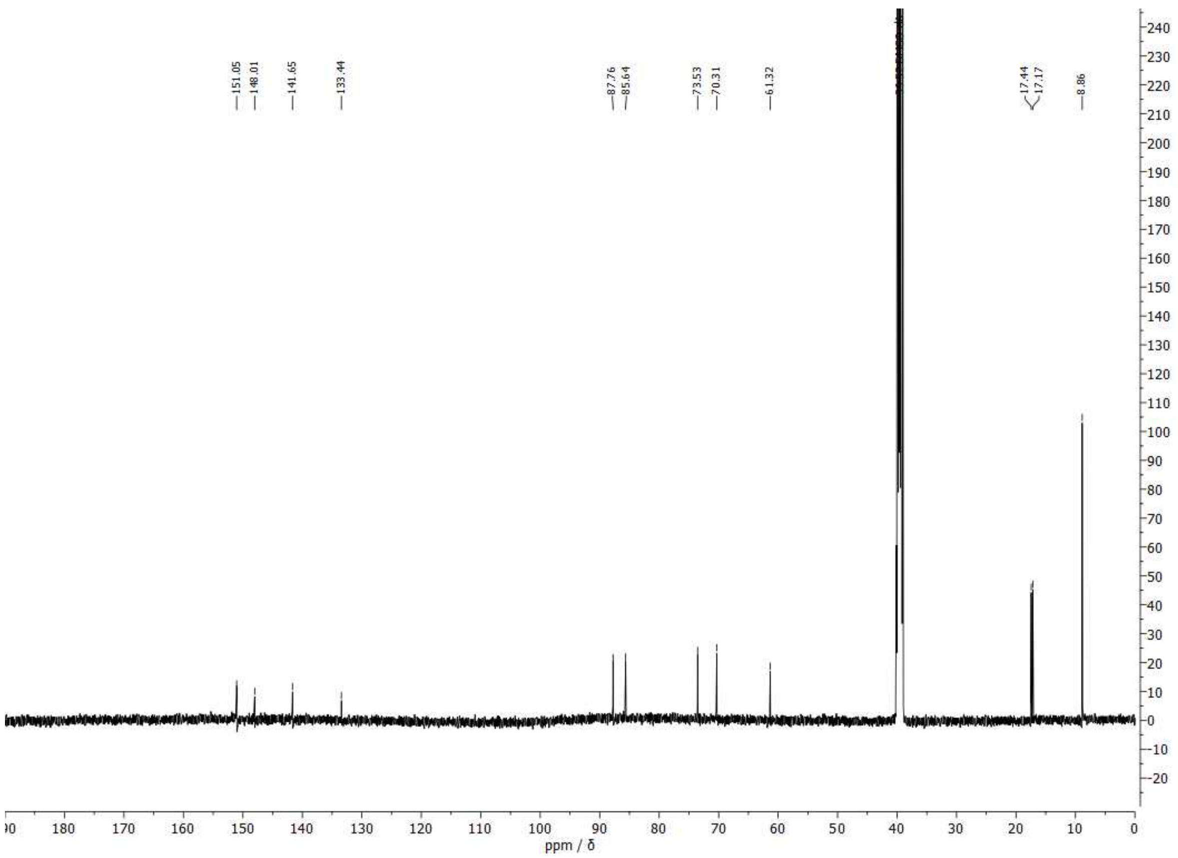

Figure S24. ^{13}C NMR spectrum of **7** in DMSO-d_6.

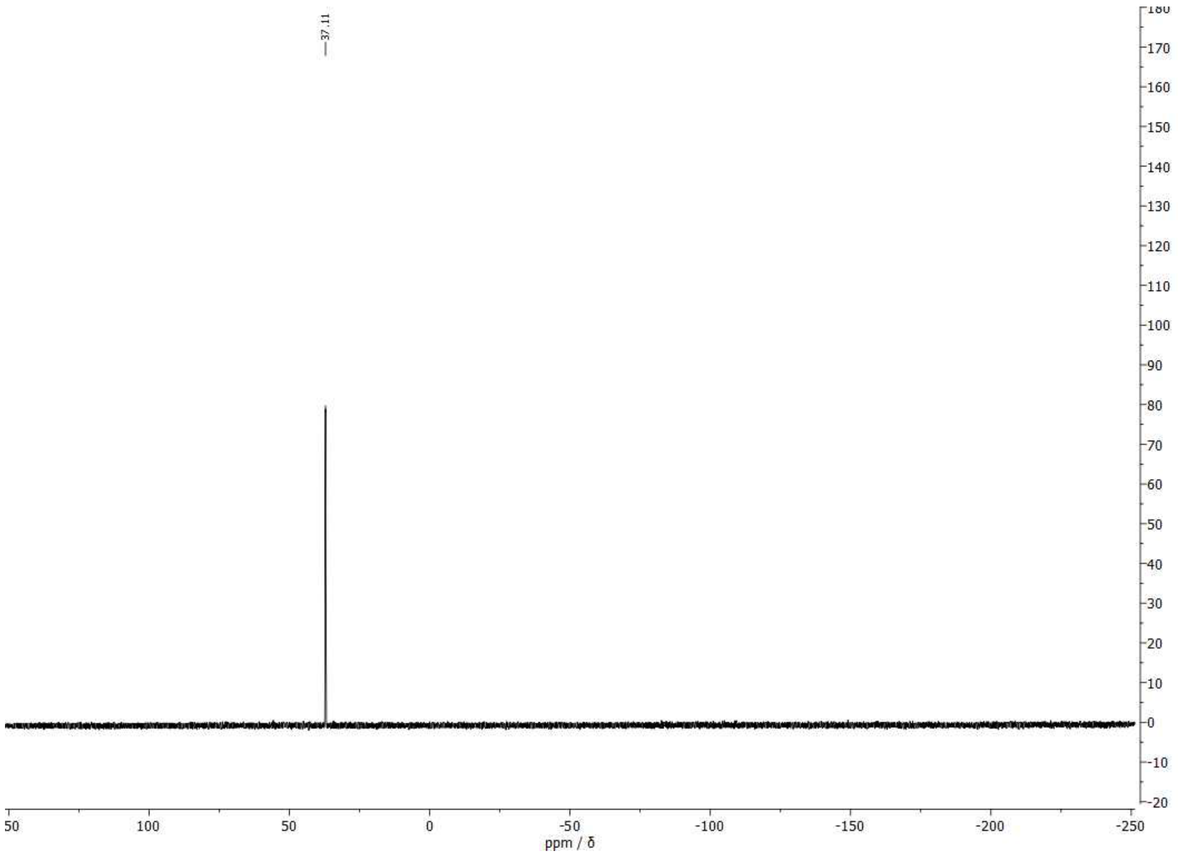

Figure S25. ^{31}H NMR spectrum of **7** in DMSO-d_6.

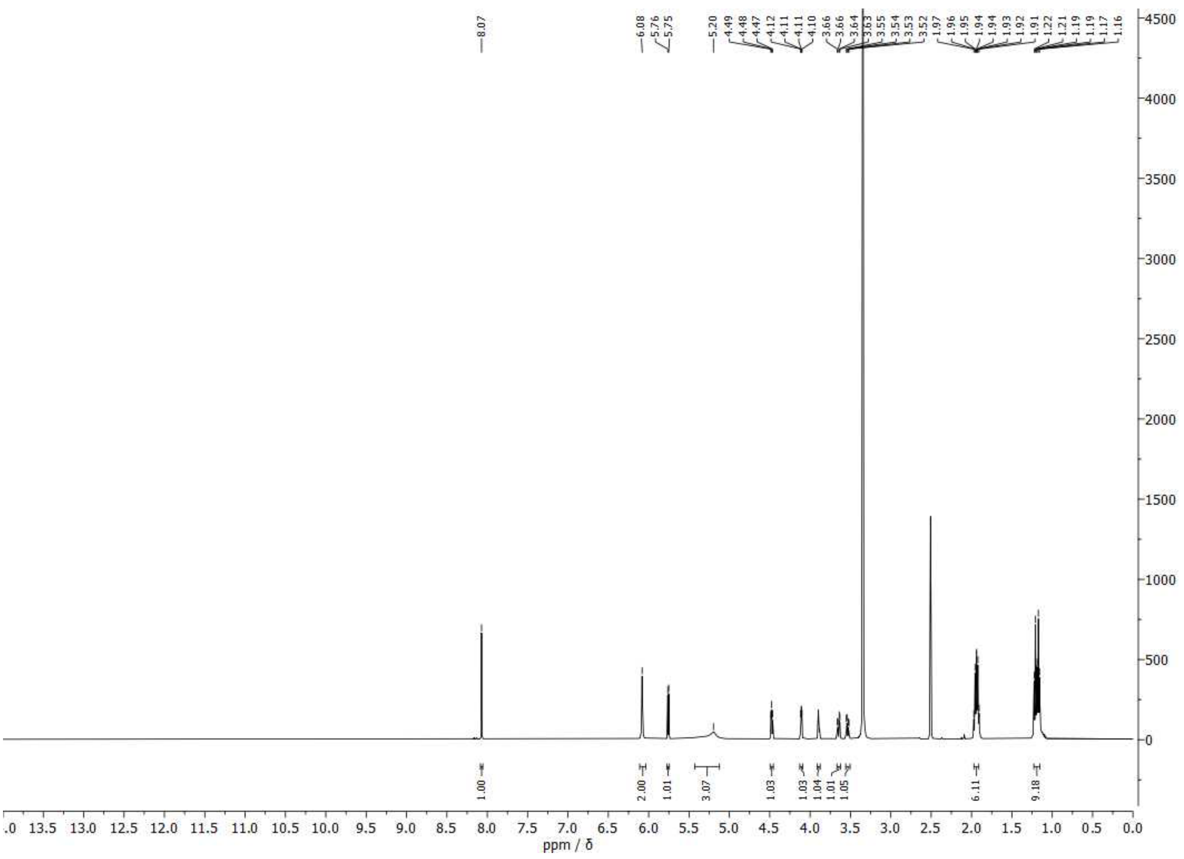

Figure S26. ^{1}H NMR spectrum of **8** in DMSO-d_6.

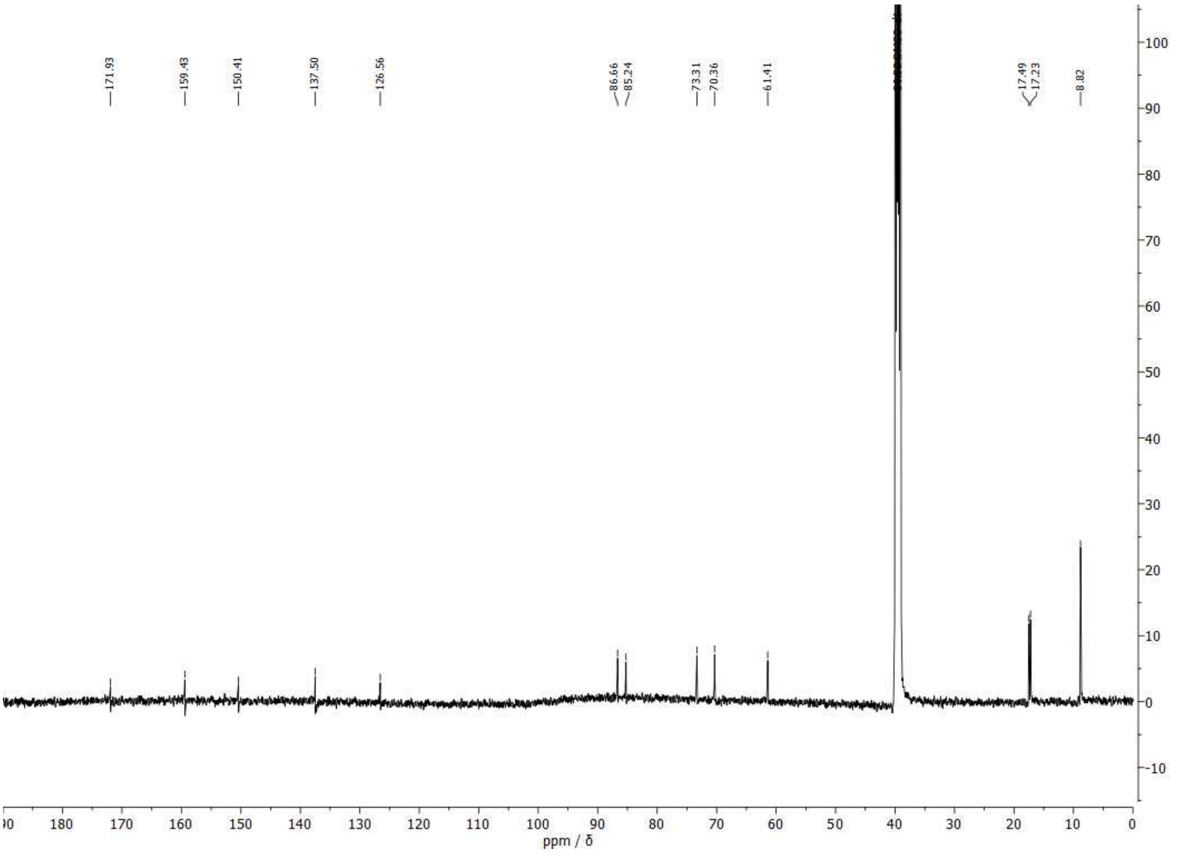

Figure S27. ^{13}C NMR spectrum of **8** in DMSO-d_6.

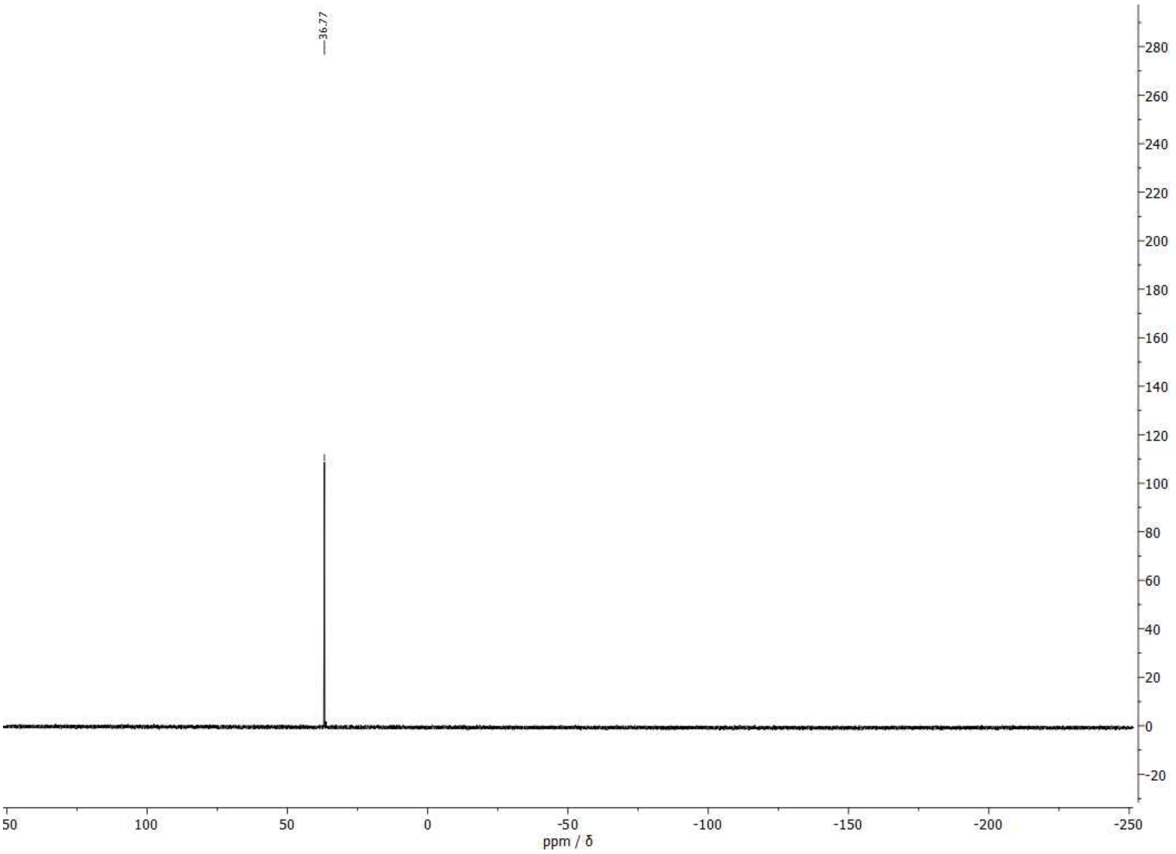

Figure S28. ^{31}P NMR spectrum of **8** in DMSO-d$_6$.

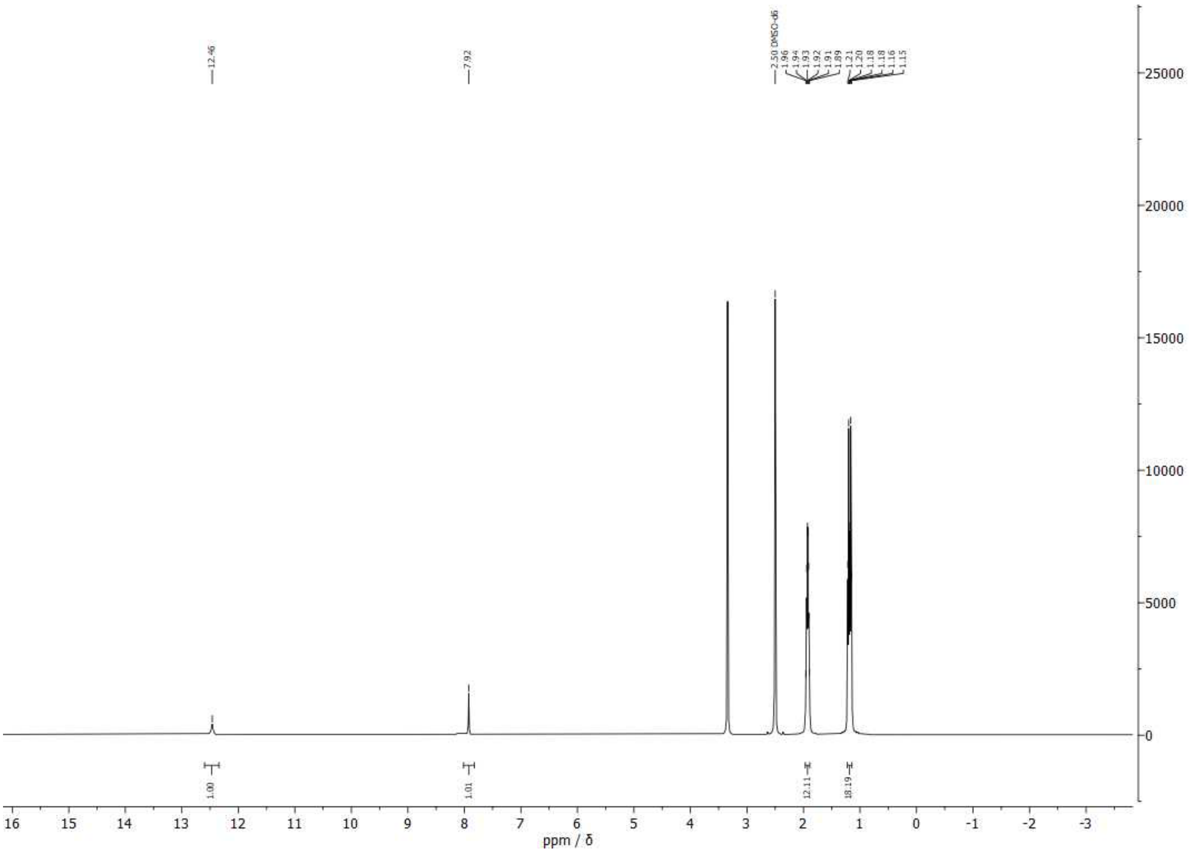

Figure S29. ^{1}H NMR spectrum of **9** in DMSO-d$_6$.

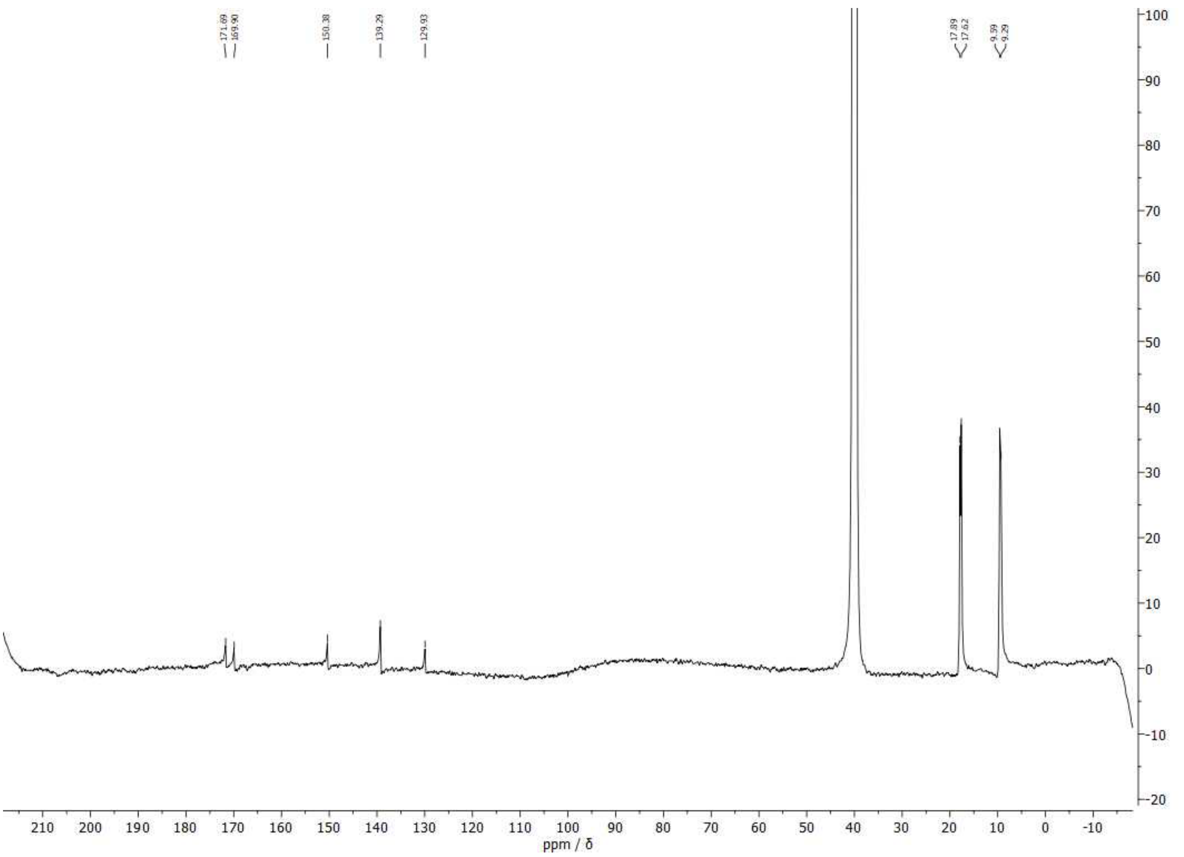

Figure S30. ^{13}C NMR spectrum of **9** in DMSO-d_6.

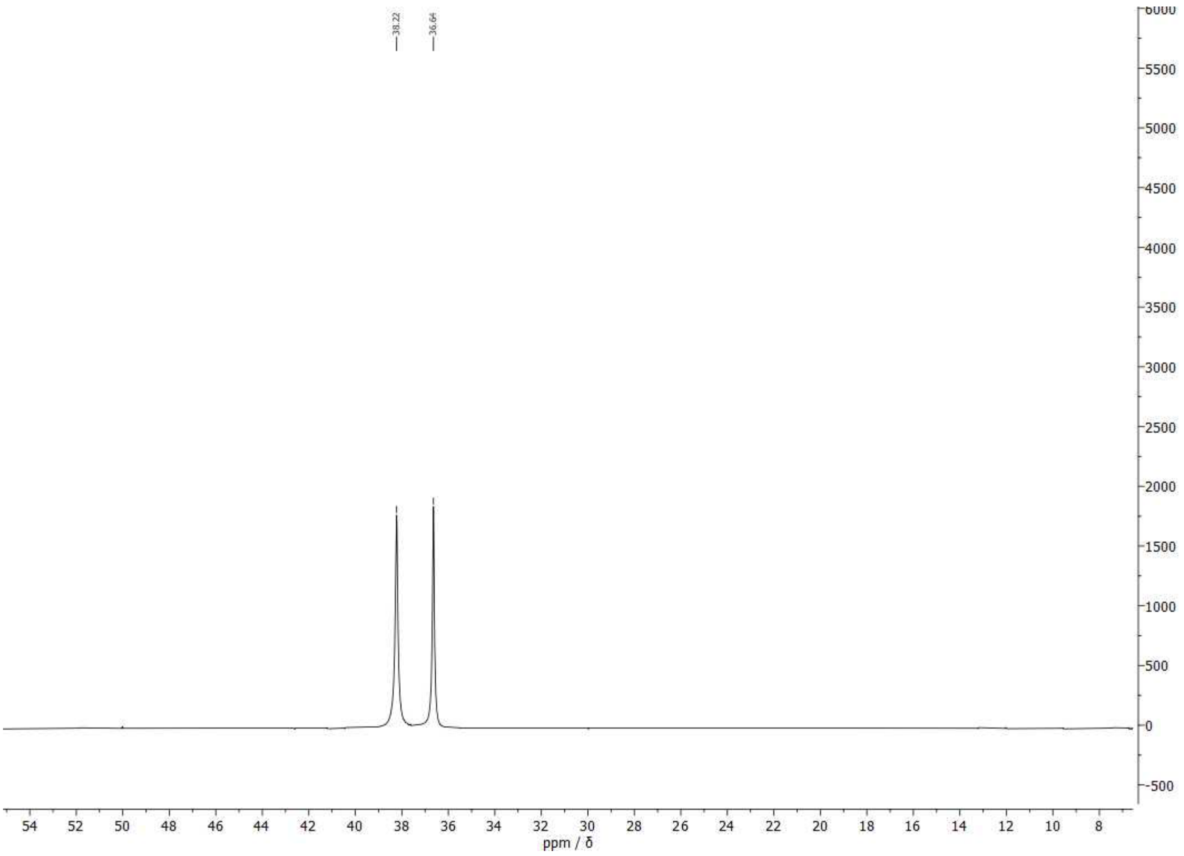

Figure S31. ^{31}P NMR spectrum of **9** in DMSO-d_6.

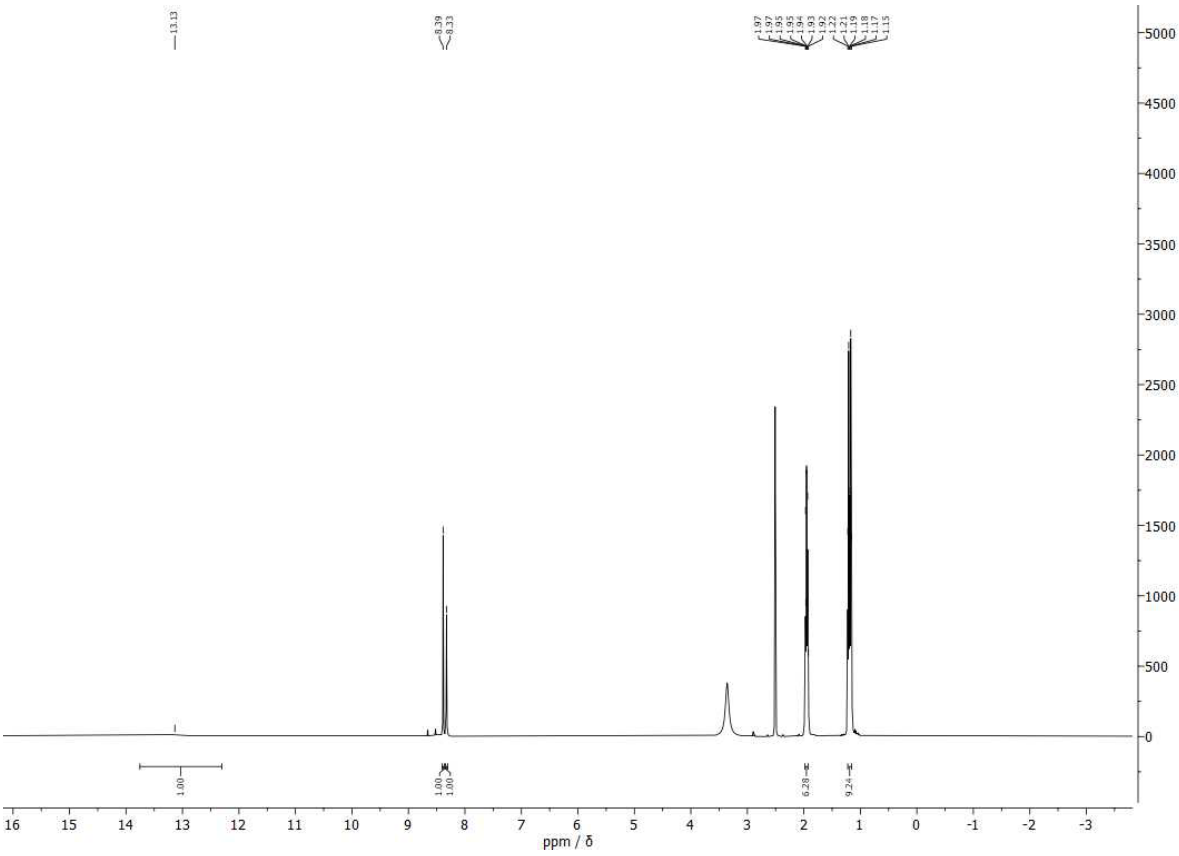

Figure S32. ^{1}H NMR spectrum of **10** in DMSO-d_6.

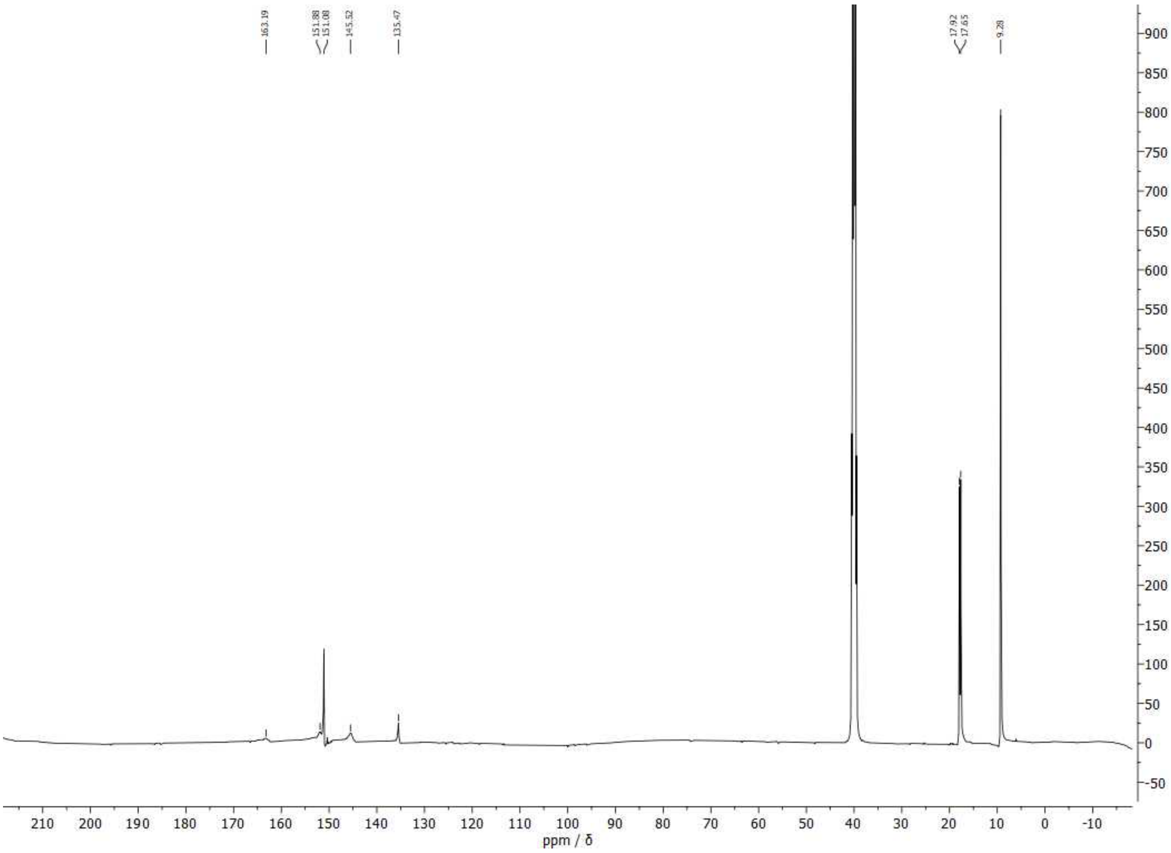

Figure S33. ^{13}C NMR spectrum of **10** in DMSO-d_6.

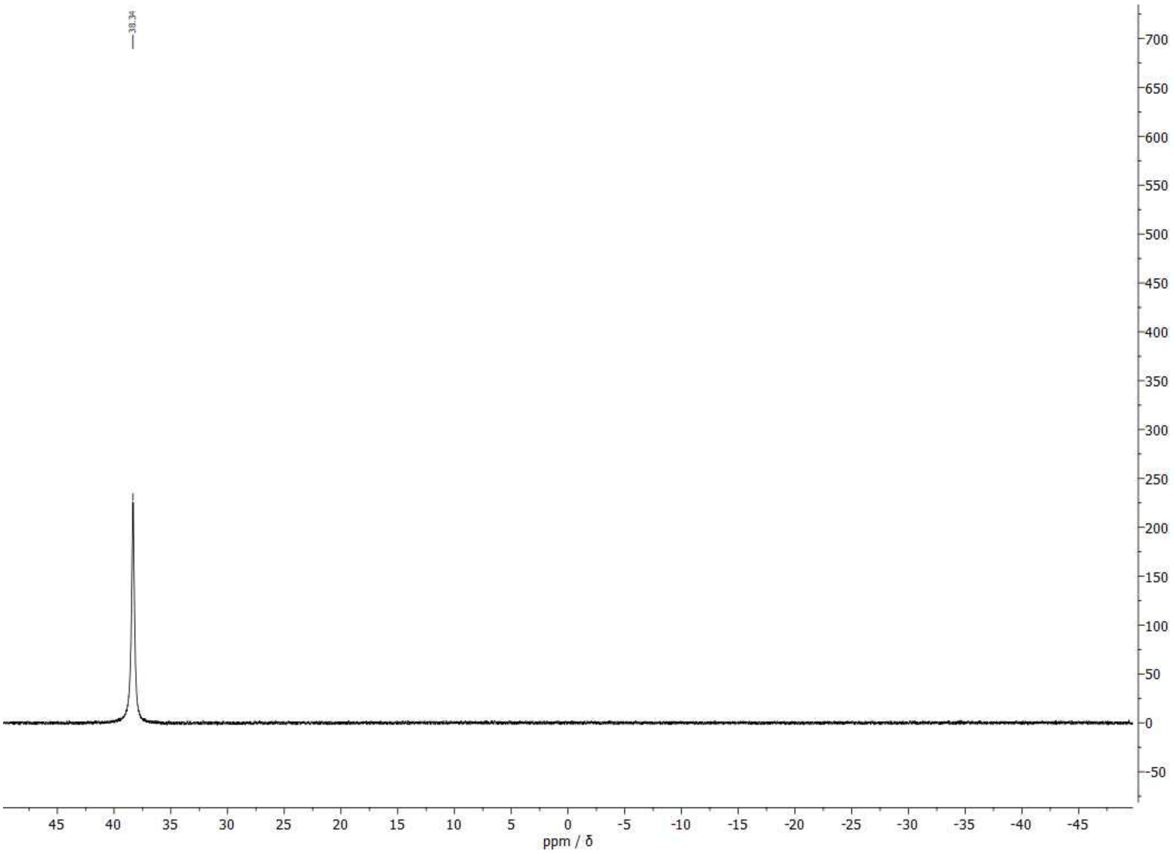

Figure S34. ^{31}P NMR spectrum of **10** in DMSO-d_6.

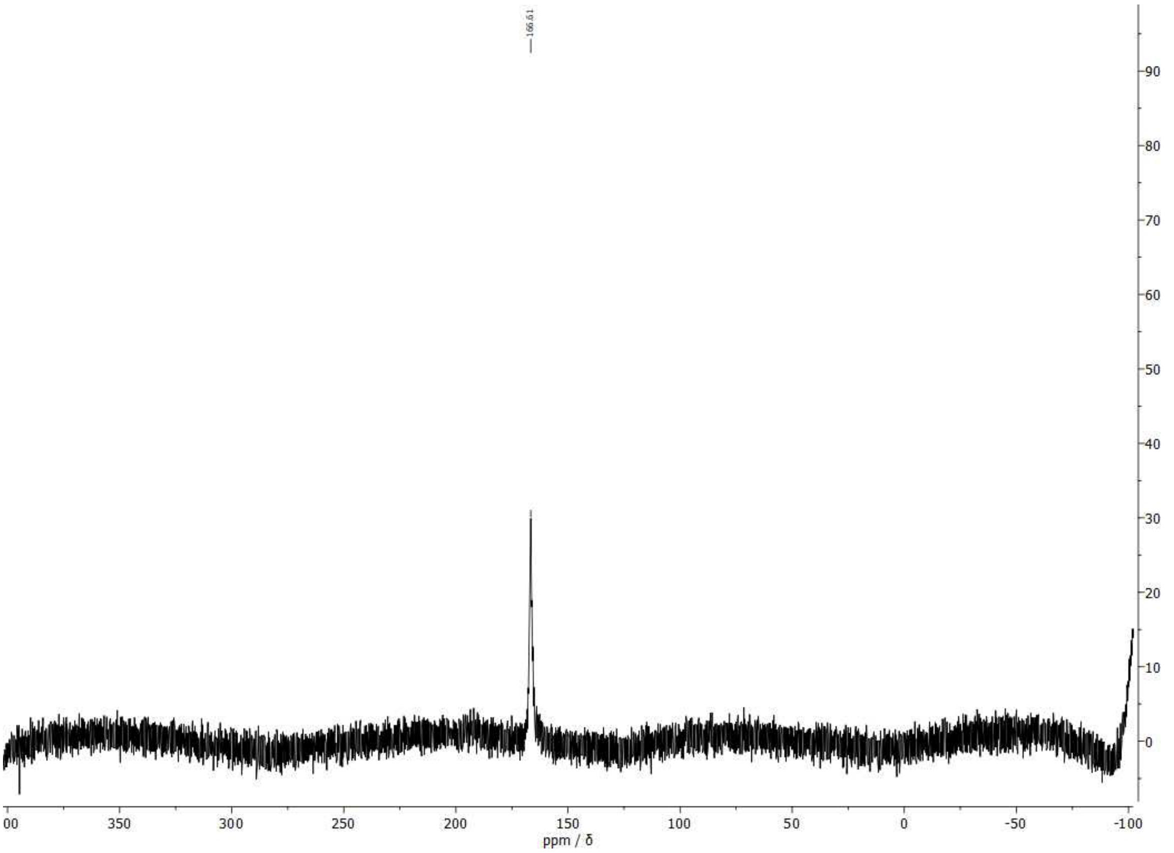

Figure S35. ^{77}Se NMR spectrum of **10** in DMSO-d_6.

Stability testing via ^{1}H, ^{31}P and ^{77}Se-NMR spectroscopy

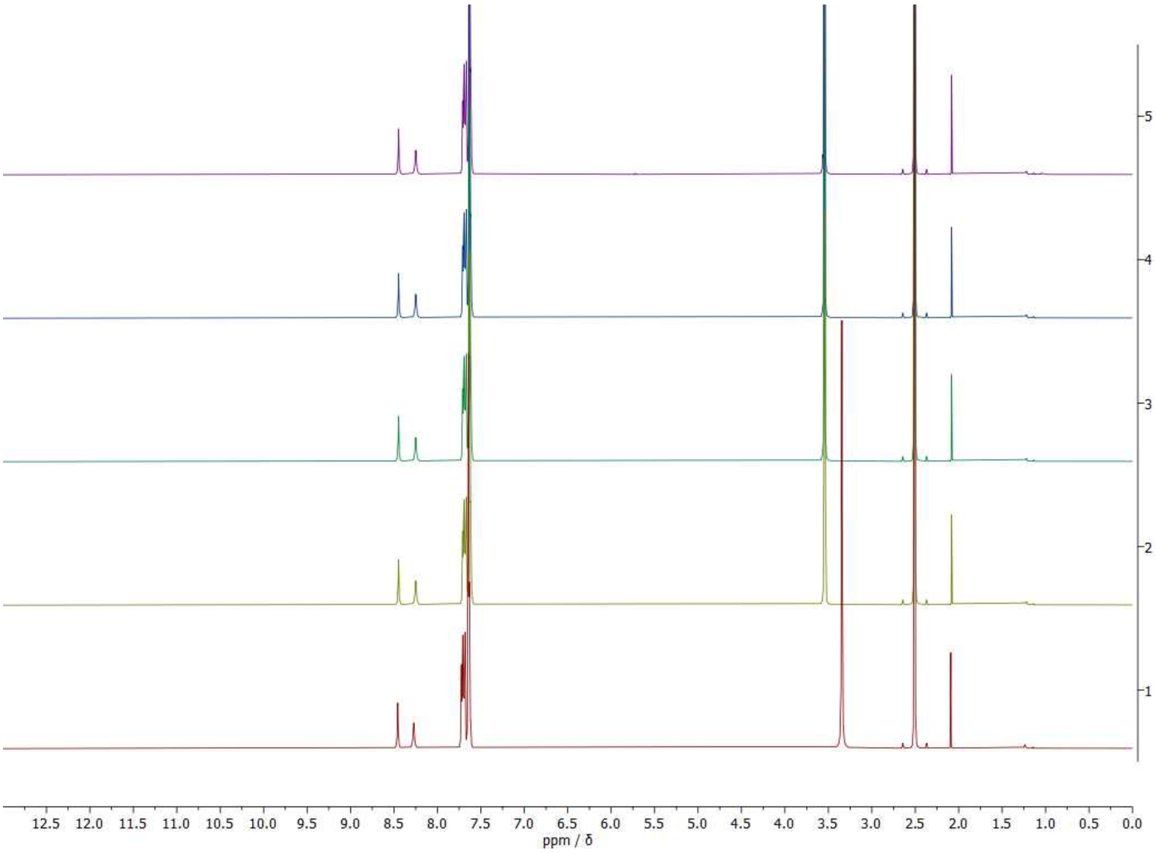

Figure S36. ^{1}H-NMR spectrum of **1** in DMSO-d_6. Addition of 5% D_2O after 0 h (yellow), 24 h (green), 48h (blue) and 72 h (purple).

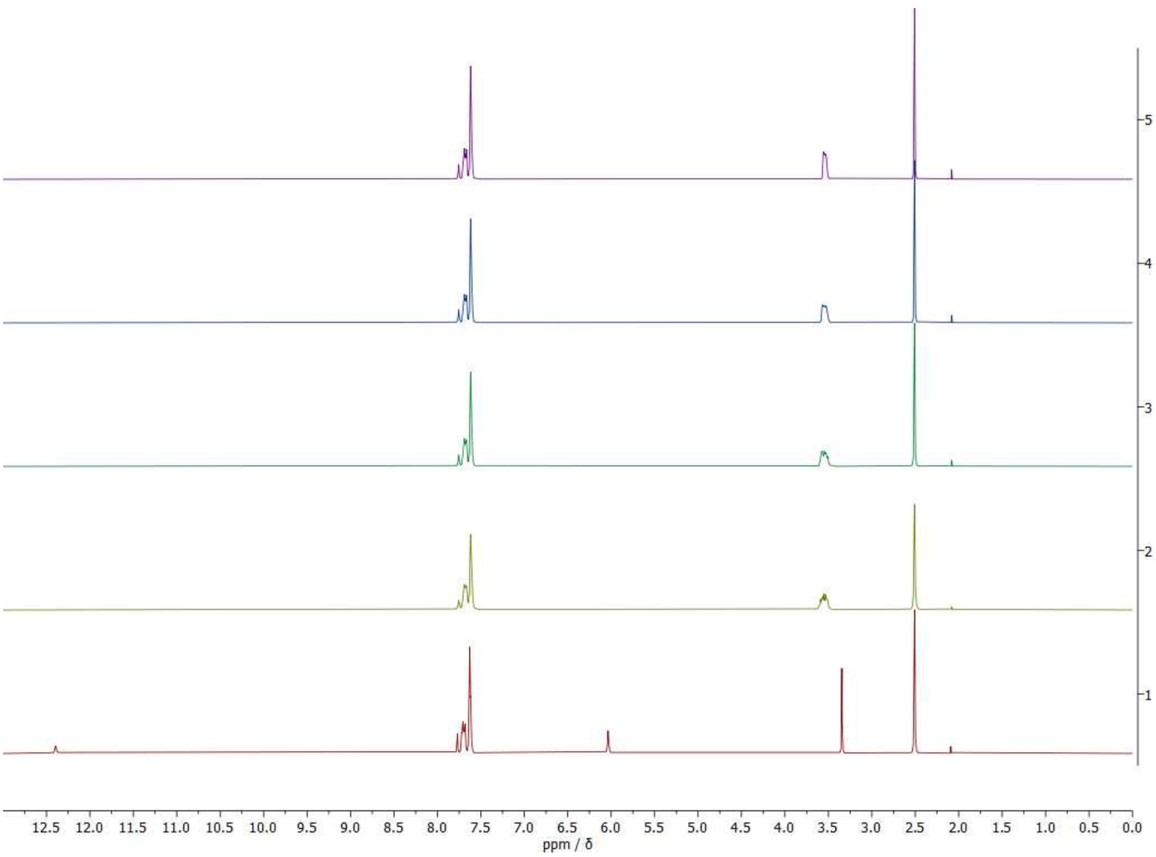

Figure S37. ^{1}H-NMR spectrum of **2** in DMSO-d$_6$. Addition of 5% D_2O after 0 h (yellow), 24 h (green), 48 h (blue) and 72 h (purple).

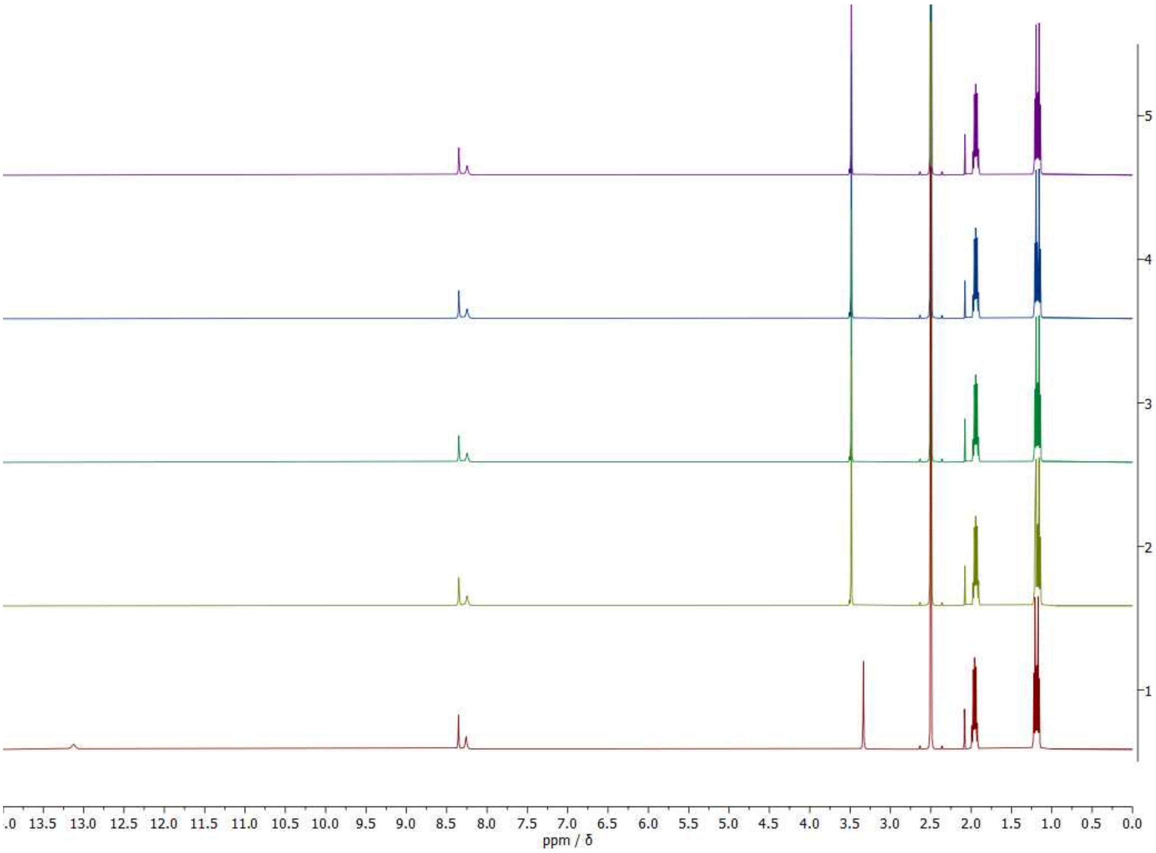

Figure S38. ^{1}H-NMR spectrum of **3** in DMSO-d$_6$. Addition of 5% D_2O after 0 h (yellow), 24 h (green), 48 h (blue) and 72 h (purple).

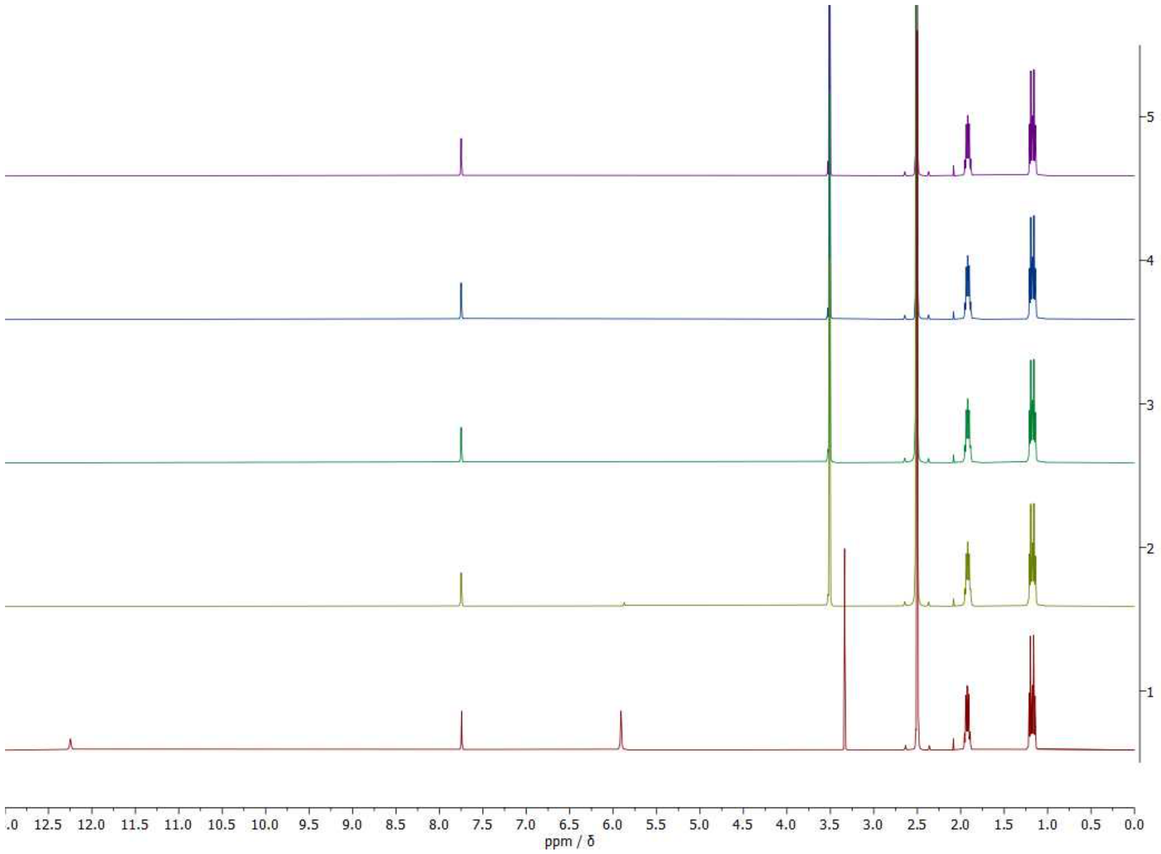

Figure S39. ^{1}H-NMR spectrum of **4** in DMSO-d_6. Addition of 5% D_2O after 0 h (yellow), 24 h (green), 48 h (blue) and 72 h (purple).

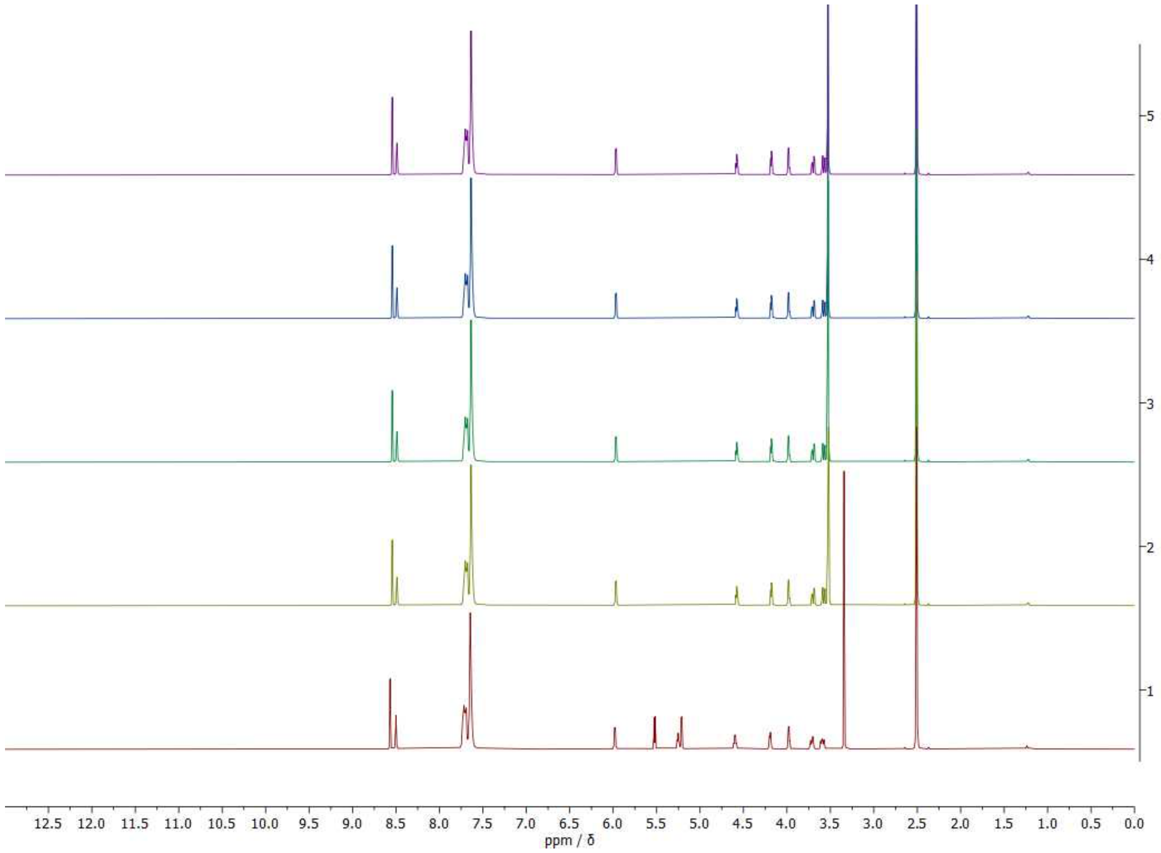

Figure S40. ^{1}H-NMR spectrum of **5** in DMSO-d_6. Addition of 5% D_2O after 0 h (yellow), 24 h (green), 48 h (blue) and 72 h (purple).

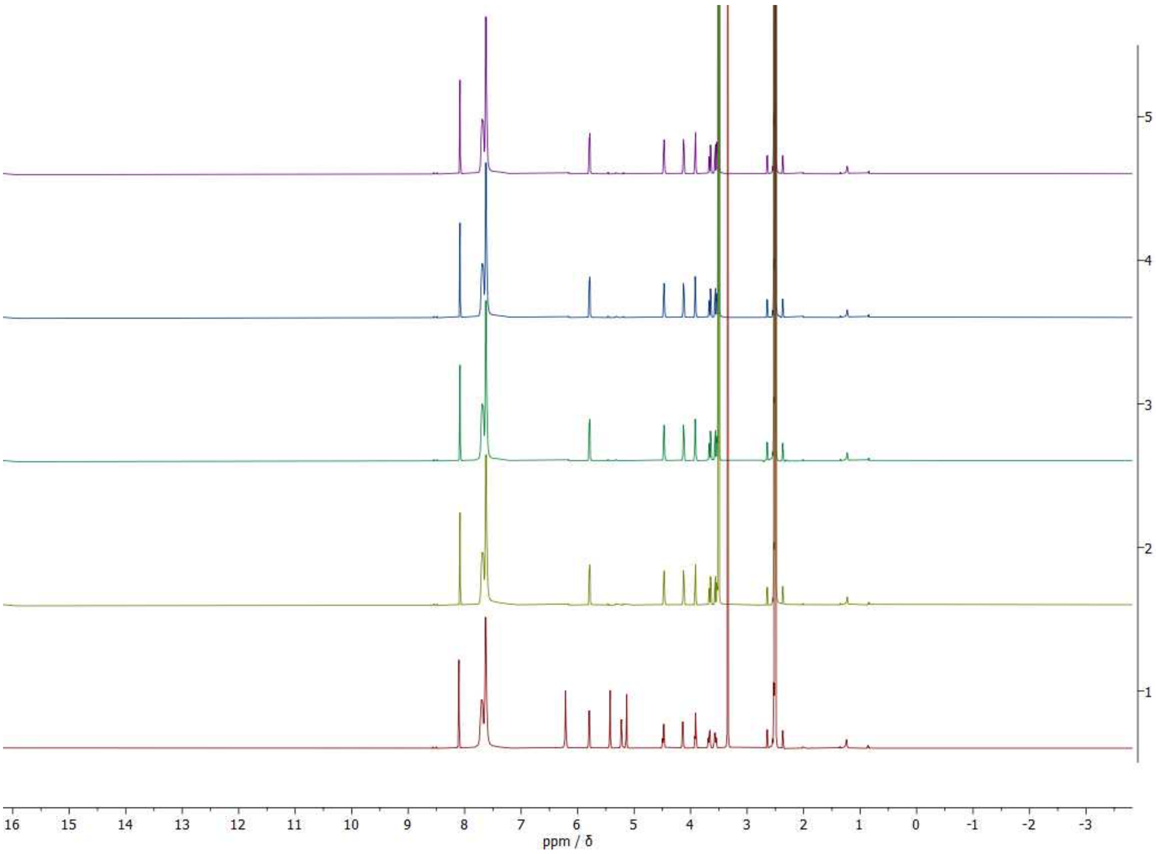

Figure S41. ^{1}H-NMR spectrum of **6** in DMSO-d$_6$. Addition of 5% D_2O after 0 h (yellow), 24 h (green), 48 h (blue) and 72 h (purple).

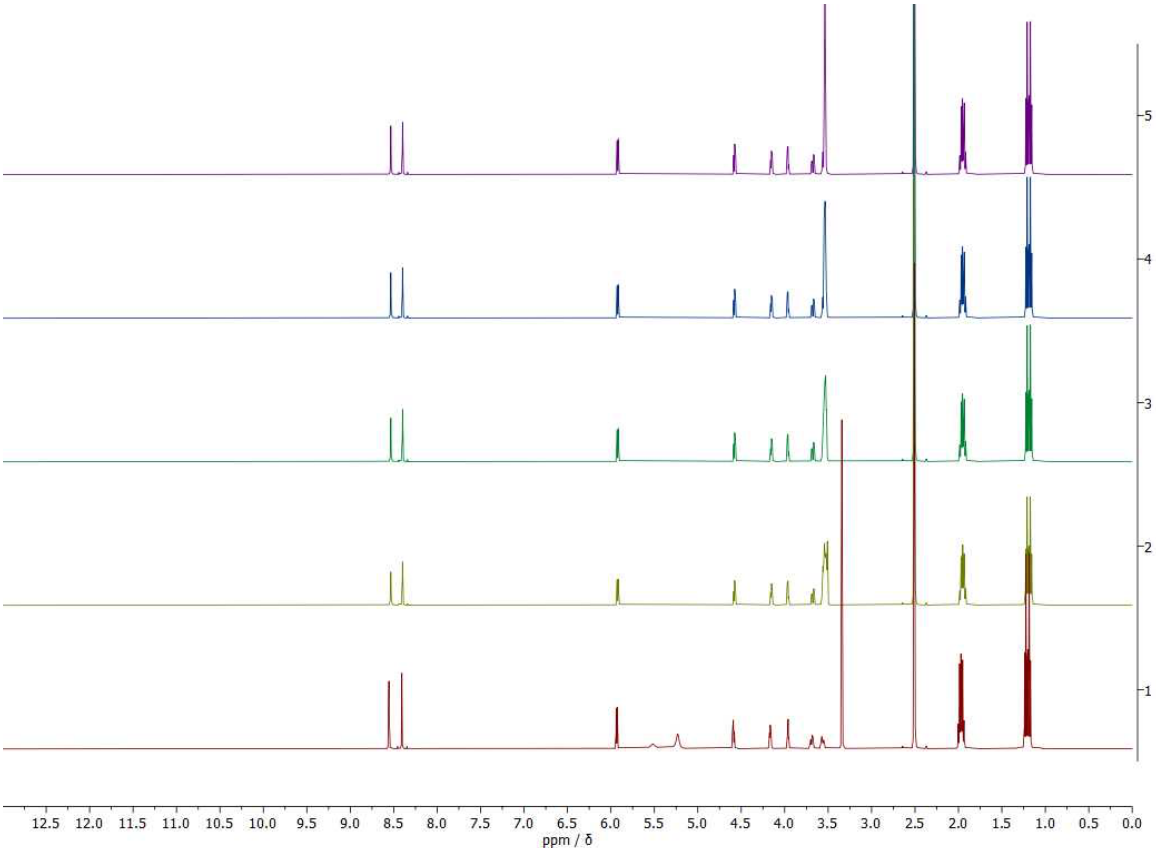

Figure S42. ^{1}H-NMR spectrum of **7** in DMSO-d$_6$. Addition of 5% D_2O after 0 h (yellow), 24 h (green), 48 h (blue) and 72 h (purple).

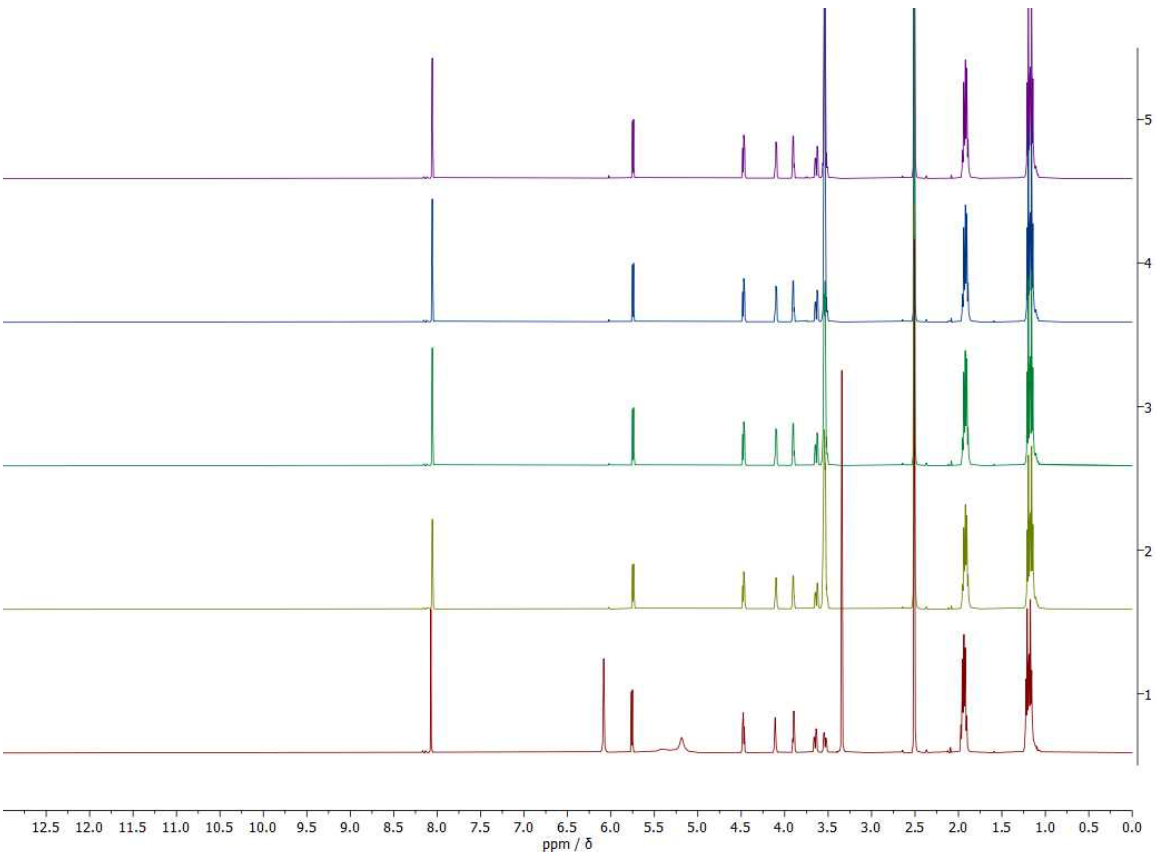

Figure S43. ^{1}H-NMR spectrum of **8** in DMSO-d$_6$. Addition of 5% D_2O after 0 h (yellow), 24 h (green), 48 h (blue) and 72 h (purple).

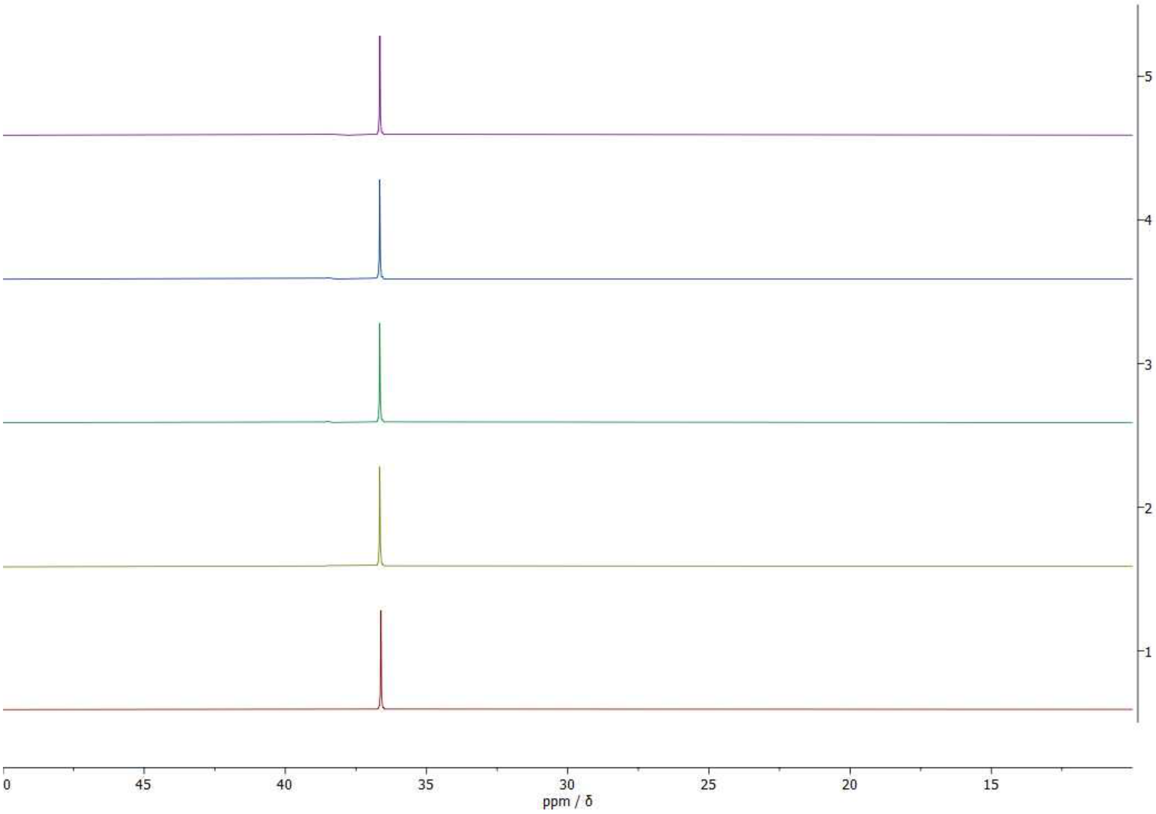

Figure S44. ^{31}P-NMR spectrum of **4** in DMSO-d$_6$. Addition of 5% D_2O after 0 h (yellow), 24 h (green), 48 h (blue) and 72 h (purple).

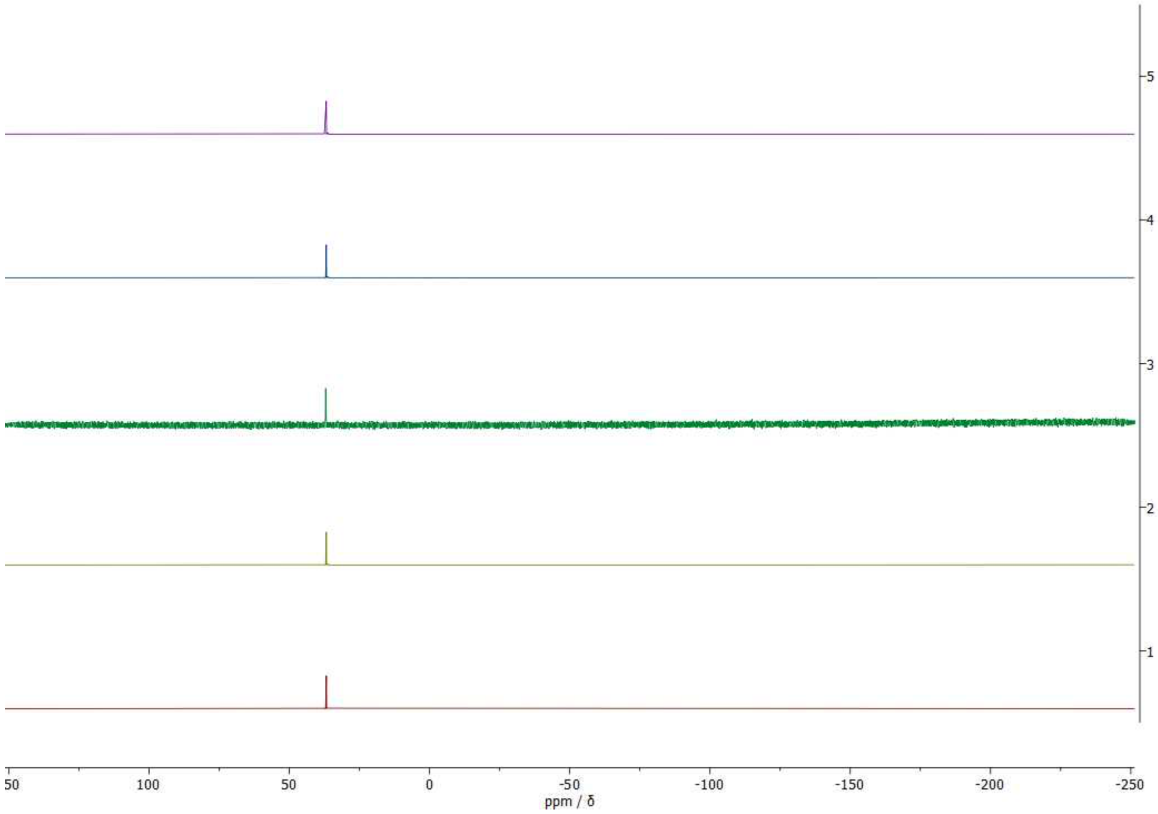

Figure S45. ^{31}P-NMR spectrum of **7** in DMSO-d$_6$. Addition of 5% D_2O after 0 h (yellow), 24 h (green), 48 h (blue) and 72 h (purple).

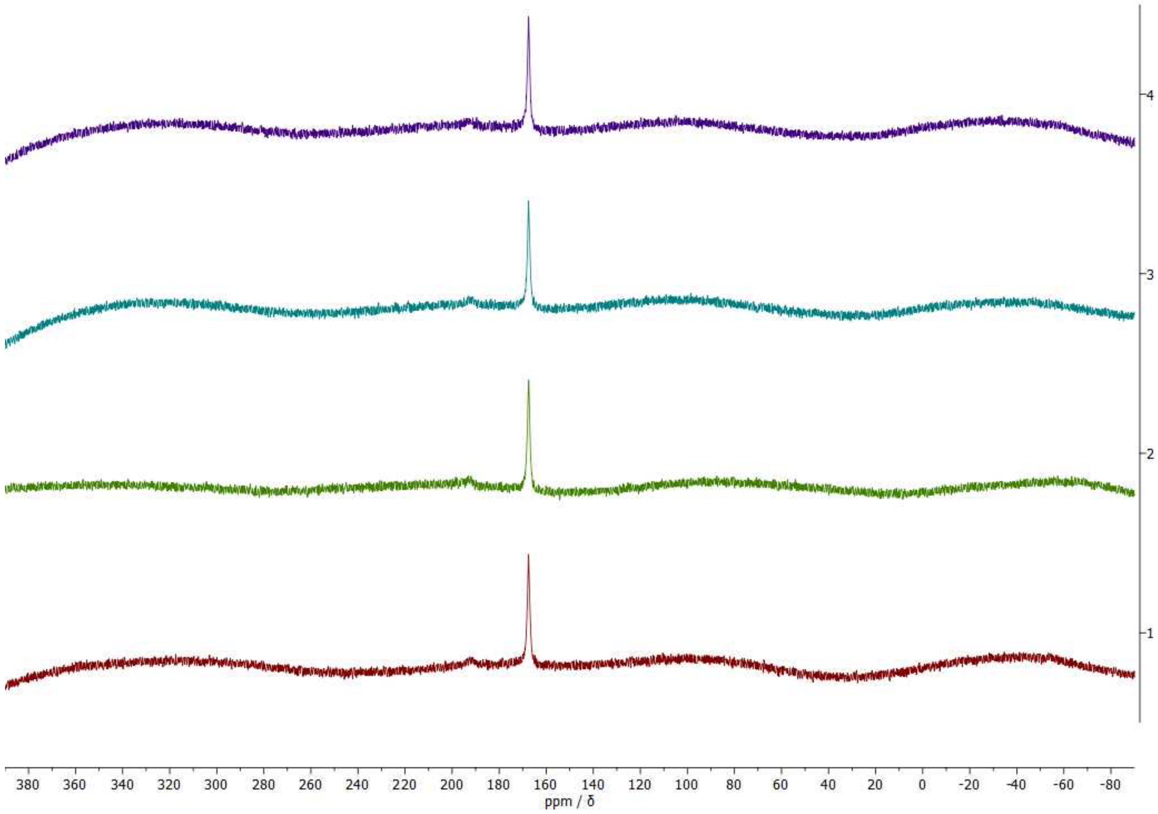

Figure S46. ^{77}Se-NMR spectrum of **10** in DMSO-d$_6$ + 5% D_2O after 0 h (red), 24 h (green), 48 h (blue) and 72 h (purple).

Stability testing via UV/Vis spectroscopy

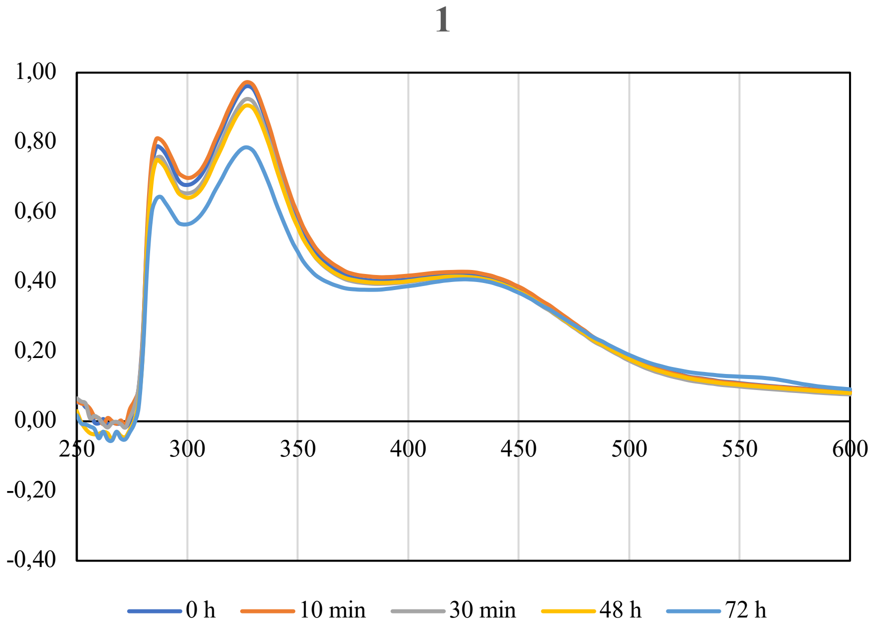

Figure S 47. UV/Vis spectra of complex **1** in EMEM with subtracted background over time.

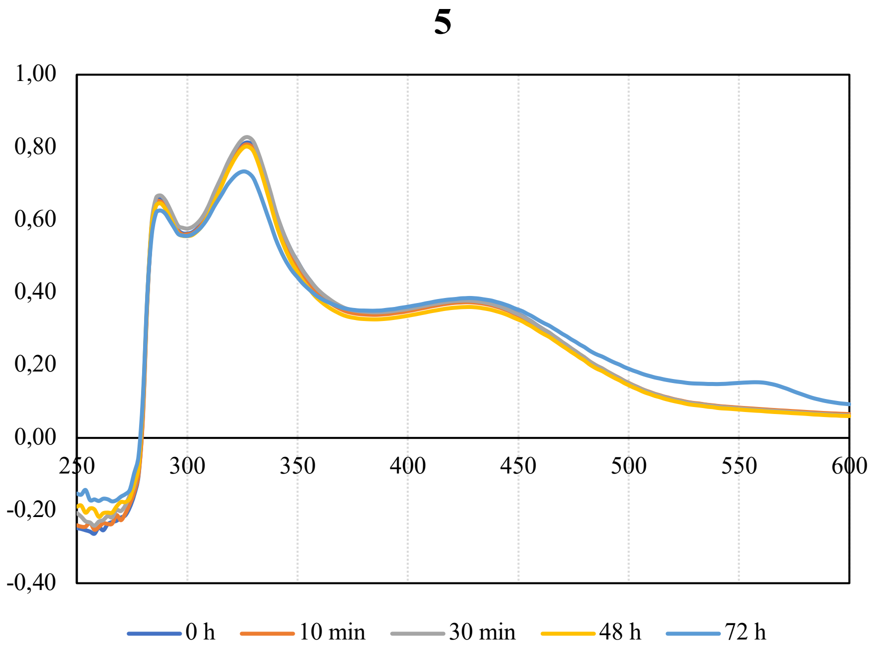

Figure S 48. UV/Vis spectra of complex **5** in EMEM with subtracted background over time.

HPLC Chromatograms

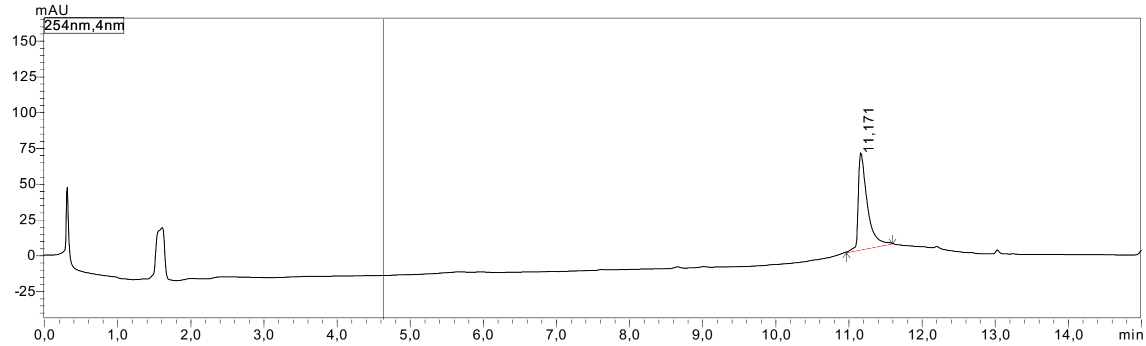

Figure S49. Chromatogram of **1**. Method: 10% → 97% MeCN in H_2O + 0.1% HCOOH, flow: 1.0 ml/min.

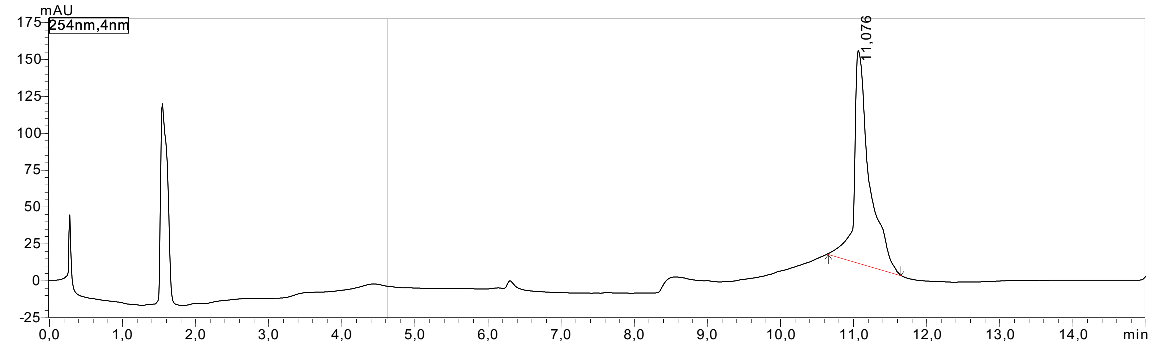

Figure S50. Chromatogram of **2**. Method: 10% → 97% MeCN in H_2O + 0.1% HCOOH, flow: 1.0 ml/min.

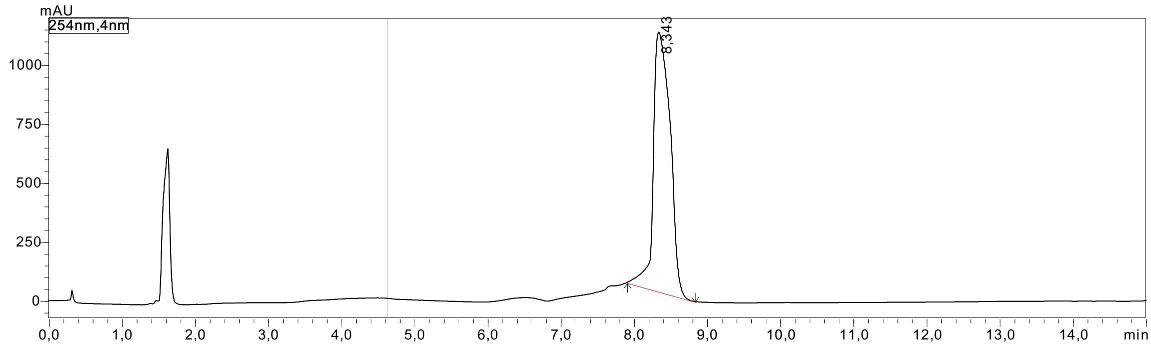

Figure S51. Chromatogram of **3**. Method: 10% → 97% MeCN in H_2O + 0.1% HCOOH, flow: 1.0 ml/min.

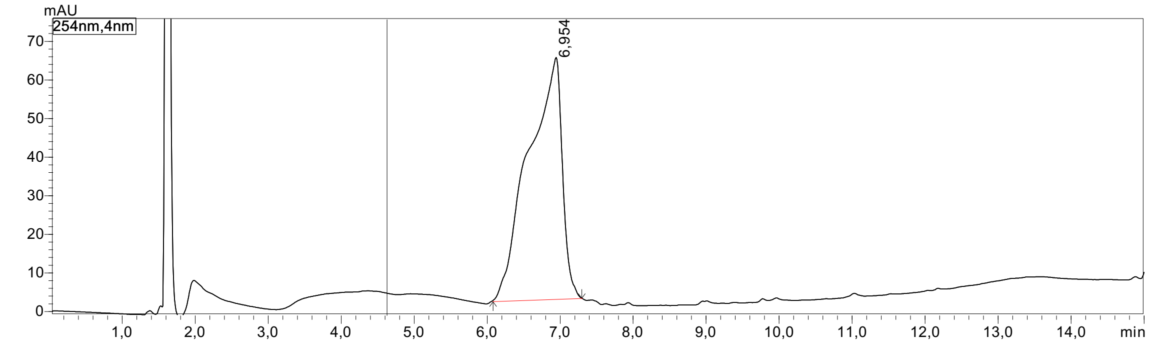

Figure S52. Chromatogram of **4**. Method: 10% → 97% MeCN in H_2O + 0.1% HCOOH, flow: 1.0 ml/min.

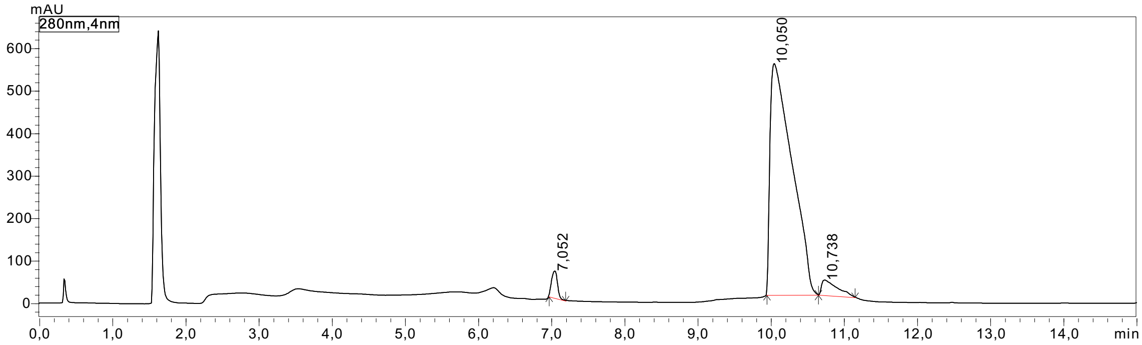

Figure S53. Chromatogram of **5**. Method: 10% → 97% MeCN in H_2O + 0.1% HCOOH, flow: 1.0 ml/min.

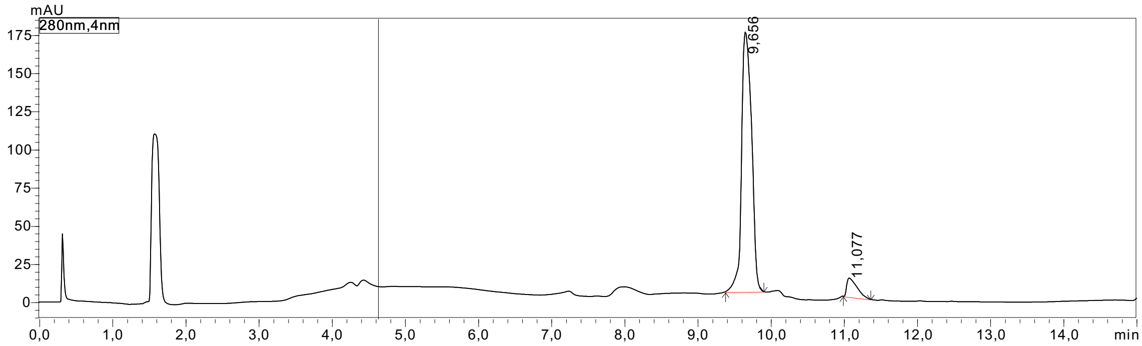

Figure S54. Chromatogram of c **6**. Method: 10% → 97% MeCN in H_2O + 0.1% HCOOH, flow: 1.0 ml/min.

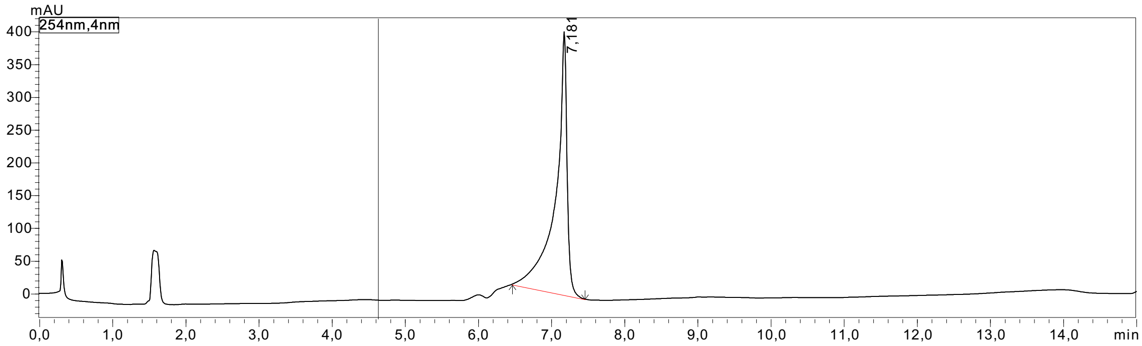

Figure S55. Chromatogram of **7**. Method: 10% → 97% MeCN in H_2O + 0.1% HCOOH, flow: 1.0 ml/min.

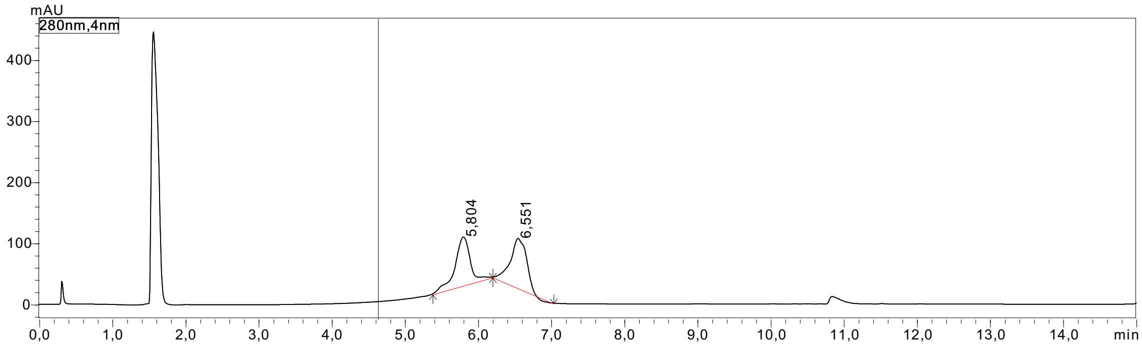

Figure S56. Chromatogram of **8**. Method: 10% → 97% MeCN in H_2O + 0.1% HCOOH, flow: 1.0 ml/min.

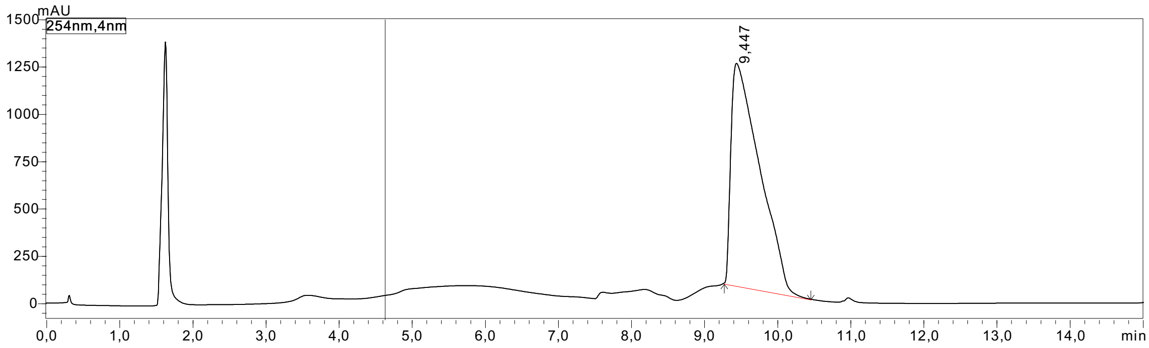

Figure S57. Chromatogram of complex **9**. Method: 10% → 97% MeCN in H_2O + 0.1% HCOOH, flow: 1.0 ml/min.

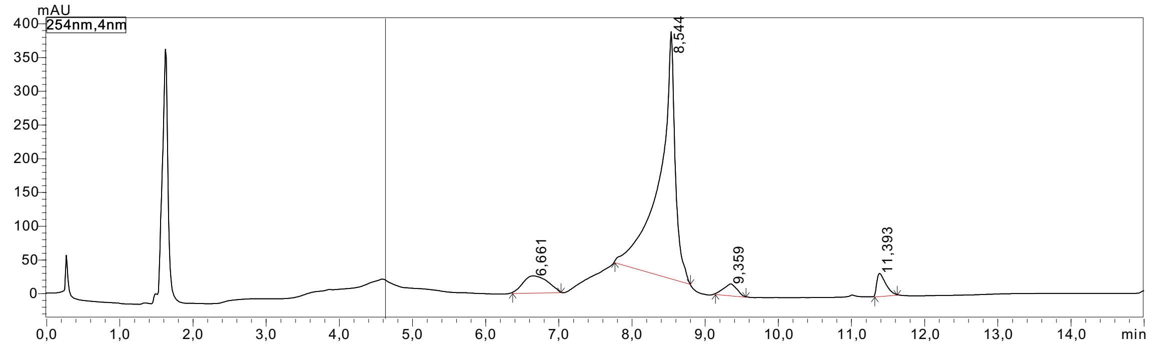

Figure S58. Chromatogram of complex **10**. Method: 10% → 97% MeCN in H_2O + 0.1% HCOOH, flow: 1.0 ml/min.
